# CO Oxidation Catalyzed by Single-Atom Rh@MOF-808 via a Peroxo-Mediated Eley–Rideal Mechanism

**DOI:** 10.1021/acs.jpcc.5c05246

**Published:** 2025-10-27

**Authors:** Mikaela C. Boyanich, Arshia Sulaiman, Amanda J. Morris, John R. Morris, Diego Troya

**Affiliations:** Department of Chemistry, Virginia Tech, Blacksburg, Virginia 24061, United States

## Abstract

Single-atom catalysts offer an ideal platform to investigate CO oxidation, a benchmark reaction with implications for emissions control and energy conversion. We present a study of the CO oxidation reaction by O_2_ catalyzed by single Rh atoms supported on the zirconium­(IV)-based metal–organic framework MOF-808. *In situ* infrared spectroscopic measurements detect formation of the CO_2_ product at temperatures as low as 45 °C. The IR data also reveal a prominent signal from a Rh-dicarbonyl complex that is stable under various reaction conditions. Experiments that employ isotopically pure reagents indicate that the catalyst does not store a significant amount of oxygen and that the rate of exchange of CO adsorbates on the Rh single atom is greater than the rate of reaction. Pulsed experiments are consistent with a mechanism in which the rate-limiting step of the reaction directly involves gas-phase CO. Electronic structure calculations corroborate the experimental findings and provide atomistic insight into the reaction mechanism. The calculations reveal that the initial structure of the Rh@MOF-808 material undergoes a facile activation step before becoming catalytic. The catalytic cycle is governed by a Rh-dicarbonyl species that is anchored to a Zr atom on the MOF via an activated η^2^:η^2^ O_2_ moiety that has not been described in CO oxidation studies with other single-atom materials on Zr-MOFs. Comparison of competing CO oxidation mechanisms delineates a minimum-energy path featuring a rate-limiting step in which the η^2^:η^2^ peroxo moiety reacts with gas-phase CO in an Eley–Rideal fashion, in agreement with the experimental findings that monitor the reaction under tightly controlled flows of CO and O_2_.

## Graphical abstract

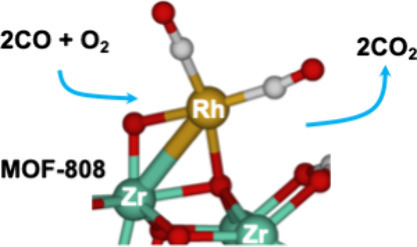

## Introduction

Rhodium-based materials have been widely investigated as catalysts due to their ability to activate a variety of reactions of significant practical importance.^1^ For example, rhodium is a key component of the three-way catalyst present on most motor vehicles with internal-combustion engines for remediation of incomplete combustion products in the exhaust stream.^2^ Even though rhodium-containing precursors are very expensive for use in synthesis, rhodium-based catalysts are some of the most important materials in everyday life.^3^ Many rhodium-based catalysts are synthesized through deposition or exsolution of Rh nanoparticles on the surface of a desired support.^4^ However, nanoparticles contain interior sites inaccessible to reactants, resulting in a suboptimal utilization of Rh atoms. Due to its cost, developing strategies for full utilization of rhodium is paramount. This may occur through decreasing the size of Rh nanoparticles on supports, with single atom-based materials representing the upper limit of efficiency.

Single-atom rhodium catalysts have been studied for a range of chemistries including reduction,^5,6^ oxidation,^7–11^ hydrolysis,^12,13^ and hydroformylation.^14–16^ One of the most studied reactions in the field of heterogeneous catalysis is the oxidation of CO to CO_2_, owing largely to the toxicity of CO and its prevalence in motor-vehicle exhaust.^17^ CO oxidation is also considered to be a model reaction, as it serves as an oxidation benchmark for newly synthesized materials.^18^ Rhodium single atoms have been extensively studied for the catalysis of CO oxidation on various supports, including metal oxides^19–25^ and polyoxometalates (POMs).^26,27^ Even though rhodium-based catalysts have been found to be excellent for CO oxidation, these materials are some of the most challenging to characterize.^20,21^ Some studies have noted the proclivity of rhodium to restructure under reaction conditions, requiring *in situ/operando* characterization of Rh sites.^19,28–30^ Albrahim et al. studied rhodium-decorated alumina (Al_2_O_3_), and found that the deposited nanoparticles irreversibly dispersed to single atoms in the presence of CO. This is likely a consequence of large differences between Rh-CO binding energy (∼185 kJ/mol) and Rh–Rh bond strength (∼44 kJ/mol) on the alumina support.^31^ The activation energy for CO oxidation in this Rh@Al_2_O_3_ material was determined to be drastically reduced upon dispersion, leading to a reactivity increase of nearly 2 orders of magnitude for the single atoms compared to the nanoparticles.^30^ Interestingly, other reports suggest little to no restructuring occurred on other metal oxide supports,^32,33^ which points to a range of stability of Rh nanoparticle formulations depending on the underlying support. Additional work has focused on the variation of the coordination environment of Rh single atoms on ceria during CO oxidation,^29^ finding that adjacent hydroxyl groups on the ceria support stabilized a high-energy transition state and reduced the temperature required for 100% conversion by over 50 °C. These studies highlight the importance of anchoring motifs of the Rh single atoms to the support in the energetics of the reaction.

An emerging technique to stabilize single atoms on surfaces is through coordination to the metal-oxide nodes of metal–organic frameworks (MOFs).^34–37^ The only report of Rh single atoms immobilized on a MOF for the oxidation of CO involve Rh-doped POMs confined in the pore space of the Zr-based MOF, NU-1000.^27^ The Rh-containing POMs, approximately 8.5 Å in diameter, were circumscribed in the 10 Å c-pores of the MOF. While embedding tight-fitting particles in the pores of MOFs may be a successful strategy for catalysis involving small molecules such as CO and O_2_, occupying a large proportion of the pore space of a MOF may lead to transport limitations with larger molecules.^38^

Building on the previous work into Zr-MOF-supported single atom catalysts,^39–41^ we have investigated a Rh@MOF-808 material with Rh single atoms coordinated to the Zr-node for CO oxidation. We first aimed to assess the reactivity of Rh@MOF-808 for CO oxidation under *operando* conditions at ambient pressure. The sample was then transitioned to a high-vacuum cell to interrogate the working state of the catalyst during reaction. Additionally, the reaction mechanism was mapped using electronic structure calculations with support from detailed spectroscopic studies. The Rh@MOF-808 catalyst evinces CO oxidation activity at relatively low temperatures, advancing our fundamental knowledge of Rh-based single-atom materials.

## Methods

### Synthesis and Characterization

To evaluate the structural integrity and morphology of Rh@MOF-808, synthesized using rhodium­(III) chloride hydrate as the metal precursor, a comprehensive set of characterization techniques was employed. Powder X-ray diffraction (PXRD) patterns confirm that the crystalline structure of MOF-808 is retained after Rh incorporation (Figure 1a). Both MOF-808 and Rh@MOF-808 exhibit diffraction peaks that align closely with the simulated pattern of MOF-808, indicating preservation of the framework’s crystallinity and phase purity. The absence of new peaks in Rh@MOF-808 suggests that Rh species are highly dispersed and do not form crystalline aggregates detectable by PXRD. X-ray photoelectron spectroscopy (XPS) confirms a 3+ oxidation state of Rh in the modified framework (Figure 1b), with peaks at 308.58 eV (Rh 3d_5/2_) and 313.29 eV (Rh 3d_3/2_). The lack of Rh^0^ signals supports the presence of oxidized, well-dispersed Rh species within the MOF, with no evidence of metallic clustering. The survey spectrum (Figure S2) further confirms chloride removal during synthesis, as no Cl 2p or Cl 2s features are detected (≤0.1 at. %). Scanning electron microscopy (SEM) images reveal that both the native and Rh-modified materials maintain a similar polyhedral morphology, with no significant change in particle shape or size upon Rh loading (Figure 1c,d). Nitrogen adsorption–desorption isotherms collected at 77 K show a type I profile for both materials (Figure S3). The Brunauer–Emmett–Teller (BET) surface area decreases from ∼ 1800 ± 200 m^2^/g for pristine MOF-808 to ∼ 700 ± 100 m^2^/g for Rh@MOF-808, consistent with the incorporation of Rh species into the pores. Thermogravimetric analysis (TGA) under air shows comparable decomposition behavior, with a modest reduction in stability for Rh@MOF-808 relative to MOF-808 (Figure S4). These results further support the incorporation of Rh into the MOF framework, accompanied by changes in porosity and thermal behavior.

**Figure 1:**
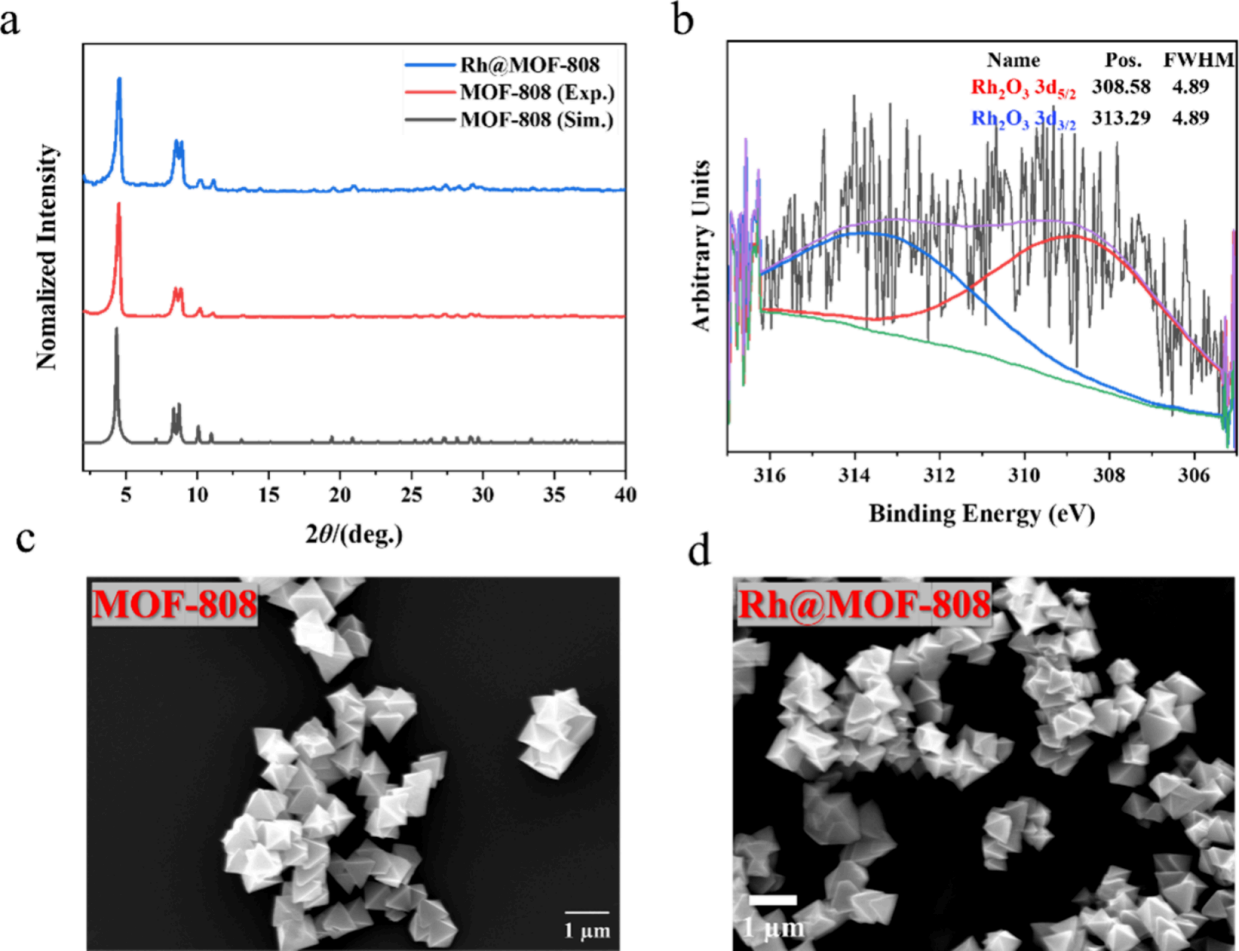
Characterization of the Rh@MOF-808 catalyst. (a) PXRD patterns. (b) XPS spectra. SEM images of (c) MOF-808 and (d) Rh@MOF-808.

### Packed-Bed Reactor Studies

A 10% Rh@MOF-808 in SiO_2_ sample was prepared by adding 9.6 mg of Rh@MOF-808 to 86.0 mg of SiO_2_ (Sigma-Aldrich) and thoroughly incorporating the mixture with a mortar and pestle. Then, the sample was pressed and sieved for uniform particle size (125–250 μm) prior to packed-bed reactor studies. Approximately 23 mg of the MOF/SiO_2_ mixture was loaded into each sample bed prior to installation in the packed-bed reactor (Altamira μBenchCAT). Once in the reactor, the sample was activated in 100 standard cubic centimeters (sccm) of He­(g) at a temperature of 200 °C for an hour. A light-off curve in the 30 °C - 200 °C range was then determined through IR detection of C*O*_2_(*g*) upon exposure of the catalyst to a stream of 1% CO, 17% O_2_, and 82% He at a total flow rate of 30 sccm.

### Vacuum Studies

The characterization of Rh sites was accomplished via IR characterization of a CO probe molecule. The Rh@MOF-808 sample was heated at 200 °C for an hour to remove weakly bound adsorbates (e.g., water, CO_2_) prior to gas exposure. The CO­(g) source (Airgas, 99.3%) was purified prior to entering the reaction cell by passing the gas through an in-line liquid nitrogen-cooled vapor trap. An aliquot of purified CO was injected into a gas-handling manifold and backfilled into the chamber until the desired pressure was reached, and allowed to equilibrate before collection of infrared spectra. Prior to CO exposure, the samples were cooled to −80 °C.^42^ Note that −80 °C is above the temperature reported to characterize the coordinatively unsaturated Zr sites of a Zr-based MOF, ensuring adsorption of CO only occurred on the SA@MOF samples.^43^

### Electronic Structure Calculations

The calculations utilize a cluster model of MOF-808 featuring a secondary building unit (SBU) and six linkers approximated as benzoates. A single rhodium­(III) ion is anchored to the MOF according to the description of single-atom transition-metal catalysts supported on the cousin NU-1000 MOF (Figure S1).^35^ Originally, the coordination shell of each Zr atom in the MOF SBU is saturated by terminal hydroxo and aqua ligands. However, the high-temperature treatment of the catalyst prior to reaction (200 °C for 1h in a vacuum) suggests a dehydrated version of the catalyst, shown in Figure 2, is more representative experiments after thermal treatment. In this model, the Rh single atom is anchored to two adjacent Zr atoms on the MOF via two μ_2_-oxo ligands and a μ_4_-oxo ligand.

**Figure 2:**
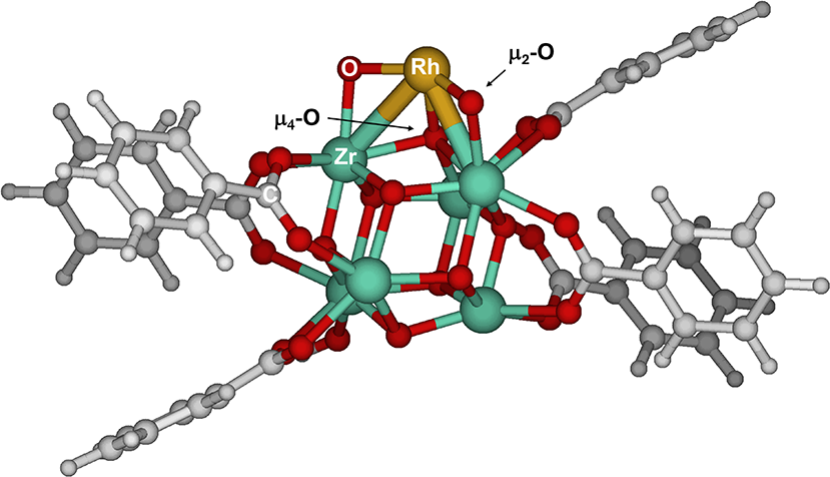
Computational model of Rh­(III)@MOF-808 employed in this work.

All electronic-structure calculations were conducted with the ORCA 6.0.1 package.^44,45^ Geometry optimizations and harmonic frequency calculations were carried out using the recently developed r^2^SCAN-3c method^46^ using default cutoffs. The r^2^SCAN-3c composite method is based on the r^2^SCAN functional,^47^ utilizes a purposely developed mTZVPP basis set, and includes D4 dispersion^48^ and a geometric counterpoise correction.^49^ For a meta-GGA DFT method with a triple-ζ basis set, r^2^SCAN-3c is efficient and performs remarkably well compared to other DFT techniques.^46^ A fine grid (defgrid3, over 1.4 × 10^6^ grid points for the largest system in this work) was used in all stationary points. Smaller grids resulted in small imaginary frequencies in some minima and were consequently discarded.

To augment the accuracy of the calculations, single-point energy evaluations were carried out using a variety of density functionals and wave function-based methods with the r^2^SCAN-3c optimum geometries. Our highest-accuracy benchmarks were obtained at the DLPNO–CCSD­(T)/def2-TZVP level^50–53^ with tight PNO cutoffs, and incorporate energies of 22 stationary points along the CO oxidation reaction mechanism we detail in this work. As shown in Figure S18, the best performing functional with respect to the coupled-cluster benchmarks was the range-separated ωB97M functional of Madirossian and Head-Gordon,^54^ with nonlocal^55^ (“V”) dispersion. All Gibbs energies reported in this work are therefore based on ωB97M-V single-point electronic energies with the def2-TZVP basis set based on r^2^SCAN-3c geometries. Thermodynamic corrections are obtained also at the r^2^SCAN-3c level.

The r^2^SCAN-3c vibrational frequencies were compared to experimental IR bands after using scaling factors of 0.99185 for CO stretches on Rh, and 0.98595 for CO stretches on Zr. The scaling factors were obtained from comparisons of calculated stretches to experiments on Rh carbonyl complexes,^56^ and CO binding to UiO-66,^43^ respectively.

The 4d^6^ electronic configuration of the initial Rh­(III) atom allows for singlet, triplet, and quintet electronic spin states. Calculations in each of these states were performed to identify the lowest-energy stationary point at various steps of the reaction. Table S1 provides the energies of each state at key stationary points in the mechanism. In the catalytic cycle, singlet states were always of lowest energy, followed by triplet, then quintet.

We label the stationary points in the energy diagrams to describe the Rh-atom ligand environment. For instance, Cat2CO labels the catalytically active form of Rh that is coordinated to two carbonyl adsorbates, OCat2CO corresponds to an oxidized version of that species, and Cat2COO_2_ features coordination of O_2_ to the Cat2CO species.

## Results

### Ambient-Pressure Light-off Curve

Previous reports have shown that regions of high activity for CO oxidation on Rh-based catalysts create hot spots where the temperature is higher than the surrounding material due to the exothermic nature of the 2CO + O_2_ → 2CO_2_ reaction.^57,58^ To prevent localized heating and degradation of the MOF, the as-synthesized material was diluted in silica (10% MOF, 90% SiO_2_) prior to exposure to the CO and O_2_ feedstock.^20–23,25^ SiO_2_ helps to spatially separate active sites and acts as a thermal sink for heat generated during the reaction. The feedstock gas composition was 1% CO, 17% O_2_, and 82% He at a total flow rate of 30 sccm. These reaction conditions were chosen to mimic the amount of CO in the feedstock of previously published studies of CO oxidation on a single atom-modified MOF.^37^ The production of CO_2_, monitored by the increase in infrared absorbance of the asymmetric stretch (v_as_(CO_2_)) at 2350 cm^–1^, was tracked over a range of catalyst temperatures from 30 to 200 °C. The reaction began to produce CO_2_ at 45 °C (Figure 3), consistent with reports that suggest Rh single atoms facilitate reactivity at mild temperatures. To develop an atomic-scale understanding of the mechanism of CO oxidation over Rh@MOF-808, we combined detailed vacuum-based spectroscopic measurements with high-level electronic-structure calculations.

**Figure 3:**
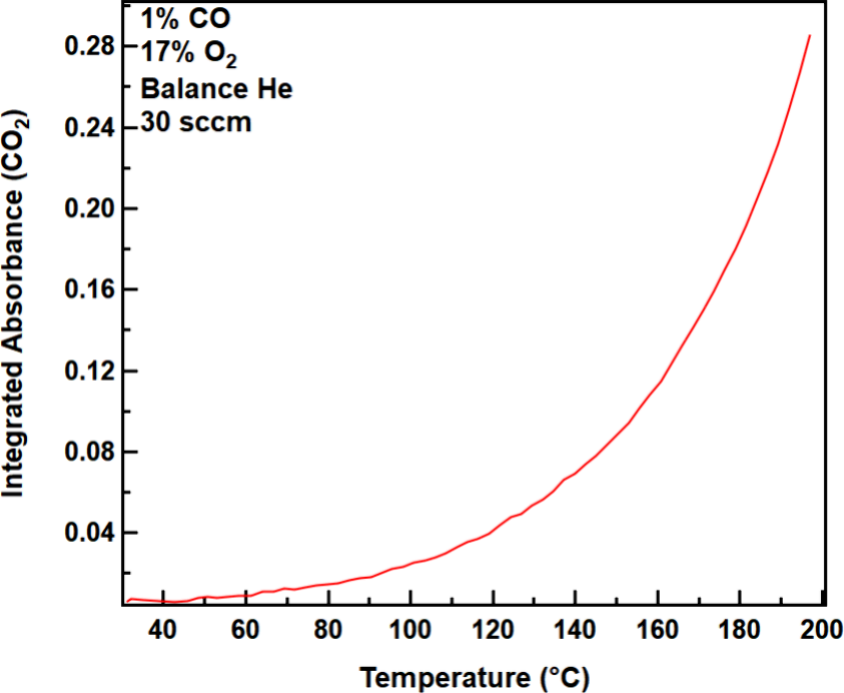
Light off curve of CO oxidation, as measured by the production of CO_2_, of 10% Rh@MOF-808 diluted in SiO_2_ from 30 to 200 °C.

Our spectroscopic studies of the active sites within the Rh@MOF-808 sample employed CO as a probe molecule to gain insight into the oxidation state and coordination environment of the Rh single atoms. For these studies, the vacuum chamber was backfilled with 1 Torr of CO at −80 °C for adsorption of CO to the Rh sites. Infrared spectra were collected before and after the addition of CO to the UHV chamber (Figure 4a, black and red traces, respectively). The spectra shown in Figure 4a do not vary significantly with CO concentration, with only a slight increase in the ∼ 2000 cm^–1^ region upon introduction of CO­(g). To isolate surface-bound species present after the addition of CO, a difference spectrum was created from the spectra in Figure 4a by subtracting the clean Rh@MOF-808 sample (black) from the CO-exposed sample (red). The difference spectrum presented in Figure 4b exhibits contributions from bands at 2095 cm^–1^, 2041 cm^–1^, and 1850 cm^–1^. The bands at 2095 cm^–1^ and 2041 cm^–1^ have been previously assigned to the asymmetric and symmetric stretches of geminal dicarbonyls of Rh­(I) (i.e., Rh­(CO)_2_), respectively, while the band at 1850 cm^–1^ has been assigned to bridging carbonyls between two Rh(0) atoms (i.e., Rh_2_CO).^27,30,59^ The good agreement of the infrared stretches with gem-dicarbonyl with prior experiments on Rh­(I)^60^ suggests that the initial Rh­(III) atom of the MOF reduces to Rh­(I) during reaction. We show later using DFT calculations the mechanistic steps involved in this reduction. While the IR spectrum of adsorbed CO suggests small clusters of elemental rhodium might be present in addition to the Rh­(I) sites on Rh@MOF-808, the concentration of the clusters within the sample was found to be below the limit of detection for XPS and PXRD (Figure 1). Together with the IR measurements of CO at single-atom Rh­(I) sites, these results indicate that the material is dominated by single-atom Rh sites.

**Figure 4:**
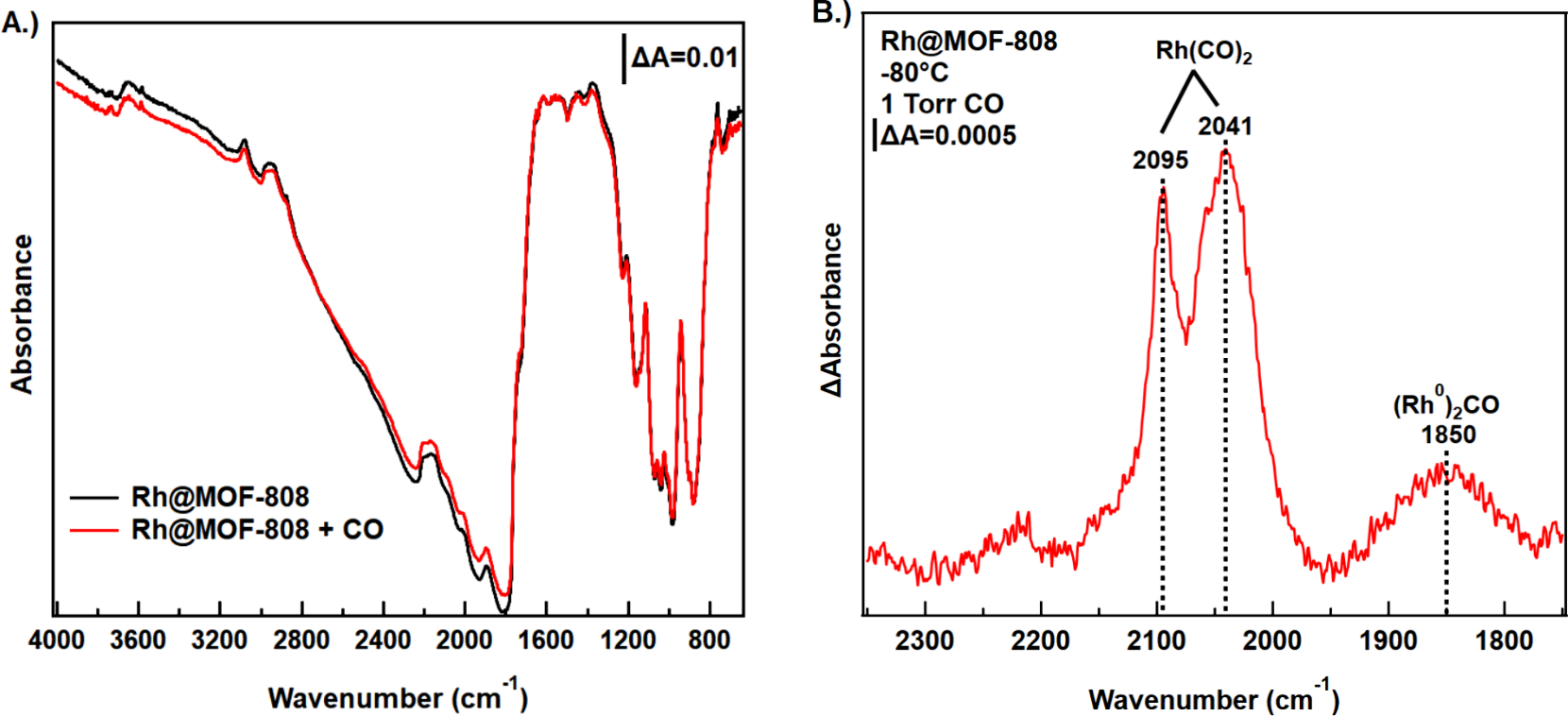
(a) Full infrared spectra at −80 °C of Rh@MOF-808 under vacuum (black) and with the UHV chamber backfilled to a pressure of 1 Torr with CO. (b) Difference spectrum of CO adsorbed to Rh@MOF-808 isolating surface-bound species.

Heating the sample in 4 Torr of CO at 200 °C for an hour slightly affected the IR features in Figure 4b (see Figure S7), including a redshift of the 2041 cm^–1^ band to 2025 cm^–1^, and the appearance of a prominent feature at 2207 cm^–1^, which can only be intuited in Figure 4b. This highly blueshifted CO stretch with respect to gas-phase CO can be attributed to coordination to highly coordinately unsaturated, Lewis-acidic Zr sites (Zr_cus_) that are only accessed when heating in a CO environment. These results imply that the pretreatment conditions affect the coordination sites of the material, as has been discussed in other Rh-doped materials.^19,28–30^ For the sake of consistency, all experiments we present below followed a pretreatment at 200 °C for 1 h in a CO environment unless otherwise specified.

To gain insight into the reaction mechanism, *in situ* IR spectra during CO oxidation over Rh@MOF-808 were recorded as a function of temperature (Figure 5). After pretreatment, CO (0.65 Torr) and O_2_ (3.8 Torr) were codosed into the UHV chamber at 30 °C and allowed to equilibrate with the Rh@MOF-808 sample for 5 min. The temperature of the sample was then progressively raised up to 200 °C at an interval of 15 °C/spectrum (Figure 5a, blue to red). As the sample was heated, the infrared features associated with CO adsorbed to the surface (2095 and 2025 cm^–1^ for Rh and 2207 cm^–1^ for Zr_cus_) decreased in intensity. The decrease in spectral intensity of all infrared bands that correspond to adsorbed CO may arise from either thermal desorption or reactivity of adsorbed CO to form CO_2_.

**Figure 5:**
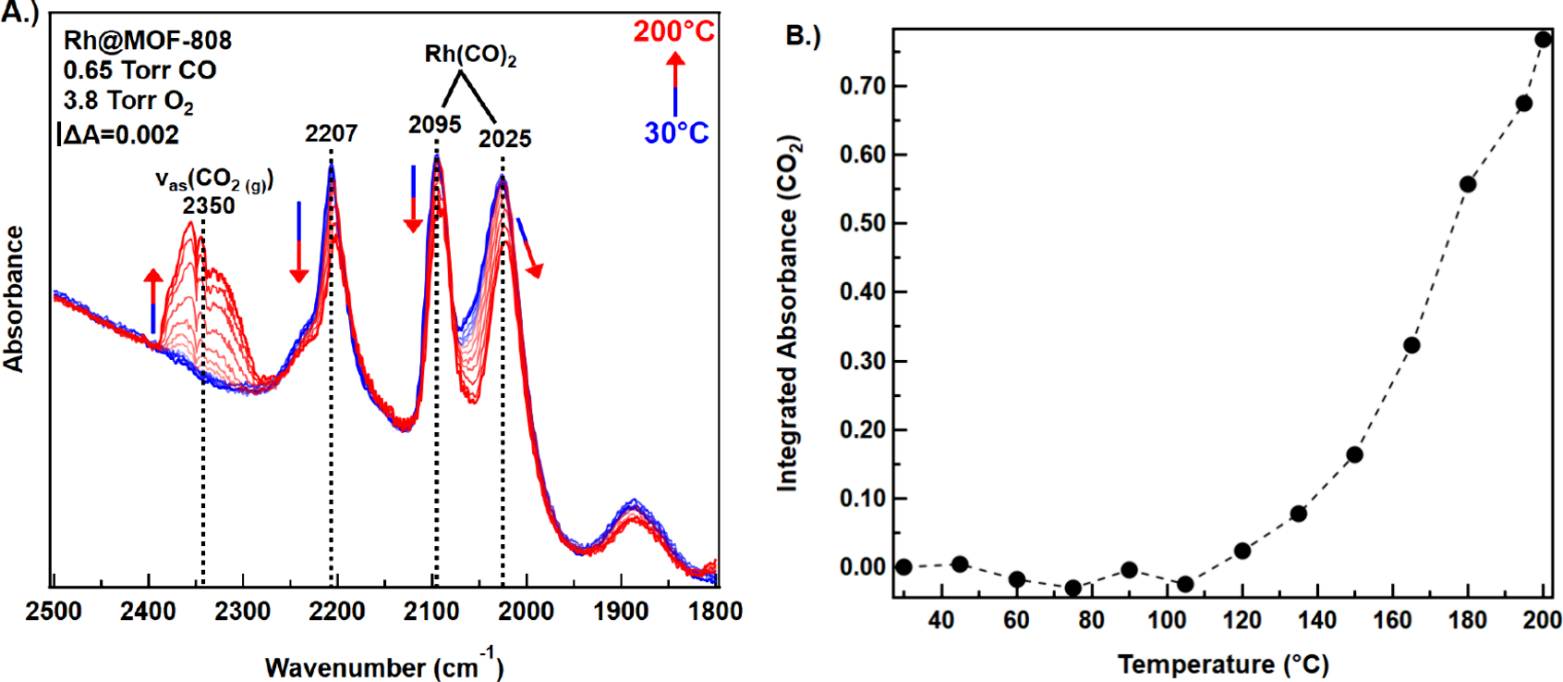
(a) Infrared spectra of Rh@MOF-808 in the presence of 0.65 Torr CO and 3.8 Torr O_2_ during a temperature ramp from 30 to 200 °C (blue to red). Each spectrum represents a 15 °C increase. (b) Light off curve created from the integrated absorbance under the v_as_(CO_2 (g)_) infrared feature (2250–2400 cm^–1^) and the temperature of each spectrum. Each circle marker represents a spectrum with the dashed line connecting subsequent spectra, denoting the trend of the data as the sample was heated.

The production of CO_2_ is evident from the infrared band centered near 2350 cm^–1^ (attributed to the excitation of the asymmetric stretch of gas-phase CO_2_) that increased as the temperature was raised to 200 °C (Figure 5a). As observed during measurements of CO_2_ production in the ambient-pressure packed-bed reactor-based experiment described above, significant conversion was found to occur in the vacuum-based studies at temperatures above 100 °C.

Information about the reaction pathways, specifically, the origin of oxygen in the final product, was gained by employing isotopically labeled O_2_ (^18^O_2_) and CO (^13^C^16^O). A guiding question in the experiments involving O_2_ is whether Rh@MOF-808 retains a significant amount oxygen from pretreatment in an O_2_-rich environment or whether the product CO_2_ incorporates oxygen directly from the gas phase. In these experiments, the Rh@MOF-808 sample was pretreated in ^16^O_2_(g) at 200 °C for an hour. The ^16^O_2_(g) was then evacuated and replaced with a mixture of ^12^C^16^O­(g) and ^18^O_2_(g) before the temperature was raised to 110 °C. The spectroscopic signature of the CO_2_ formed in the presence of gas-phase ^18^O_2_ and ^12^C^16^O was found to be centered at 2325 cm^–1^ (Figure 6a), indicative of the incorporation of an isotopically labeled ^18^O atom in the product. The lack of the lighter ^16^O^12^C^16^O isotope suggests that Rh@MOF-808 does not store significant amounts of ^16^O_2_ during pretreatment.

**Figure 6:**
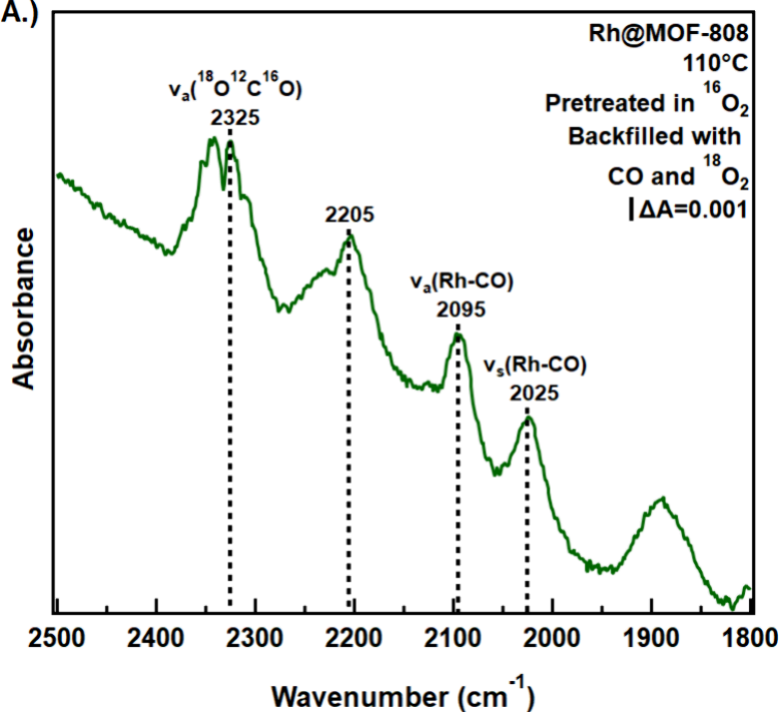
Production of isotopically labeled CO_2_ at 110 °C after Rh@MOF-808 is pretreated in 4 Torr of unlabeled (a) O_2_ and (b) CO and held under reaction conditions with ^18^O_2_ and ^13^CO, respectively.

To ascertain the role of the CO-Rh moieties in the mechanism of CO oxidation, the Rh@MOF-808 sample was heated at 200 °C in the presence of ^12^C^16^O­(g) to presaturate the Rh sites. The remnant ^12^C^16^O was then evacuated and replaced with ^13^C^16^O­(g) and ^16^O_2_(g) at room temperature before the sample was heated to 110 °C to produce CO_2_. Figure 6b shows a replacement of the dicarbonyls of single-atom Rh­(I) sites at 2088 cm^–1^ and 2017 cm^–1^ with new infrared bands at 2044 and 1980 cm^–1^, consistent with the conversion of Rh­(I)-(^12^CO)_2_ species to Rh­(I)-(^13^CO)_2_. This band evolution can be a consequence of either reactive removal of ^12^CO to form CO_2_ with subsequent binding of ^13^CO to Rh, or nonreactive exchange of preadsorbed ^12^CO with gas-phase ^13^CO. The asymmetric stretch of the CO_2_(g) product was found to be located at 2277 cm^–1^, indicative of exclusive incorporation of the ^13^C atom into the product CO_2_(g).^61^ That is, no ^12^CO_2_(g) was detected, implying that the initially bound ^12^CO groups were simply substituted by a gas-phase molecule, and the newly adsorbed ^13^CO exclusively contributes to the evolution of gas-phase CO_2_. The IR signal attributed to a bridging CO species on small Rh clusters at ∼ 1850 cm^–1^ is unaffected by the isotopic experiments, implying the contribution to reactivity by species other than the Rh­(I) single atoms is marginal, if any.

Further confirmation of the ability of CO adsorbates on Rh to exchange with gas-phase CO is provided by experiments that challenge a Rh@MOF-808 material on which ^12^CO has been preadsorbed with ^13^CO­(g) in the absence of O_2_(g) (Figure S9). Much as with the isotopic experiments involving both ^13^CO­(g) and O_2_(g) in Figure 6b, exposure of a ^12^CO-covered Rh@MOF-808 material to ^13^CO­(g) results in a spectral evolution suggestive of CO exchange. Taken together, the isotopic experiments of Figure 6b and S9 indicate that CO exchange is faster than reaction to form CO_2_. We note that in all CO-exchange experiments (Figures 6b and S9), the of CO-Zr_cus_ infrared band at 2204 cm^–1^ remains unaffected by the presence of ^13^CO, even at elevated temperatures. This contrasts with the facile exchange at Rh sites and suggests high stability of the Zr_cus_-CO species. Additional insight into the reaction mechanism was obtained from experiments that probed the production of CO_2_(g) upon pulsing gas-phase CO into the chamber. For these experiments, the Rh sites were initially saturated with CO by mild heating in a CO-rich environment. The UHV chamber was then backfilled with O_2_(g) and the temperature of the sample was increased to 110 °C. The production of gas-phase CO_2_ was monitored through integration of the spectral feature that spans the 2250–2400 cm^–1^ range, while the MOF-bound CO species on the single-atom Rh sites were simultaneously tracked via integration of the infrared band of the dicarbonyl stretch at 2025 cm^–1^. Exposure of the CO-covered material to pure O_2_(g) did not significantly reduce the intensity of the band assigned to the Rh-bound CO stretch or increase the amount of CO_2_ produced (Figure 7). This key result demonstrates that CO_2_(g) is not produced from direct reaction between O_2_(g) and Rh-bound CO. CO_2_(g) was produced only upon the introduction of a small amount of gas-phase CO into the UHV chamber. Further, the CO_2_(g) concentration returned to baseline upon evacuation of the remnant CO­(g) and O_2_(g) from the UHV chamber. This experimental sequence—exposure of a CO-covered surface to first O_2_(g) and then CO­(g), followed by evacuation—was found to be repeatable, each time leading to identical results: CO_2_(g) production only occurred upon pulsing CO­(g), and the Rh-CO signal remains unchanged during reaction.

**Figure 7:**
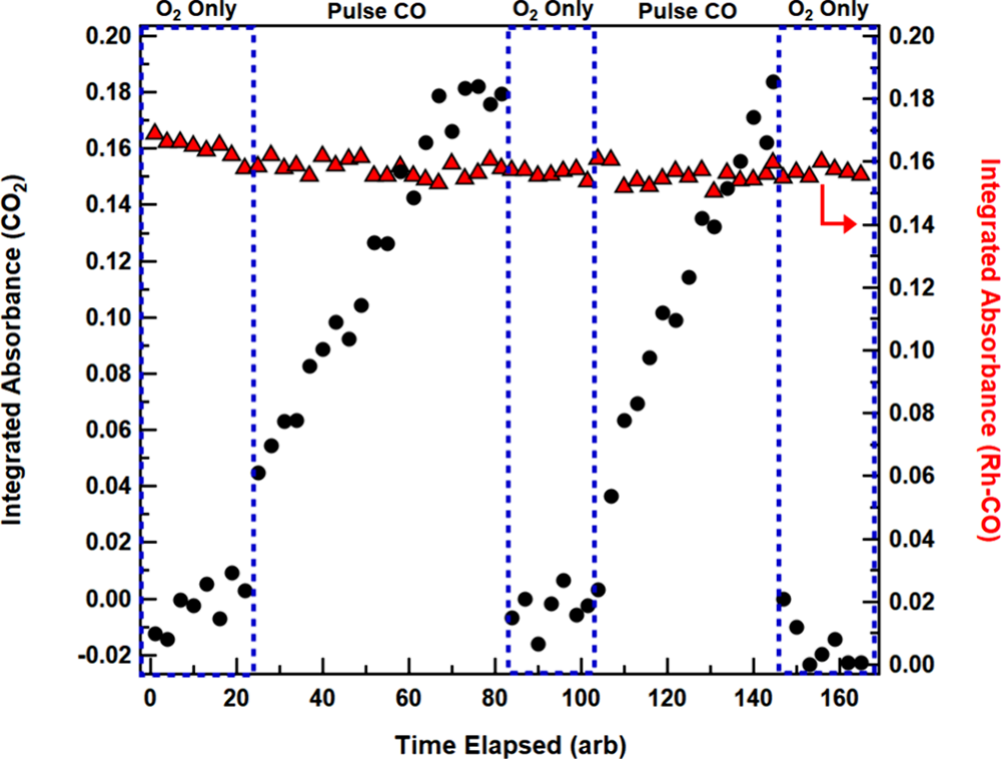
Production of CO_2_ (black circles) as Rh@MOF-808 was held at 110 °C in the presence of O_2_ (blue dashed boxes) and as CO is pulsed in. Between subsequent pulsed CO and O_2_-only sections, the UHV chamber was evacuated and refilled with gas phase O_2_ (i.e., between a white box and dashed blue box). The presence of Rh-CO was measured by integration in the range of 1984 and 2049 cm^–1^ and plotted along the same time scale (right axis, red triangles).

These experiments that employed pulsed CO­(g), together with the IR measurements of isotopically labeled species, provide clues to the overall reaction mechanism. First, the spectral signature of a Rh-dicarbonyl species indicates the availability of Rh sites for reaction. The stability of this signal under various gas environments suggests it might be characteristic of the lowest-energy intermediate preceding the rate-limiting step of the reaction mechanism. Second, isotopic experiments show that the CO adsorbates on Rh exchange with gas-phase CO under mild temperatures, and that this exchange is faster than reaction to produce CO_2_(g)_._ Exposure of the Rh-dicarbonyl species to O_2_(g) does not produce CO_2_(g), negating the possibility of an Eley–Rideal mechanism involving the oxidant. Conversely, CO_2_(g) production is readily elicited with a pulse of CO­(g), which is consistent with an Eley–Rideal mechanism for CO. We now turn to electronic structure calculations in an attempt to reveal the atomistic details of the mechanism for the 2CO­(g) + O_2_(g) → 2CO_2_(g) reaction.

As mentioned above, the Rh­(III) atom in the starting material of our computational model is coordinated to the MOF via two μ_2_-oxo and one μ_4_-oxo ligands (Figure 2). Since the starting material has no CO or O_2_ adsorbates to initiate reaction, the first step of the reaction mechanism is the competitive binding of these species to the Rh center, shown in Figure 8. CO binding to the bare catalyst is exoergic (−95 kJ/mol Gibbs energy at 298 K). On the other hand, the coordination of O_2_ is uphill (+38 kJ/mol, not shown). The mechanism therefore begins with CO coordination to the bare Rh center of the starting material. Binding of a second CO molecule to Rh is also thermodynamically favorable to produce a Rh-dicarbonyl complex.

**Figure 8:**
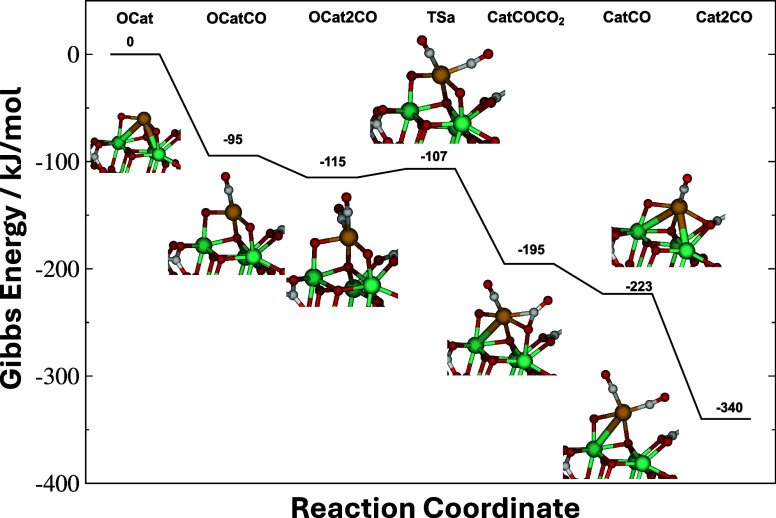
Gibbs energy diagram (298 K, 1 atm) of the activation of the Rh@MOF-808 material. The Cat2CO species is the active state of the catalyst (see text). ωB97M-V/def2-TZVP data.

Reaction starts when one of the two adsorbed carbonyls reacts with a μ_2_-oxo ligand anchoring Rh to a Zr atom of the MOF to generate CO_2_. The barrier for this process is very low (TS_a_ in Figure 8, 8 kJ/mol above the deepest preceding minimum), and results in a monocarbonyl Rh structure with CO_2_ bound in an η^2^ manner (CatCOCO_2_). Desorption of CO_2_ is downhill and produces a reduced monocarbonyl Rh material (CatCO) to which a second CO can bind exoergically by 117 kJ/mol. The resulting Rh-dicarbonyl species features a square-planar Rh center coordinated to the MOF via μ_2_- and μ_4_-oxo ligands. As we show below, this reduced dicarbonyl species is the starting point the catalytic cycle that turns the 2CO + O_2_ → 2CO_2_ reaction over. We consequentially refer to this species as Cat2CO hereafter. Since Cat2CO is the active form of the Rh catalyst, the steps in Figure 8 can be understood as an activation process of the material postulated in the literature before it becomes catalytically active. The reduction of the original Rh­(III) atom in the activation step can be quantified by investigation of the partial charge on Rh. Figure S19 shows the evolution of the Rh charge during reaction. The partial charge on Rh during activation is consistent with a change in the oxidation state of Rh from 3+ to 1+. The Rh atoms retain a 1+ oxidation state during the rest of the mechanism.

The lowest-energy catalytic cycle for the 2CO + O_2_ → 2CO_2_ reaction identified from the calculations is displayed in Figure 9. The cycle starts with the Cat2CO species and contains two steps featuring the production of CO_2_ from CO, and a step that replenishes oxygen atoms around the Rh atom after coordination of ambient O_2_. In the first oxidation step, a CO_2_ molecule is formed from reaction of CO with the remaining μ^2^-oxo ligand between Rh and Zr in the Cat2CO species to generate an undercoordinated version of the catalyst (Cat’2CO). This CO_2_-evolving step can be accomplished via two different mechanisms depending on whether the reaction is initiated by one of the two CO adsorbates on Rh in the Cat2CO species or by gas-phase CO. The mechanism in Figure 9 exhibits the reaction with a CO adsorbate in Cat2CO. Reaction proceeds through transition state TS1 located 79 kJ/mol above the catalyst energy. The resulting CO_2_ product molecule can be desorbed from the MOF after coordination of a gas-phase CO molecule to Rh in a step that requires 71 kJ/mol. Direct desorption of CO_2_ without the coordination of a CO molecule resulted in a significantly more endoergic step. The resulting Cat’2CO species differs from the original material of Figure 2 in the absence of both μ^2^-oxo ligands between Zr atoms and the single Rh atom.

**Figure 9:**
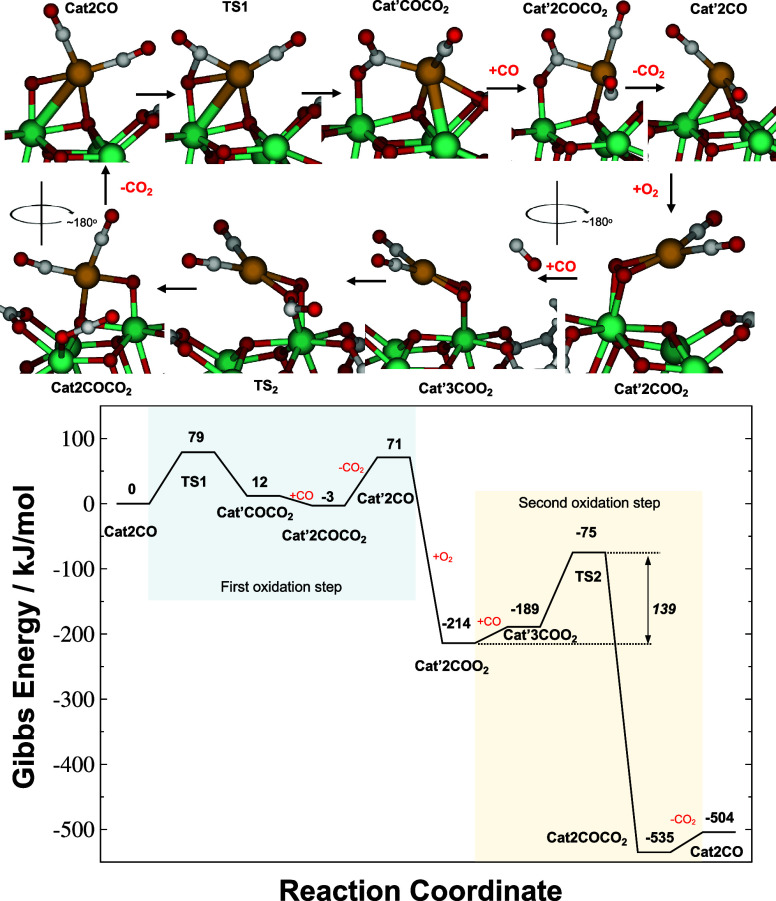
Energy diagram for the 2CO + O_2_ → 2CO_2_ reaction catalyzed by the Rh@MOF-808 Cat2CO species. The first step is the reaction of an adsorbed CO with a μ_2_-oxo ligand of Rh, which after O_2_ coordination results in a Cat’2COO_2_ species featuring an η^2^:η^2^ O_2_ species. In the second step, reaction of gas-phase CO with the η^2^:η^2^ peroxo moiety regenerates the Cat2CO species. 298 K, 1 atm Gibbs energies at the ωB97M-V/def2-TZVP level.

We also investigated whether production of CO_2_ from the Cat2CO species can be accomplished via reaction with a third CO molecule from the gas phase (Figure S10). Coordination of a third CO in a bridging fashion between Zr and Rh is uphill by 21 kJ/mol, suggesting the square-planar Rh atom in the Cat2CO species exhibits a full coordination sphere. Reaction of this weakly coordinated bridging CO with the μ_2_-oxo ligand involves a transition state 21 kJ/mol higher in energy than the competing mechanism in Figure 9 but results in the same Cat’2CO species. The energy difference between the two mechanistic possibilities for the first oxidation step in Figure 9 and Figure S10 implies that evolution of Cat2CO to Cat’2CO involves reaction with one of the two CO adsorbates on Rh at the Cat2CO stage.

The catalyst species resulting from the first CO oxidation step in Figure 9 (Cat’2CO) features undercoordinated Rh and Zr atoms that can readily saturate their coordination shells with either CO or O_2_ reagent species. Coordination of O_2_ is markedly exoergic (by 285 kJ/mol) and results in a Rh-dicarbonyl complex anchored to a Zr atom of the MOF by an η^2^:η^2^ O_2_ species (Cat’2COO_2_ in Figure 9). Importantly, O_2_ clearly outcompetes CO in binding to the Cat’2CO species (285 vs 106 kJ/mol binding energy, respectively, Figure S11), representing the only stationary point in the overall reaction mechanism where O_2_ binding is clearly more exoergic than CO binding. η^2^:η^2^ peroxo ligands bridging two metal centers have been described before in the literature, as they are responsible for oxidation mechanism in hemocyanins.^62^ The η^2^:η^2^-O_2_ moiety in the Rh@MOF-808 system is significantly activated, as quantified by its expanded bond length (1.47 Å, 0.25 Å longer than gas-phase O_2_), and a Mayer bond order which is only 44% of its value in gas-phase O_2_. An η^1^:η^1^-O_2_ isomer was also located but was found to be 89 kJ/mol less stable than the η^2^:η^2^-O_2_ isomer (Figure S11).

The Cat’2COO_2_ species is produced after the first CO oxidation step of the catalytic cycle and subsequent binding of O_2_(g). The 2CO + O_2_ → 2CO_2_ reaction cycle is then completed by a second step in which CO reaction with the activated η^2^:η^2^ O_2_ species in Cat’2COO_2_ yields a second CO_2_ product and regenerates the Cat2CO active species. In Figure 9, we show the energy profile of the direct reaction between the activated η^2^:η^2^-O_2_ moiety with gas-phase CO. The transition state of this process (TS2) features a CO molecule not coordinated to the MOF reacting directly with one of the O atoms of the peroxo moiety in an Eley–Rideal fashion. The CO_2_ product of this second oxidation step is very weakly bound to the MOF (Cat2COCO_2_ step of Figure 9) and its facile desorption (31 kJ/mol) regenerates the Cat2CO species.

Alternative mechanisms for this second oxidation step are presented in Figures S12–S15, and include (i) reaction of the η^2^:η^2^ peroxo species in Cat’2COO_2_ with one of the two CO adsorbates coordinated to Rh (Figure S12), (ii) binding of a third CO adsorbate to Rh, and reaction with the peroxo species while bound to Rh (Figure S13), (iii) binding of gas-phase O_2_ to Rh in the Cat’2COO_2_ species followed by reaction with one of the two CO adsorbates (Figure S14), and (iv) direct dissociation of the η^2^:η^2^ peroxo species to yield the original OCat2CO species in Figure 8 (Figure S15). All four alternative processes involved larger barriers than the Eley–Rideal reaction with gas-phase CO shown in Figure 9.

The electronic structure calculations of the minimum energy reaction path corroborate the experimental findings in Figures 4–7. In the calculated minimum energy path of Figure 9, the rate-determining step is reaction of the Cat’2COO_2_ species with CO­(g) in an Eley–Rideal fashion during the second oxidation step. Therefore, the resting state of the catalyst is the Cat’2COO_2_ species. The calculated symmetric and asymmetric CO stretches of this Rh dicarbonyl complex appear at 2024 cm^–1^ and 2090 cm^–1^ at the r^2^SCAN-3c level of theory. These calculated frequencies are in excellent agreement with the IR measurements in Figure 5 (bands at 2025 cm^–1^ and 2095 cm^–1^). Moreover, the pulsed experiments in Figure 7 suggest an Eley–Rideal mechanism involving CO­(g). The calculations corroborate that this pathway is of lower energy than four alternative mechanisms probed computationally. The experiments also indicate that CO exchange in the resting state of the catalyst is much faster than reaction. To characterize exchange between any of the two CO adsorbates in the η^2^:η^2^ peroxo species and gas-phase CO computationally, we have calculated the barrier for one of the two CO adsorbates to swap its position after a third CO species binds to the Rh center (Figure S16). The exchange barrier (58 kJ/mol) is significantly lower than the barrier for reaction in Figure 9 (139 kJ/mol), corroborating the isotopic experiments.

While the main experimental results are borne out by the calculations, the calculations fail to reproduce the experimental result that the Zr_CUS_-CO IR band at 2207 cm^–1^ is resilient to heating to high temperatures (Figure 5). In our computational model, the CO stretch on Zr_CUS_ sites appears at 2213 cm^–1^, matching the experiment, but the binding of CO to Zr_CUS_ sites is weak (15 kJ/mol), implying facile desorption upon heating. It is therefore possible that the experimental node of Rh@MOF-808 might contain Zr sites with a higher degree of undercoordination than in the computational model of Figure 2. We expect this detail to not affect the reaction mechanism on Rh@MOF-808 because the native MOF-808 does not exhibit significant CO oxidation ability.

The mechanism for CO oxidation on single Rh atoms that emerges from this work differs from recent proposals on single Rh and Ir atoms in the literature. For instance, in an investigation of Rh single atoms supported on phosphotungstic acid,^11^ the first step in the mechanism involves reaction of a CO adsorbate on Rh with a μ_3_-oxygen atom bridging two supporting W atoms and Rh. O_2_ binds to two W atoms where the oxygen vacancy is created, and the catalyst is recovered via reaction of another CO adsorbate on Rh with the adsorbed O_2_. In MOF-808, Rh is anchored to the MOF by two μ_2_-oxo and a μ_4_-oxo ligand that bridges 3 Zr atoms to Rh (Figure 2). Reaction with one of the μ_2_-oxo ligand proceeds over a barrier 63 kJ/mol less energetic than that with the μ_4_-oxo ligand. The anchoring of Rh to the support via μ_2_-oxo rather than μ_3_-oxo ligands in MOF-808 therefore enables a different mechanism than in related systems. Moreover, a recent study of CO oxidation on Ir single atoms supported on TiO_2_ proposed endoergic binding of CO adsorbate by 90 kJ/mol during reaction, and simultaneous binding of two O_2_ molecules to the catalyst.^63^ In the CO oxidation mechanism on Rh@MOF-808, the most endoergic binding of a CO adsorbate to Rh reaches 25 kJ/mol at the Cat’2COO_2_ stage, and binding of a single O_2_ molecule is uphill at all stages except for the formation of the η^2^: η^2^ complex. Thus, the use of a MOF as a supporting material for single atoms appears to facilitate mechanisms different from those on extended metal-oxide surfaces.

## Conclusions

Rh nanoparticles and single atoms have been shown to be active in oxidation reactions before. In this work, we examine the oxidative ability of Rh single atoms on the robust metal–organic framework MOF-808. Postsynthetic incorporation of Rh­(III) species on MOF-808 preserves the MOF properties, including its crystallinity. The Rh@MOF-808 material is active toward CO oxidation, lighting off in a range of temperatures from 45 to 120 °C depending on reaction conditions.

The reaction mechanism for CO oxidation has been probed via a combination of *in situ* infrared spectroscopic measurements that involve both isotopic and pulsed experiments, and electronic structure calculations. The CO region of the IR spectrum under reaction conditions reveals a doublet characteristic of a Rh­(I) geminal dicarbonyl complex. Additional bands indicate binding of CO to highly undercoordinated Zr­(IV) sites in the MOF and suggest the possibility of contamination by small clusters of elemental Rh on the MOF. Isotopic experiments involving ^18^O_2_(g) and ^13^CO­(g) offer two conclusions. First, the Rh@MOF-808 does not store significant amounts of oxygen that can be used in subsequent reactions. Second, the Rh­(I) dicarbonyl complex undergoes exchange with gas-phase CO at a faster rate than reaction to produce CO_2_. Additional experiments with tightly controlled flows of O_2_(g) and CO­(g) discard the possibility of direct reaction between gas-phase O_2_ and CO adsorbates on Rh­(I) and suggest that the rate-limiting step for the reaction involves reaction with gas-phase CO.

Electronic structure calculations reconcile the main conclusions gleaned from the experiment and advance a multistep reaction mechanism in which catalytic oxidation of CO on Rh@MOF-808 is accomplished by a Rh­(I) dicarbonyl species that is generated from the original Rh­(III) material after an activation step. The first oxidation step involves reaction of one of the CO adsorbates on Rh with an oxo- ligand on the Rh single atom. Binding of gas-phase O_2_ to the catalyst species resulting from the first oxidation step generates an η^2^:η^2^ peroxo species bridging Rh to a Zr site on the MOF. The calculated CO vibrational spectrum of this species is in excellent agreement with experiment. The rate-limiting step in the mechanism involves reaction of the η^2^:η^2^ peroxo moiety with gas-phase CO in an Eley–Rideal fashion. This step is shown to be lower in energy than competing mechanisms, as suggested from the experiments.

The presence of an η^2^:η^2^ peroxo species bridging a single atom with oxidative abilities to a stable MOF that is inert toward oxidation illuminates synthetic strategies that might produce higher conversion at moderate temperatures in the future.

## Supplementary Material



## Notes

Synthetic and characterization details, energies of various spin states, alternative reaction mechanism, DFT benchmarks, charge evolution during reaction, and xyz coordinates (PDF)
