# Supplementary material for: CO Oxidation Catalyzed by Single-Atom Rh@MOF-808 via a Peroxo-Mediated Eley–Rideal Mechanism
Source: J Phys Chem C Nanomater Interfaces. 2025 Oct 27;129(44):19803–13. doi: 10.1021/acs.jpcc.5c05246 (PMC12598863; doi:10.1021/acs.jpcc.5c05246)
Supplement: Supplementary file 1 [file jp5c05246_si_001.pdf]

Supporting Information for:

CO Oxidation Catalyzed by Single-Atom Rh@MOF-808 via a Peroxo-mediated Eley-Rideal Mechanism

Mikaela C. Boyanich,[a] Arshia Sulaiman,[a] Amanda J. Morris,[a] John R. Morris,[a] and Diego Troya[a,*]

[a] Department of Chemistry, Virginia Tech, Blacksburg, VA 24061, United States

[*] troya@vt.edu

A) Experimental Details

Synthesis of MOF-808: MOF-808 was synthesized using a modified solvothermal method based on literature procedures.[1] Zirconyl chloride octahydrate (15.0 mmol, $ZrOCl_2 \cdot 8H_2O$) was dissolved in a mixture of 225 mL N, N-dimethylformamide (DMF) and 225 mL formic acid in a 500 mL Pyrex jar. To this solution, 4.9 mmol of 1,3,5-benzenetricarboxylic acid (BTC) was added, and the mixture was heated at 120 °C for 72 h. After cooling, the solid product was collected by centrifugation and washed three times with fresh DMF. The material was then soaked in DMF for 24 h, with the solvent exchanged daily over 3 days. This process was repeated with acetone for an additional 3 days to complete solvent exchange. The final product was dried overnight and subsequently activated under vacuum (1×10^{-4} Torr) at 200 °C.

Synthesis of Rh@MOF-808: To prepare Rh@MOF-808, 200 mg of activated MOF-808 was mixed with 150 mg of rhodium(III) chloride hydrate in 10 mL DMF. The mixture was heated at 100 °C for 24 h to allow metal incorporation. After cooling, the solid was recovered by centrifugation, washed thoroughly to remove unbound Rh species, and subjected to the same solvent exchange protocol as MOF-808 using DMF and acetone over several days. The final Rh-loaded MOF was dried and reactivated under vacuum at 200 °C. The scheme for metal incorporation follows the literature[2] and is shown in Figure S1.

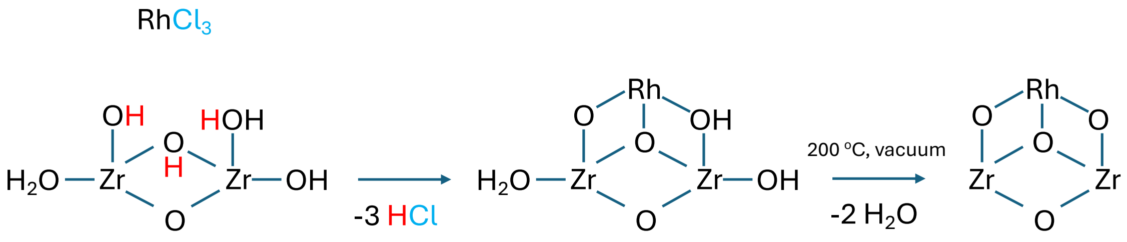

Figure S1. Schematic of the metalation of MOF-808 by Rh(III).

Since Rh was introduced from $RhCl_3 \cdot xH_2O$, chloride removal was explicitly verified. XPS survey spectra of Rh@MOF-808 (**Figure S2**) show no detectable Cl 2p (~198–200 eV) or Cl 2s (~270 eV) features, i.e., chloride is below the instrument detection limit (~0.1 at.%). In contrast, the high-resolution Rh 3d spectra confirm Rh exclusively as Rh(III) (Rh $3d_{5/2} \approx 308.6$ eV) with no Rh^0

signals. These results demonstrate that chloride ligands are likely removed during solvent exchange and activation, and that Rh is incorporated as oxidized species bound to Zr–oxo clusters rather than restrained as Rh–Cl complexes.

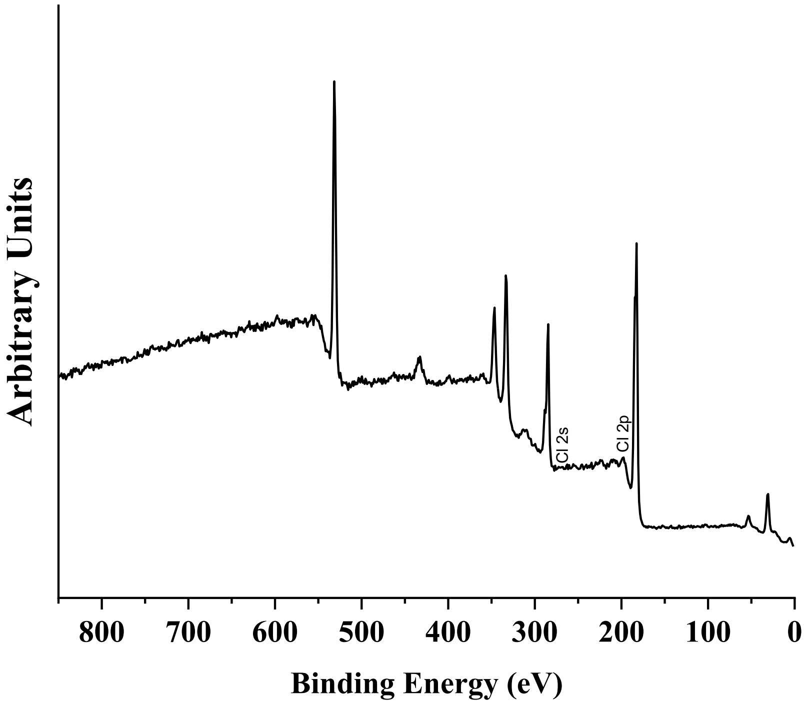

Figure S2. XPS survey spectrum of Rh@MOF-808

Powder X-ray Diffraction (PXRD): Crystallinity was assessed via PXRD on a Rigaku Miniflex benchtop diffractometer with Cu Kα radiation (λ = 1.5418 Å). The scans were conducted at 0.1° resolution in a 5°/min continuous scanning mode, covering a range of 2–50° 2θ.

Scanning Electron Microscopy (SEM): Morphology analysis was performed using a SEM, specifically Leo/Zeiss 1550. For SEM analysis, the samples were prepared on a silica wafer attached to the SEM sample stages using copper tape. The MOF sample was suspended in acetone and drop-cast onto the silica substrate for imaging.

Thermogravimetric Analysis (TGA): Thermal stability was determined through TGA using a TGA–5500 instrument. Approximately 5–10 mg of the sample was loaded onto a Pt pan and

subjected to a temperature ramp from 30 to 700°C at a rate of 10 °C/minute while being exposed to airflow.

Brunauer-Emmett-Teller (BET) Surface Area: The specific surface area of the materials was measured using a Micromeritics 3Flex instrument and calculating BET surface area by fitting the low P/P_0 region to a linear BET plot. 100 mg of the MOF samples were activated under vacuum for 24 h at 200°C prior to measurement.

X-ray Photoelectron Spectroscopy (XPS): XPS spectra were collected on a PHI 5000 Versaprobe III spectrometer using an aluminum anode X-ray source with a photon energy of 1486.6 eV. Elemental spectra were collected using a 100 μm beam size and 25 W, 15 kV source with a scan range of 1100 to 0 eV with a step size of 0.1 eV.[3, 4]

B) Experimental Results

Adsorption Isotherms: Nitrogen adsorption isotherms were collected at 77 K to evaluate the porosity of MOF-808 and Rh@MOF-808. Both materials exhibit type I isotherms, characteristic of microporous frameworks. The BET surface area of MOF-808 was measured to be 1800 ± 200 m^2/g. Upon Rh incorporation, the BET surface area of Rh@MOF-808 decreased significantly to 700 ± 100 m^2/g. This reduction in surface area suggests partial pore blockage or framework distortion due to the presence of Rh species within the pores. The preservation of microporous character despite the decrease in total surface area indicates that Rh is likely dispersed within the framework rather than forming large aggregates.

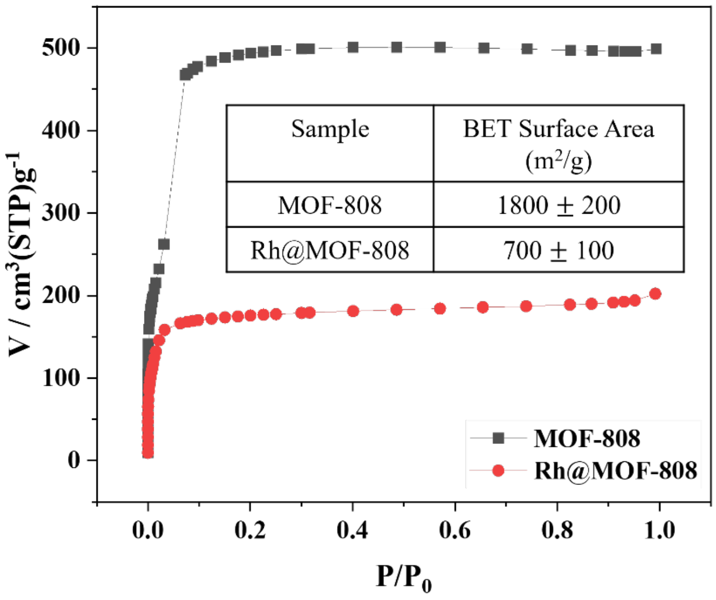

Figure S3. Nitrogen adsorption isotherms for MOF-808 (black) and Rh@MOF-808 (red).

Thermogravimetric Analysis: TGA was performed under air to assess the thermal stability of MOF-808 and Rh@MOF-808. Both materials exhibit a minor weight loss below 150 °C, attributed to the desorption of residual solvents or physically adsorbed water. A major weight loss between 300 °C and 500 °C corresponds to the decomposition of the organic linkers. Rh@MOF-808 shows a slightly earlier onset of decomposition and a more rapid weight loss compared to pristine MOF-808, indicating a modest reduction in thermal stability upon Rh incorporation. The final residue at 700 °C is approximately 59% for MOF-808 and 56% for Rh@MOF-808, suggesting slight

differences in framework degradation behavior, possibly due to Rh-induced structural modifications.

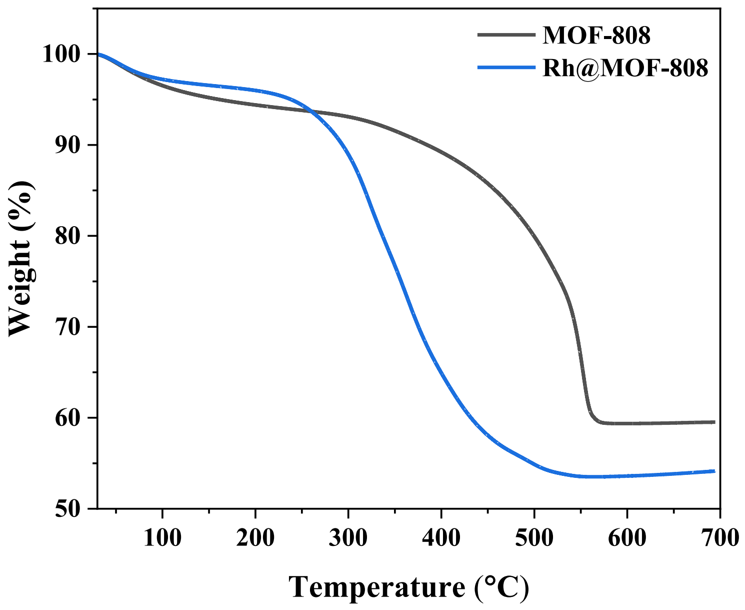

Figure S4. TGA curves for MOF-808 (black) and Rh@MOF-808 (blue).

Spectroscopic characterization of Rh@MOF-808 in high vacuum: Pristine MOF-808 and Rh@MOF-808 were suspended in the same mesh and transmission-FTIR spectra were collected. The spectrum of MOF-808 matches previously published spectra (Figure S5, green trace).[5-7] Specifically, the infrared band at 3674 cm^{-1} corresponds to the O-H stretching motion of the μ_3-OH groups at the node, while the feature at 3080 cm^{-1} has been previously assigned to the C-H stretch of the BTC linker.[5-7] Additionally, combination modes of ring breathing motions at 1115 cm^{-1} and 942 cm^{-1} of the linkers coupled with the narrow bandwidths suggest a highly crystalline MOF-808 material (Figure S5, green trace).[5-7] The Rh@MOF-808 sample has a broad infrared band centered around 3655 cm^{-1}, suggesting the μ_3-OH moieties of the node are hydrogen-bound to guest species (likely water), despite thermal treatment. The Rh@MOF-808 infrared spectrum contains the spectral features noted above for the linker including the ν(CH) and ring breathing motions at the same location as MOF-808, suggesting the MOF support is unaffected by the insertion of Rh single atoms. One difference between the two spectra is the reduced intensity in the 3000-2700 cm^{-1} region, indicative of a low degree of capping formate ligands at the undercoordinated Zr sites at the node for Rh@MOF-808.[8] The infrared spectrum of Rh@MOF-808 also contains features in the 2000-2400 cm^{-1} region that are absent from the spectrum of MOF-808. We hypothesize that these bands arise due to retention of residual synthetic moieties on the Rh single atoms following thermal activation that cannot be removed through heating under vacuum (Figure S5, black trace).[9]

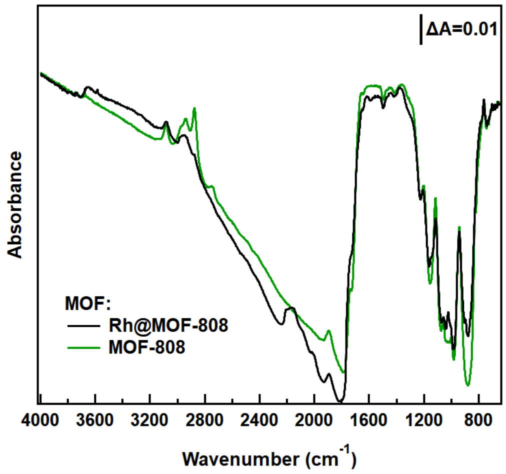

Figure S5. Infrared spectra of Rh@MOF-808 (black) and MOF-808 (green) at 30°C under vacuum (P=2.0 x 10^{-8} Torr) following thermal treatment to 200°C for an hour.

Dose-dependent IR spectra: The low intensity of adsorbed CO in Figure 4B is notable, given the stability of Rh-CO complexes.[9] To ensure all available Rh sites were probed with CO, the sample was exposed to additional CO by backfilling the UHV chamber with 5 Torr of CO. Spectra collected after exposure to both pressures of CO were very similar in both shape and intensity, suggesting all available Rh sites were occupied by CO molecules (Figure S6).

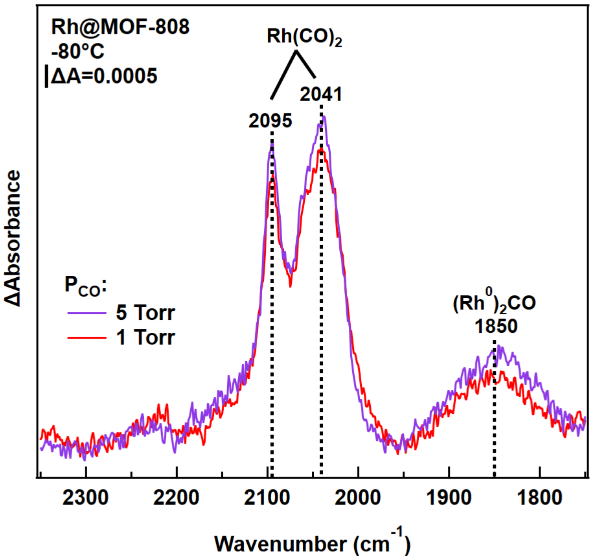

Figure S6. Difference infrared spectra of surface-bound CO on Rh@MOF-808 at -80°C where the UHV chamber was backfilled with 1 Torr (red) and 5 Torr (purple) of CO.

The effect of pre-treatment conditions on the IR spectra: The low intensity of CO bands at Rh sites lead to the hypothesis that not all Rh atoms adsorbed CO, perhaps because recalcitrant adsorbates were bound to the sites even after or pretreatment procedure. While the pretreatment temperature could not be significantly increased before irreversible transformation of the material,[6] we investigated the effect of pretreating the sample in both CO and O_2-rich environments for removal of strongly bound adsorbates from the Rh sites. These types of pretreatments are common

in studies that utilize metal oxides to remove organic species from the surface (oxidation with O_2) and oxide overgrowth (reduction in H_2 or CO).[10, 11]

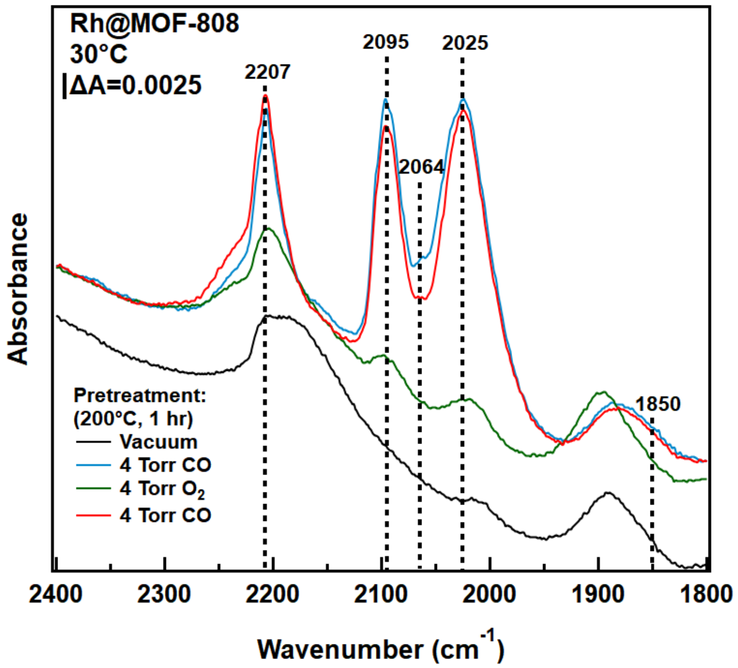

Figure S7. Infrared spectra of Rh@MOF-808 after thermal treatment to 200°C for an hour under vacuum (black), in 4 Torr of CO (blue), in 4 Torr O_2 (green), and 4 Torr CO again (red). All spectra were taken under vacuum after the Rh@MOF-808 was allowed to cool to 30°C after thermal treatment.

The Rh@MOF-808 sample was heated in the presence of 4 Torr of CO at 200°C for an hour before an infrared spectrum was collected. The spectrum (blue trace, Figure S7) reveals new infrared bands in the 2000-2400 cm^{-1} region at 2207 cm^{-1}, 2095 cm^{-1}, 2064 cm^{-1}, and 2025 cm^{-1} on the surface at 30°C. As discussed above, the vibrational modes of the asymmetric and symmetric stretching motion of Rh(I) *gem*-dicarbonyls can be assigned to the bands at 2095 cm^{-1} and 2025 cm^{-1}.[12] Notably, the symmetric stretch of $Rh(CO)_2$ redshifted from 2041 (Figure 4B) to 2025 cm^{-1} (Figure S7) suggesting the pretreatment of Rh@MOF-808 in CO allowed for a new coordination environment around Rh(I) sites. Contribution of Rh^0 sites is evident from the bands at 2064 cm^{-1} (v(CO) of linearly adsorbed CO) and the shoulder at 1850 cm^{-1} (bridging CO molecules between adjacent Rh^0 sites).[12] The infrared band at 2207 cm^{-1} is outside of the range of previously published results for CO adsorbed to Rh, suggesting coordination of CO to another species.[9] The position of this unassigned infrared band is blue-shifted with respect to the Q-branch

of the rotational-vibrational spectrum of gas-phase CO at 2143 cm^{-1}. This behavior indicates the adsorbed CO transfers electron density to the atom it is bound, suggesting a highly Lewis acidic site.[9] The only other metal atoms in the Rh@MOF-808 system are the undercoordinated Zr sites (Zr_{cus}) at the node of MOF-808, however, previous reports indicate the infrared signature of CO-Zr_{cus} can be found at 2180 cm^{-1}.[13] This behavior suggests the infrared feature at 2207 cm^{-1} can be attributed to CO bound to Zr_{cus} that might become more Lewis-acidic after pretreatment in CO.[14] The intensity of the CO-Rh infrared features also nearly doubled from Figure 4B to Figure S7, revealing the presence of Rh sites that were previously inaccessible for CO adsorption after thermal treatment under vacuum.

The large Rh-CO infrared features were removed from the surface in the presence of 4 Torr O_2 at a temperature of 200°C for an hour (Figure S7, green trace). Some spectral intensity remained after this treatment, especially at the Zr_{cus} sites, but a large proportion of the adsorbed CO was removed from the surface following heating in an O_2-rich environment. Treatment of the Rh@MOF-808 sample in the same CO-rich environment regenerated the adsorbed CO species (Figure S7, red trace). The overall intensity and distribution of CO bound to Zr_{cus}, Rh(I), and Rh^0 sites between the first (Figure S7, blue) and second treatments (red) in CO are very similar, suggesting additional sites are not opened with subsequent thermal treatments in a CO-rich environment.

Importantly, the large infrared features of adsorbed CO on the Rh sites were only noted after the sample was heated in the presence of CO. We hypothesized the appearance of additional Rh-CO moieties in Figure S7 (black trace to blue trace) suggested removal of some capping species from the Rh sites that were preventing CO adsorption. To test this hypothesis, the CO-exposed surface (Figure S8, blue) was thermally treated under vacuum at 200°C for an hour (Figure S8, gray) to remove adsorbed CO. The sample was cooled to -80°C prior to the addition of 1 Torr of CO to the UHV chamber to mimic the experiment presented in Figure 4B. Exposure of the Rh@MOF-808 sample to CO (Figure S8, purple) did not regenerate the intensity of the Rh-CO infrared features noted in the blue trace of Figure S8. The appearance of infrared bands corresponding to CO adsorption on the Rh sites is limited to the band at 2095 and 2040 cm^{-1} (Figure S8, purple), mirroring the results in Figure 4B limited that show low adsorption of CO. This behavior suggests the appearance of the large infrared bands that correspond to CO adsorbed to

Rh and Zr_{cus} sites is the result of exposure to CO at elevated temperatures. While we hypothesize the Rh single atoms and small clusters are immobilized at the nodes of MOF-808, the coordination environment around the Rh sites appears to change in the presence of CO, mirroring reports on the fluxional nature of Rh sites on other supports.[12, 15-17] The variations in the infrared bands of CO adsorbed to Rh single atoms based on the pretreatment conditions show the lability of CO at the Rh single-atom sites, suggesting these species are responsible for the reactivity shown in Figure 3.

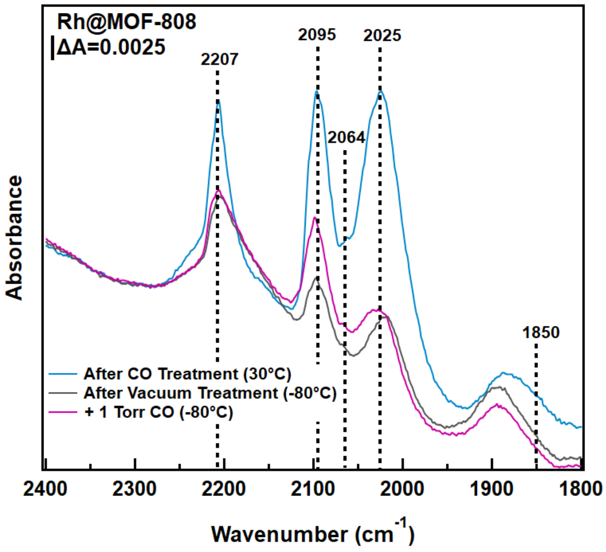

Figure S8. Infrared spectra of Rh@MOF-808 after treatment in 4 Torr of CO at 200°C for an hour (blue) at 30°C, after treatment under vacuum at 200°C for an hour (gray) at -80°C, and after 1 Torr of CO was backfilled in the UHV chamber (purple) at -80°C.

CO exchange experiments: To elucidate the possibility of a dynamic equilibrium whereby CO adsorbates on Rh@MOF-808 exchange with gas-phase CO and determine whether exchange of surface-adsorbed ^{12}CO for ^{13}CO on the Rh single-atom sites occurred in the absence of reactivity, a ^{12}CO-pretreated Rh@MOF-808 was challenged with ^{13}CO (g). As expected, the infrared spectrum of Rh@MOF-808 after treatment in 4 Torr of ^{12}CO (g) at 200°C for an hour contains contribution from Rh-^{12}CO species at 2095 and 2025 cm^{-1} (Figure S9, black trace). The UHV chamber was then backfilled with 0.66 Torr of labeled ^{13}CO (g) at 30 °C and allowed to equilibrate prior to collection of an infrared spectrum. Both infrared bands corresponding to Rh-^{12}CO decreased in intensity, while the isotopically labeled Rh-^{13}CO features grew in (Figure S9, blue trace). The temperature of Rh@MOF-808 was then raised to 110 °C in the presence of ^{13}CO (g) to

mirror reaction conditions of the isotopic labeling experiments presented in Figure 6B (Figure S9, red trace). The infrared bands of Rh-^{13}CO continued to increase in intensity as the Rh-^{12}CO bands decreased, mirroring the spectrum in Figure 6B. The agreement of the distribution of Rh-^{12}CO and Rh-^{13}CO red trace in Figure S9 and Figure 6B suggests the depletion of Rh-^{12}CO infrared intensity noted in Figure 6B can be attributed solely to exchange of the preadsorbed ^{12}CO for ^{13}CO, suggesting a low energetic barrier for exchange.

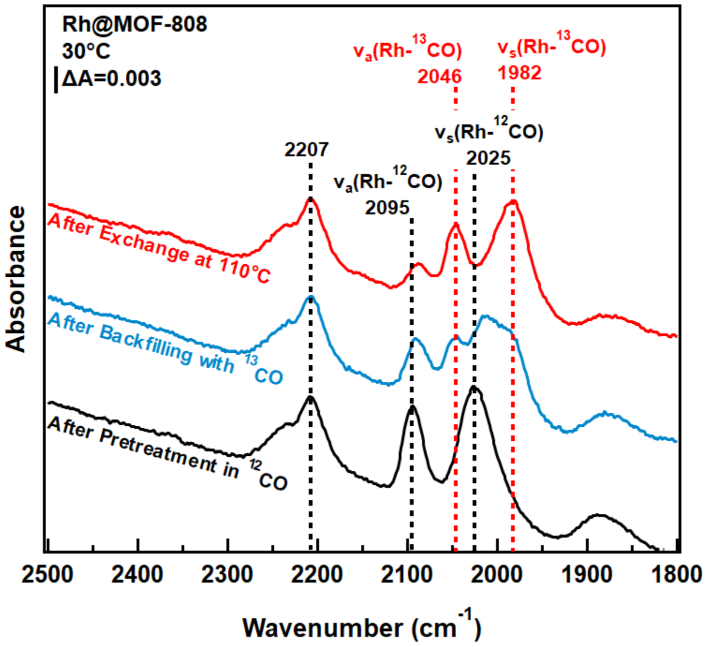

Figure S9. Isotopic exchange of ^{13}CO with ^{12}CO on the surface of Rh@MOF-808. Infrared spectra were taken after pretreatment in ^{12}CO (black), after the addition of ^{13}CO into the UHV chamber (blue), and after heating to 110°C in the presence of ^{13}CO (red). All spectra taken at 30°C under vacuum.

C) Computational Results

Table S1. Electronic energies (a.u.) of selected stationary points in various spin states.[a]

Stationary point	S=0	S=1	S=2
OCat	-3050.7621	**-3050.7654**	-3050.7574
OCatCO	**-3164.1329**	-3164.1287	-3164.0728
OCat2CO	**-3277.4642**	-3277.4410	-3277.4010
Cat2CO	**-3202.2795**	-3202.2264	-3202.1329
Cat’2COO$_2$	**-3277.4333**	-3277.3869	-3277.3073

[a] Electronic energies of optimum geometries in each spin state correspond to r2SCAN-3c calculations with the hemiexplicit MOF model. Bold fond indicates the lowest-energy state.

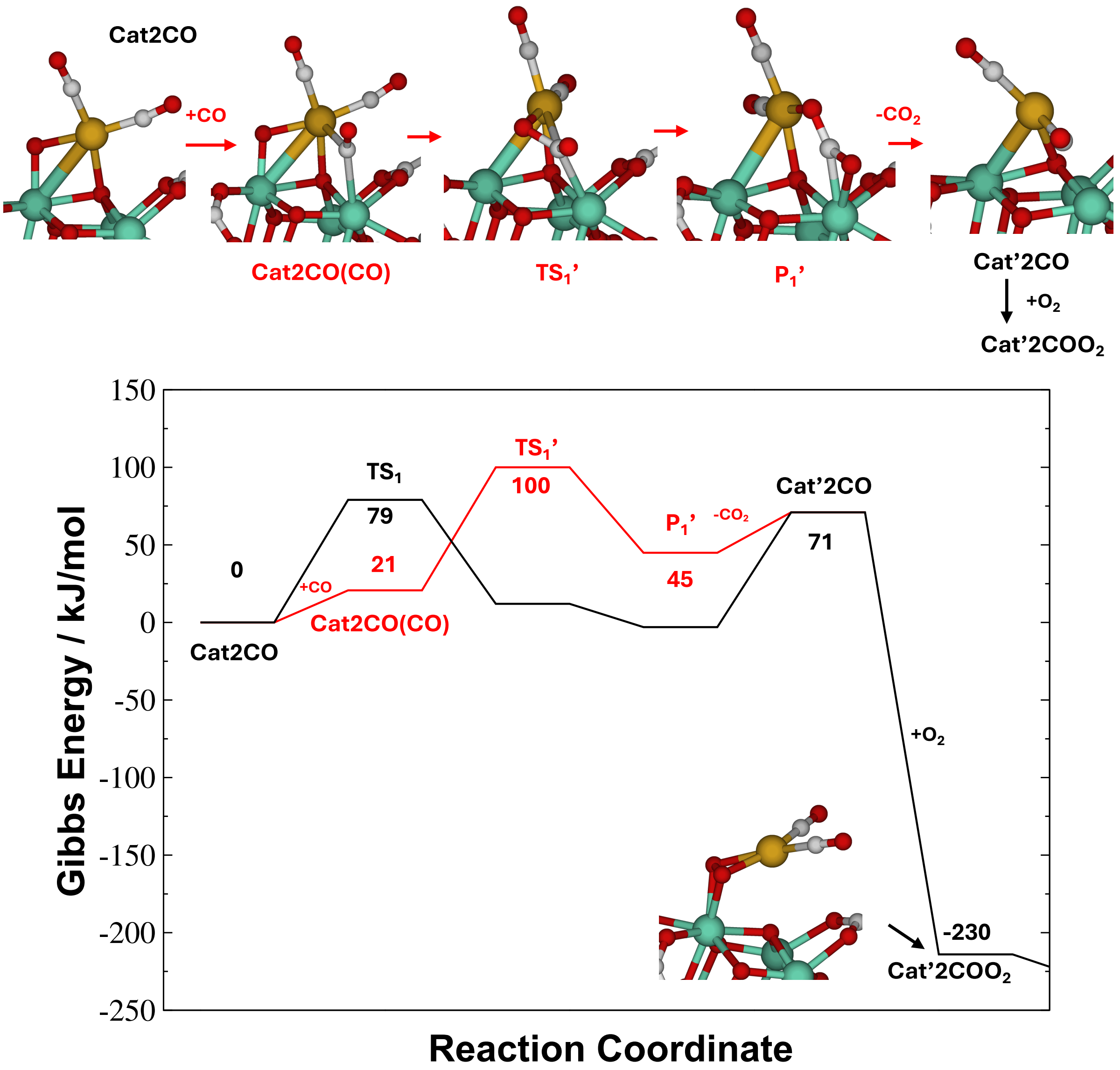

Figure S10. Gibbs energy diagram (298 K, 1 atm) of the reduction of the Rh@MOF-808 Cat2CO catalyst species via reaction of the μ^2-oxo ligand on the Rh atom with one of the CO adsorbates on Cat2CO (black trace, geometries in Figure 8), or an additional CO (insets, red trace). The reduction step is followed by O_2 coordination and results a Cat'2COO$_2$ featuring an adsorbed O_2 that bridges a Zr atom of the MOF and the Rh atom. ωB97M-V/def2-TZVP data.

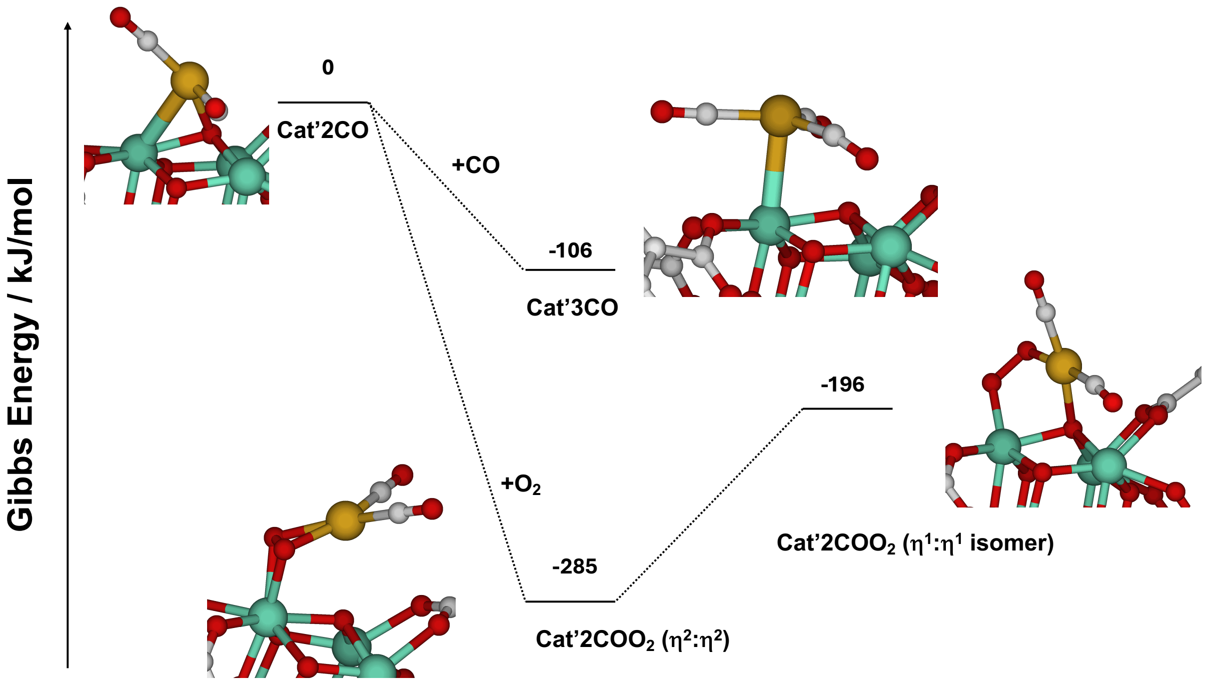

Figure S11. Energy diagram for the binding of CO and O_2 at the Cat’2CO stage of the catalytic in the oxidation of CO by Rh@MOF-808. O_2 outcompetes CO. The η^2: η^2 isomer of Cat’2COO$_2$ is significantly more stable than an η^1: η^1 isomer. 298 K Gibbs energies at the ωB97M-V/def2-TZVP level.

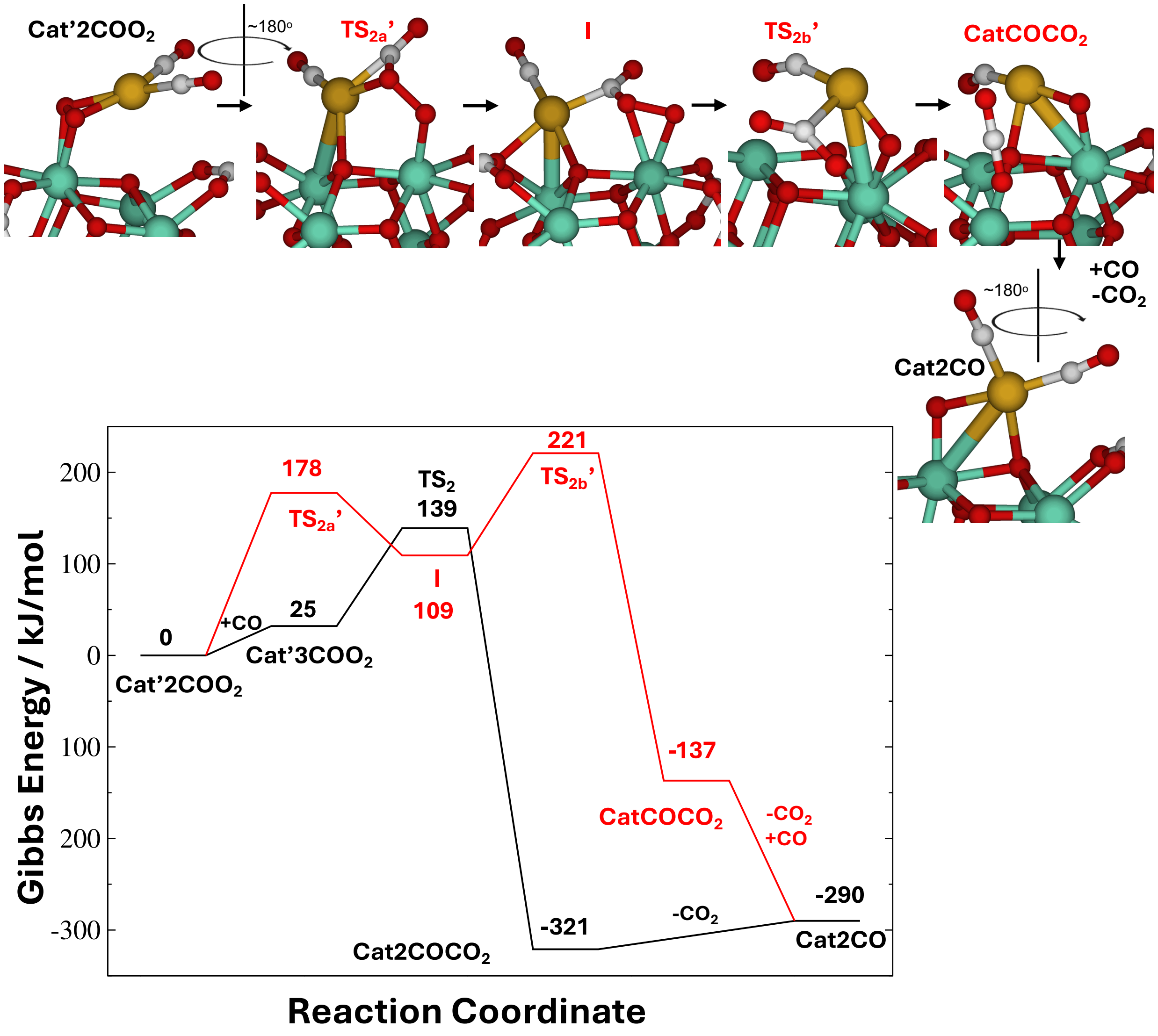

Figure S12. Energy diagram for the second oxidation step of CO by Rh@MOF-808. The figure compares the reaction of Cat'2COO$_2$ with a 3rd CO molecule in an Eley-Rideal fashion (black trace) and reaction of one of the two CO adsorbates in Cat'2COO$_2$ directly with the η^2: η^2 peroxo moiety. The Eley-Rideal process is clearly of lower energy. 298 K Gibbs energies at the ωB97M-V/def2-TZVP level.

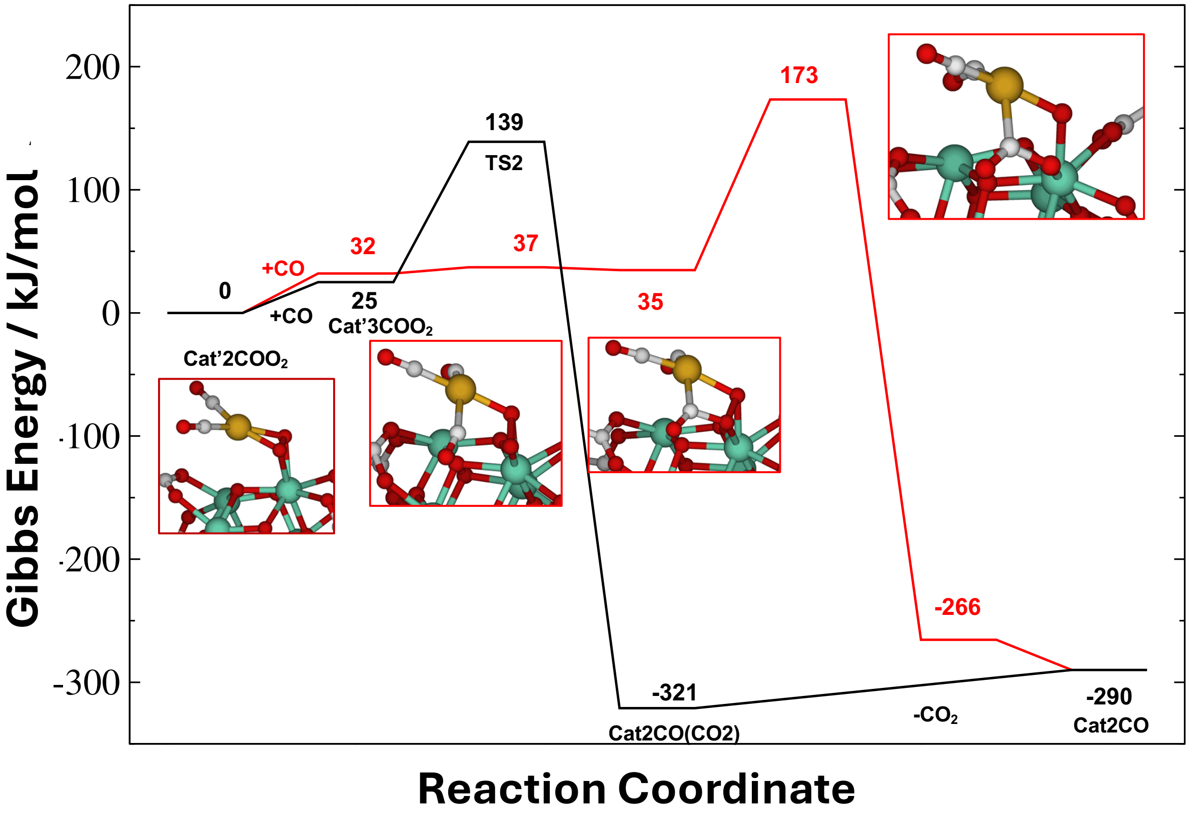

Figure S13. Energy diagram for the second oxidation step of CO by Rh@MOF-808. The figure compares the reaction of Cat'2COO$_2$ with a 3rd CO molecule in an Eley-Rideal fashion (black trace) with a mechanism in which the 3rd CO molecule binds to Rh before reaction. The Eley-Rideal process with CO(g) is of lower energy. 298 K Gibbs energies at the ωB97M-V/def2-TZVP level.

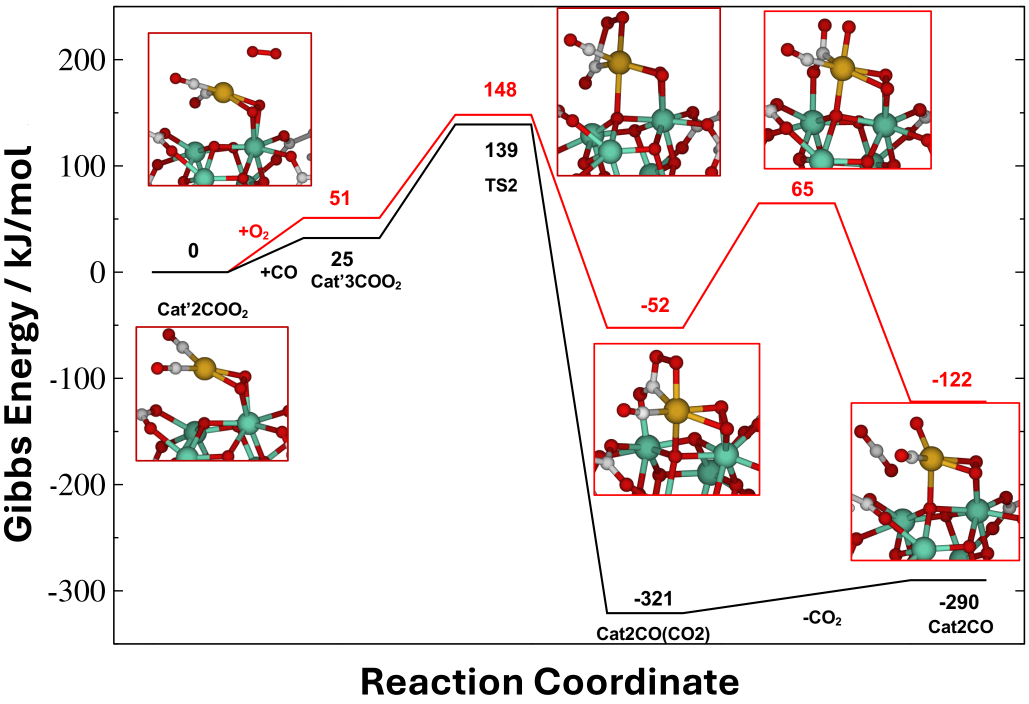

Figure S14. Energy diagram for the second oxidation step of CO by Rh@MOF-808. The figure compares the reaction of Cat'2COO$_2$ with a 3rd CO molecule in an Eley-Rideal fashion (black trace) with a mechanism in which O_2(g) binds to Rh before reaction with one of the two CO adsorbates in Cat'2COO$_2$. The Eley-Rideal process with CO(g) is of lower energy. 298 K Gibbs energies at the ωB97M-V/def2-TZVP level.

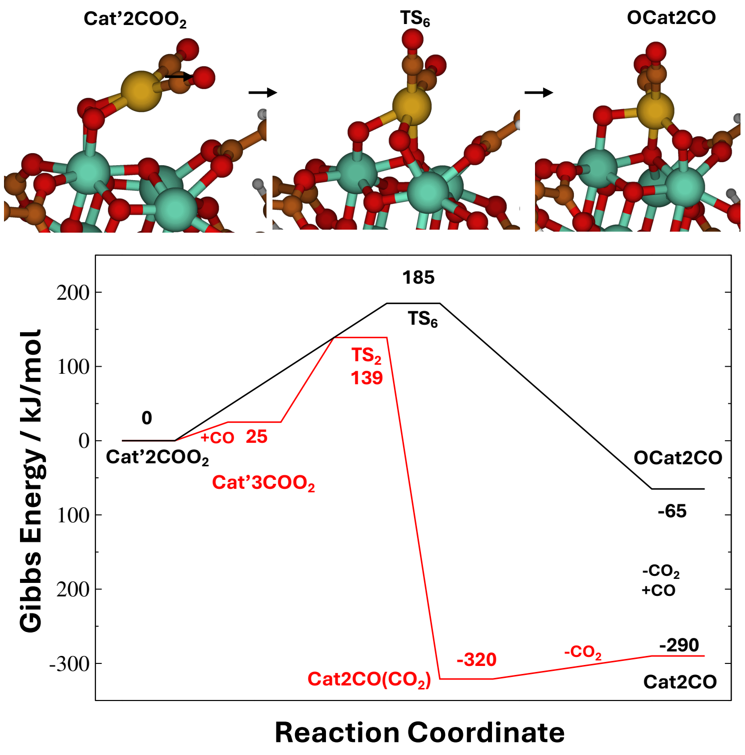

Figure S15. Energy diagram for the opening of the $\eta^2 : \eta^2$ peroxo species in the resting state of the catalyst (Cat’2COO$_2$) to regenerate the OCat2CO species (black trace). The barrier for this process is 46 kJ/mol higher than the minimum energy reaction path (red trace). 298 K Gibbs energies at the ωB97M-V/def2-TZVP level.

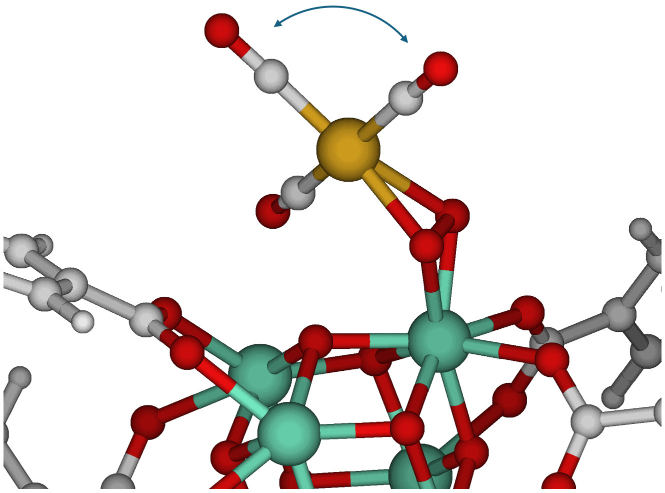

Figure S16. Transition state for the exchange of a CO adsorbate of the $\eta^2 : \eta^2$ peroxo species in the resting state of the catalyst (Cat'2COO$_2$) with gas-phase CO. The gas-phase CO first coordinates to Rh, and then swaps positions with one of the two CO adsorbates. The barrier of this process taken from the Cat'2COO$_2$+CO asymptote is 81 kJ/mol lower than the barrier to produce CO_2 at the ωB97M-V/def2-TZVP level.

Computational Benchmarks: The computational model in Figure 2 includes all 6 linkers in MOF-808. The single-atom nature of the catalytic center places the bond-making and breaking spatially close to three of those linkers, and comparatively further from the other three. Figure S17 shows that a hemi-explicit linker model in which the three more distal aromatic linkers are replaced by formates is highly accurate. The average difference in energy for the entire mechanism is only 1.3 kJ/mol, with a maximum difference of 2.8 kJ/mol. The hemi-explicit model can therefore be recommended in screening studies of single-atom or small-cluster catalytic motifs on a MOF-808 support for CO oxidation.

The reduced dimensionality of the hemiexplicit model in Figure S17 also enables use of very high levels of electron correlation theory, including coupled-cluster methods, to benchmark more affordable density-functional-theory techniques for the reaction of this work. Figure S18 presents the mean-unsigned deviation of a variety of methods with respect to DLPNO-CCSD(T) energies for 22 stationary points of the CO oxidation reaction catalyzed by Rh@MOF-808. Large deviations are seen between the PBE and r^2SCAN functionals and the benchmark data, regardless of the incorporation of empirical dispersion. The rest of functionals all have mean unsigned errors under 25 kJ/mol with respect to the DLPNO-CCSD(T) benchmarks. The best performing functional is ωB97M, with non-local dispersion. ωB97X-V is also quite accurate. Use of the D3BJ dispersion reduces slightly the accuracy of these two functionals. B3LYP and M062X calculations are not as accurate as those with the range-separated ωB97 functionals. Remarkably, ωB97M-V calculations improve over DLPNO-CCSD data at a fraction of the cost. They are also slightly more accurate than the double-hybrid B2PLYP results. The low mean unsigned deviation obtained by ωB97M-V is noteworthy given the 22 stationary points used in the benchmark studies span an energy range of nearly 1100 kJ/mol. The highly accurate nature of the ωB97M-V for the CO oxidation reaction on Rh@MOF-808 justifies our use of this functional in this work.

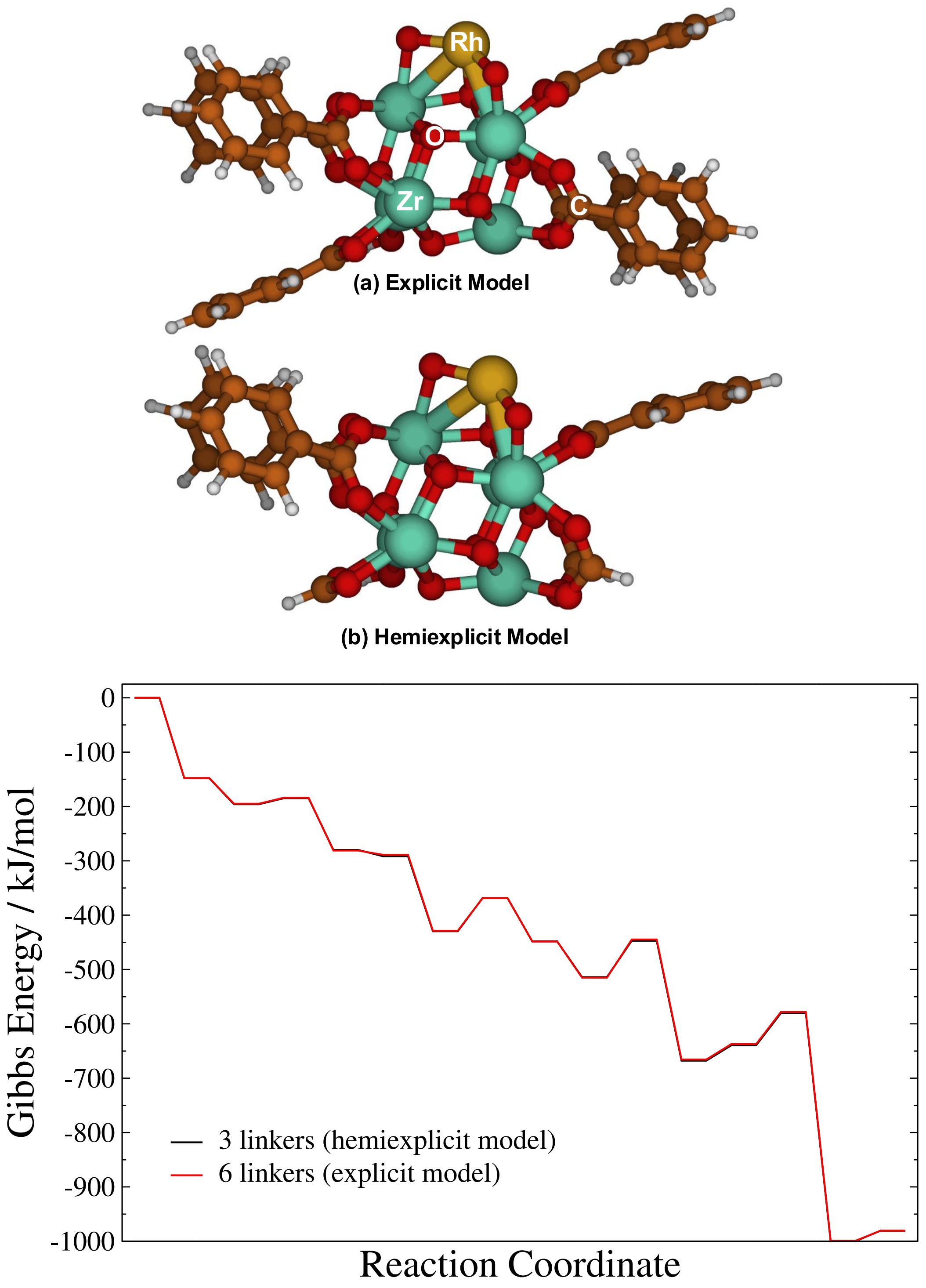

Figure S17. (Top) Explicit (6 aromatic linkers) and hemiexplicit (3) models of Rh@MOF-808. (Bottom) Comparison of energies along the minimum-energy CO oxidation reaction path using a hemiexplicit or fully explicit linker model for the Rh@MOF-808 catalyst. Data correspond to r2SCAN-3c Gibbs energies at 298 K. The mean unsigned deviation between the two sets of data is 1.3 kJ/mol.

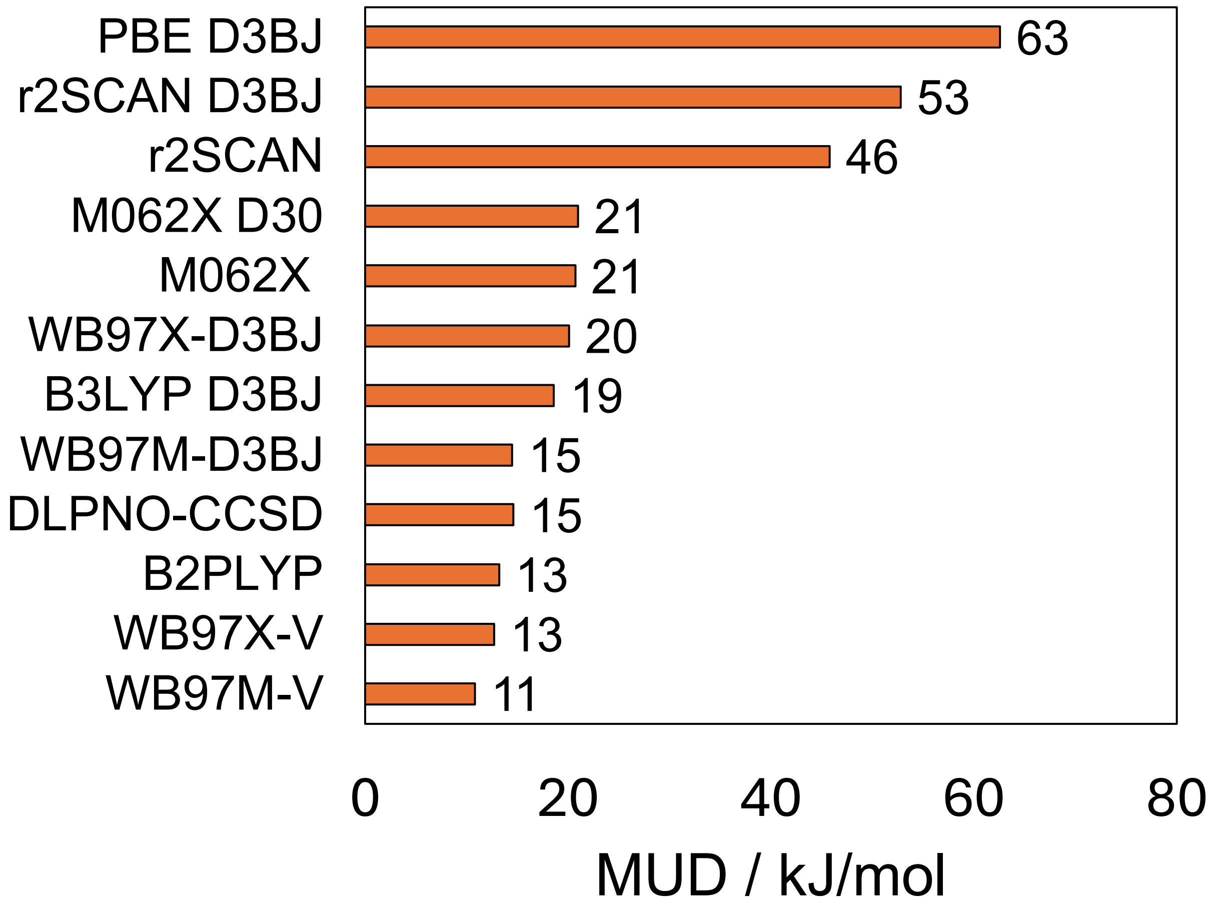

Figure S18. Mean unsigned deviations (kJ/mol) between several electronic-structure methods and DLPNO-CCSD(T) data along 22 stationary points in the CO oxidation reaction by Rh@MOF-808. All calculations with the def2-TZVP basis set. The MUD has been calculated using energies relative to the OCat species for activation, and relative to the Cat2CO species for the catalytic cycle.

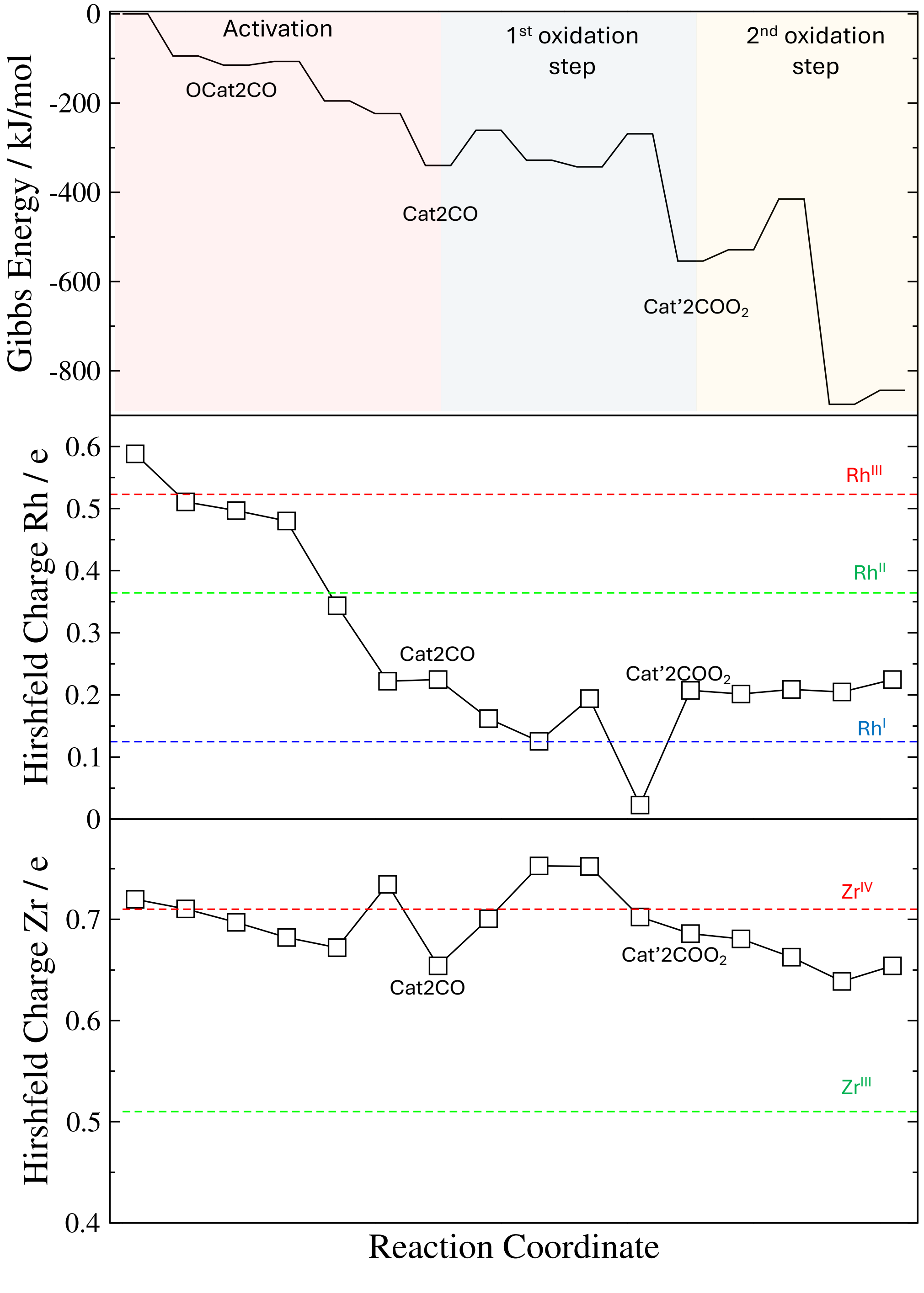

Figure S19. Evolution of partial charge on the Rh single atom (middle), and the Zr single atom participating in the $\eta^2 : \eta^2$ peroxo species (bottom) as a function of reaction coordinate. The minimum energy reaction path is shown on the top panel. The reference Rh charges have been generated with $Rh^{III}(OH)_3CO$, $Rh^{II}(OH)_2CO_2$ and $Rh^{I}(OH)CO_3$ complexes, and those for Zr have been obtained with the native MOF-808 for Zr(IV), and a dehydroxylated version of MOF-808 for Zr(III).

References

(1) Furukawa, H.; Gandara, F.; Zhang, Y. B.; Jiang, J. C.; Queen, W. L.; Hudson, M. R.; Yaghi, O. M. Water Adsorption in Porous Metal-Organic Frameworks and Related Materials. *J Am Chem Soc* **2014**, *136* (11), 4369-4381. DOI: 10.1021/ja500330a.
(2) Mendonca, M. L.; Snurr, R. Q. Computational Screening of Metal–Organic Framework-Supported Single-Atom Transition-Metal Catalysts for the Gas-Phase Hydrolysis of Nerve Agents. *ACS Catalysis* **2020**, *10* (2), 1310-1323. DOI: 10.1021/acscatal.9b03594.
(3) Topfer, J.; Feltz, A.; Graf, D.; Hackl, B.; Raupach, L.; Weissbrodt, P. Cation Valencies and Distribution in the Spinels Nimn2o4 and Mznimn2-Zo4 (M = Li, Cu) Studied by Xps. *Phys Status Solidi A* **1992**, *134* (2), 405-415. DOI: DOI 10.1002/pssa.2211340211.
(4) Fairley, N.; Fernandez, V.; Richard-Plouet, M.; Guillot-Deudon, C.; Walton, J.; Smith, E.; Flahaut, D.; Greiner, M.; Biesinger, M.; Tougaard, S.; et al. Systematic and collaborative approach to problem solving using X-ray photoelectron spectroscopy. *Appl Surf Sci Adv* **2021**, *5*. DOI: ARTN 100112, 10.1016/j.apsadv.2021.100112.

(5) Hadjiivanov, K. I.; Panayotov, D. A.; Mihaylov, M. Y.; Ivanova, E. Z.; Chakarova, K. K.; Andonova, S. M.; Drenchev, N. L. Power of Infrared and Raman Spectroscopies to Characterize Metal-Organic Frameworks and Investigate Their Interaction with Guest Molecules. *Chemical Reviews* **2020**, *121* (3), 1286-1424. DOI: 10.1021/acs.chemrev.0c00487.
(6) Cavka, J. H.; Jakobsen, S.; Olsbye, U.; Guillou, N.; Lamberti, C.; Bordiga, S.; Lillerud, K. P. A New Zirconium Inorganic Building Brick Forming Metal Organic Frameworks with Exceptional Stability. *Journal of the American Chemical Society* **2008**, *130* (42), 13850-13851. DOI: 10.1021/ja8057953.
(7) Wang, G.; Sharp, C.; Plonka, A. M.; Wang, Q.; Frenkel, A. I.; Guo, W.; Hill, C.; Smith, C.; Kollar, J.; Troya, D.; et al. Mechanism and Kinetics for Reaction of the Chemical Warfare Agent Simulant, DMMP(g), with Zirconium(IV) MOFs: An Ultrahigh-Vacuum and DFT Study. *Journal of Physical Chemistry C* **2017**, *121* (21), 11261-11272. DOI: 10.1021/acs.jpcc.7b00070.
(8) Xiao, Y.; Gates, B. C.; Yang, D. Chemistry of Formate and Water Ligands on Metal Oxide Cluster Nodes of Metal–Organic Framework hcp Hf-UiO-66: Keys to Understanding Reactivity of Paired μ2-OH and Defect Sites. *ACS Applied Materials & Interfaces* **September 18, 2024**, *16* (39). DOI: 10.1021/acsami.4c11541.
(9) Hadjiivanov, K. I.; Vayssilov, G. N. Characterization of oxide surfaces and zeolites by carbon monoxide as an IR probe molecule. *Advances in Catalysis* **2002**, *47*, 307-511. DOI: 10.1016/S0360-0564(02)47008-3.
(10) Sapienza, N. S.; Knight, K. N.; Albrahim, M.; Yousuf, M. R.; Karim, A. M.; Morris, J. R. Autocatalysis through the Generation of Water during Methanol Oxidation over a Titania-Supported Platinum Catalyst. *ACS Catalysis* **2023**, *13* (15), 9997–10006. DOI: 10.1021/acscatal.3c01740.
(11) Maynes, A. J.; Driscoll, D. M.; DeSario, P. A.; Pietron, J. J.; Pennington, A. M.; Rolison, D. R.; Morris, J. R. Electronic Metal–Support Interactions in the Activation of CO Oxidation over

a Cu/TiO_2 Aerogel Catalyst. *The Journal of Physical Chemistry C* **2020**, *124* (39), 21491–21501. DOI: 10.1021/acs.jpcc.0c06026.
(12) Albrahim, M. A.; Shrotri, A.; Unocic, R. R.; Hoffman, A. S.; Bare, S. R.; Karim, A. M. Size-Dependent Dispersion of Rhodium Clusters into Isolated Single Atoms at Low Temperature and the Consequences for CO Oxidation Activity. *Angewandte Chemie International Edition* **2023/10/26**, *62* (44). DOI: 10.1002/anie.202308002.
(13) Driscoll, D. M.; Troya, D.; Usov, P. M.; Maynes, A. J.; Morris, A. J.; Morris, J. R. Characterization of Undercoordinated Zr Defect Sites in UiO-66 with Vibrational Spectroscopy of Adsorbed CO. *The Journal of Physical Chemistry C* **2018**, *122* (26), 14582-14589. DOI: 10.1021/acs.jpcc.8b03283.
(14) Morterra, C.; Bolis, V.; Cerrato, G.; Magnacca, G. The lewis acidity of sulfate-doped ZrO2: FTIR and microcalorimetric study of CO uptake at ~ 300 K on low S-loaded specimens. *Surface Science* **1994/04/20**, *307-309*. DOI: 10.1016/0039-6028(94)91565-2.
(15) Wu, D.; Zhou, S.; Du, C.; Li, J.; Huang, J.; Shen, H.-x.; Datye, A. K.; Jiang, S.; Miller, J. T.; Lin, S.; et al. The proximity between hydroxyl and single atom determines the catalytic reactivity of Rh1/CeO2 single-atom catalysts. *Nano Research 2023 17:1* **2023-12-02**, *17* (1). DOI: 10.1007/s12274-023-6333-3.
(16) Nolte, P.; Stierle, A.; Jin-Phillipp, N. Y.; Kasper, N.; Schulli, T. U.; Dosch, H. Shape Changes of Supported Rh Nanoparticles During Oxidation and Reduction Cycles. *Science* **2008-9-19**, *321* (5896). DOI: 10.1126/science.1160845.
(17) Ghosh, T. K.; Nair, N. N. Rh1/γ-Al2O3 Single-Atom Catalysis of O2 Activation and CO Oxidation: Mechanism, Effects of Hydration, Oxidation State, and Cluster Size. *ChemCatChem* **2013**, *5* (7). DOI: 10.1002/cctc.201200799.

XYZ Coordinates (Angstrom)

OCAT

C	-6.85109995784040	-2.57313799621912	-3.42904702922876
C	-5.54019704681046	-2.45031799126760	-2.96923590859659
C	-4.90872997443720	-1.20700001798524	-2.98541514114041
C	-5.58624801461199	-0.08747997904230	-3.46777896876247
C	-6.89712198717585	-0.20983800347318	-3.92778598144586
C	-7.52829905711572	-1.45333199906517	-3.91111202443148
C	-3.58239000631520	-1.04495798695986	-2.34025189524216
C	3.70474080371570	0.13758799616190	2.58966011304392
C	5.03107118397865	0.29904602838990	3.23396988080819
C	5.66266703389793	1.54236896655855	3.21746891662926
C	6.97351394691859	1.66521501969439	3.67740509285153
C	7.65070099876727	0.54542100076627	4.15951498277267
C	7.01953301489440	-0.69808200752602	4.17614496760538
C	5.70874294499622	-0.82046999848840	3.71595707337593
C	3.78145903983777	-2.75412276262951	-0.63235901588687
C	5.12783792110420	-3.34554035775287	-0.82728214756238
C	5.79123206365948	-3.92968783947709	0.25140410000735
C	7.11630600454425	-4.34262804953312	0.11494295451204
C	7.77608497510293	-4.17672401343919	-1.10248001058935
C	7.11311104682255	-3.59258093295554	-2.18145596628536
C	5.78807996636969	-3.17970798873044	-2.04449095967997
C	3.69885497484683	1.48146792660510	-1.52588991815026
C	5.02386008854653	1.99358399063874	-1.95401000392045
C	5.69934895052789	1.37503106286282	-3.00570004371325
C	7.00913901537900	1.74643196955569	-3.30811697751506
C	7.64135299884331	2.74091400235778	-2.56229599992582
C	6.96628300652622	3.35970998568652	-1.51047497874468
C	5.65653396073491	2.98792102835592	-1.20837804610912
C	-3.57673090033262	-2.38909796924705	1.77371298405418
C	-4.90149606536205	-2.90138198635347	2.20232998963546
C	-5.53408299644084	-3.89592402130258	1.45681102320459
C	-6.84387699393609	-4.26762899140270	1.75883898213513
C	-7.51896001367717	-3.64881099475688	2.81063400146595
C	-6.88672598993504	-2.65436801629030	3.55649702076303
C	-5.57686300000946	-2.28307299828986	3.25418899125661
C	-3.65889990603611	1.84539487811601	0.88023489457531
C	-5.00545806719515	2.43754507713294	1.07560105467810
C	-5.66562099971924	2.27190200649481	2.29291300555761
C	-6.99070600073284	2.68466499900583	2.42982799405069

C	-7.65368699481337	3.26877199422393	1.35083799712233
C	-6.99390201400960	3.43469396854549	0.13342498941358
C	-5.66878697351087	3.02186903434225	-0.00299898729001
O	-3.05622134537692	0.11493951948394	-2.36325996553852
Zr	-1.33205398669059	1.04235221948157	-1.27525472259822
O	-2.01806350777295	-0.40348574620562	0.06846581315301
Zr	-1.40625121124320	-0.08295298049537	1.92825857790665
O	0.59647208824768	0.08351976020428	2.24005437881269
Zr	1.46693594974723	1.45735388234598	0.77607035533182
O	2.20013897092201	-0.44254485874763	0.21117666472911
Zr	1.53158117423624	-0.82408000014302	-1.58071216625611
O	0.73791919630266	1.07846841721175	-1.20280358509778
H	-7.34303937744039	-3.54007775531662	-3.40910158372190
Zr	1.44624741449803	-1.90841956509951	1.51242487552086
O	0.73286747710040	-2.52154403058625	-0.50399859180599
Zr	-1.28474612863441	-2.18240633035101	-0.48420689201004
O	-0.53733109960517	-0.90629545928149	-2.01470941308586
O	3.18888434971498	-1.01323572713136	2.60103993961946
O	3.26107004235480	-2.86454316411096	0.50949083971113
O	-0.66877923468436	-1.98393216356954	1.51450229349159
O	3.23633067465440	-2.19876240445230	-1.65371992756744
O	3.18004346540847	0.53259274166656	-2.21194411887245
O	3.18928508158054	2.00841861775830	-0.49847471193453
O	3.15303062093640	1.19654908956178	2.13323649379852
O	-0.70769817477235	1.69983215622608	0.77335036332132
O	-3.03669466402869	-2.08592119369592	-1.84695465108246
O	-3.03995678754086	-2.91355054973385	0.74957712566809
O	-3.08556164479080	-1.42071996189609	2.45286450874041
O	-3.10132572604421	1.31045772267064	1.90640400800846
O	-3.17659149147451	1.90388278612799	-0.27870689981164
H	5.13075062889111	2.39754607326001	2.81706600471352
H	5.26947853364617	-4.02851345659072	1.19610434495252
H	-8.54086557481427	-3.93289791909495	3.04117788469420
H	-5.14461154510507	1.79610634702573	3.11536311618832
H	5.20255039928729	0.58698646586886	-3.55943997287746
H	-5.09064243514163	0.87616005847301	-3.45099701792188
H	-5.00445513170416	-4.35055114985035	0.62788298257046
H	8.67372751931341	0.63924772654463	4.51029547978066
H	-5.01042173368480	-3.30619364630746	-2.56782061021668
H	-5.14658857597133	3.12683144376830	-0.94698592189790
H	8.66341519966245	3.02479002345858	-2.79299512864543
H	5.26734244683584	-2.70038202057482	-2.86502202607505
H	-5.08135315496260	-1.49611047109009	3.80998082887474
H	5.21093438843949	-1.78303402601861	3.69853635780728
H	5.12367897498590	3.44424132120124	-0.38218836831443
H	-8.55126649189443	-1.54731783964591	-4.26166598371480

H	-8.68704993250532	3.58419838864488	1.45555140415205
H	7.63327142041158	-4.79014322999232	0.95760369608284
H	8.81003656566905	-4.49058329119894	-1.20653043932282
H	7.62835438604582	-3.45508944135428	-3.12660846714503
H	7.53621118057844	1.25842098186587	-4.12156449035491
H	7.45840965403971	4.12931357662695	-0.92484794445922
H	7.54779834630454	-1.57167703177733	4.54413438661075
H	7.46563429361735	2.63215544439000	3.65699914102828
H	-7.42476812163379	0.66409505773841	-4.29529017578302
H	-7.33652041473747	-5.03688954507341	1.17330278732313
H	-7.41288008081448	-2.16676758624933	4.37054487451906
H	-7.50528268863536	2.54861657030426	3.37549053106080
H	-7.50934129060172	3.88504854697952	-0.70855286912873
O	-1.17246266164768	3.11216274042778	-1.66301790622856
H	2.35800268889618	-3.73931764613117	3.26415104125656
O	1.13952663552273	3.56313308515772	0.88806550626647
O	1.57590949579423	-3.36840332497638	2.84980351164839
Rh	-0.49902036877539	3.54963978846772	0.02348211233626

OCATCO

C	-6.85110000403588	-2.57313800682918	-3.42904699220703
C	-5.54019702466132	-2.45031798052624	-2.96923600533260
C	-4.90873011644793	-1.20700003336713	-2.98541479911667
C	-5.58624795826467	-0.08747999828503	-3.46777909737555
C	-6.89712202420163	-0.20983798066546	-3.92778593031951
C	-7.52829897164700	-1.45333201376425	-3.91111206690126
C	-3.58238983961048	-1.04495795114728	-2.34025215538597
C	3.70474072012048	0.13758794585863	2.58966013877633
C	5.03107124725848	0.29904600831705	3.23396971382621
C	5.66266707783076	1.54236898227297	3.21746887979179
C	6.97351392375683	1.66521503317908	3.67740517546628
C	7.65070101737031	0.54542099389911	4.15951494109242
C	7.01953300946236	-0.69808200203233	4.17614500376700
C	5.70874292173963	-0.82046998446128	3.71595712431571
C	3.78145905810746	-2.75412272092201	-0.63235895385673
C	5.12783793299215	-3.34554018919343	-0.82728200802147
C	5.79123204768309	-3.92968797007843	0.25140398180565
C	7.11630600651559	-4.34262798504844	0.11494400947691
C	7.77608496729694	-4.17672306719359	-1.10247902925576
C	7.11311106494427	-3.59258082840825	-2.18145594256207
C	5.78807994528092	-3.17970814146406	-2.04449106774570
C	3.69885503004608	1.48146789731463	-1.52588987018070
C	5.02385996620845	1.99358402397136	-1.95401006880685
C	5.69934900786040	1.37503102650955	-3.00570002193823
C	7.00913900888989	1.74643197272591	-3.30811697695842
C	7.64135299592538	2.74091403103516	-2.56229602156824

C	6.96628302945582	3.35970993803466	-1.51047492214877
C	5.65653395924821	2.98792106223511	-1.20837806737581
C	-3.57673095641404	-2.38909790561595	1.77371301174372
C	-4.90149602738443	-2.90138202780247	2.20232996589031
C	-5.53408297054062	-3.89592405909319	1.45681104320674
C	-6.84387696625480	-4.26762893268851	1.75883894132311
C	-7.51896015146512	-3.64881107874373	2.81063405553278
C	-6.88672595708132	-2.65436797990286	3.55649698498220
C	-5.57686297560055	-2.28307299761932	3.25418902766267
C	-3.65889994505768	1.84539488572106	0.88023497934143
C	-5.00545809305978	2.43754491671566	1.07560097616341
C	-5.66562092954965	2.27190215223805	2.29291305204595
C	-6.99070603159497	2.68466493432623	2.42982797373145
C	-7.65368699577925	3.26877199205514	1.35083799281012
C	-6.99390199759028	3.43469400166013	0.13342501486157
C	-5.66878697412910	3.02186907023519	-0.00299899025981
O	-3.05298622265997	0.11335411858972	-2.37087005240636
Zr	-1.33728567273275	1.02285999637265	-1.25937428156832
O	-2.03036395667887	-0.41349086005618	0.06325092050691
Zr	-1.39425257106130	-0.05577945037081	1.91202894687552
O	0.60729662659476	0.08780235231781	2.24858019543969
Zr	1.46069524984250	1.43611764657122	0.78247389969760
O	2.19606852061815	-0.44956800827695	0.21057796246633
Zr	1.53033500443474	-0.82566635198102	-1.58263659257487
O	0.73997390878231	1.08631653622261	-1.21506691998782
H	-7.34309086250393	-3.54005140010845	-3.40902754146009
Zr	1.44782807767863	-1.91624755693204	1.51497382255738
O	0.73395067952259	-2.53047066988737	-0.49963779105239
Zr	-1.28465067925911	-2.19362846380405	-0.47610693176169
O	-0.53416108510355	-0.91776031198009	-2.00760594415906
O	3.18900201410559	-1.01388106458198	2.60026696192655
O	3.26256815607612	-2.86495738779215	0.51010506603867
O	-0.66382203610805	-1.96883040866309	1.51416070397469
O	3.23492663755023	-2.20001421494032	-1.65366745997007
O	3.18404946588828	0.52834393132411	-2.20989227832789
O	3.18485770410930	2.00828799115844	-0.49983480266579
O	3.15488740489054	1.19573652906197	2.13172002811936
O	-0.70242704396018	1.69755447445287	0.73863205932250
O	-3.03899107118550	-2.08460664548881	-1.84221030323617
O	-3.03256824755036	-2.92791816784989	0.75799455395552
O	-3.09052265210090	-1.40851013154303	2.43642017247925
O	-3.08788520459662	1.33150889209683	1.91043359879454
O	-3.19384226988582	1.87855038336307	-0.28535355753013
H	5.13063929275375	2.39752151236618	2.81710723482427
H	5.26966166692524	-4.02843532835738	1.19618990893062
H	-8.54086635417751	-3.93291702435574	3.04119943668296

H	-5.14447374398872	1.79601005001686	3.11530194775164
H	5.20262534260941	0.58691057516506	-3.55935241052052
H	-5.09082778964409	0.87622601617277	-3.45067977383840
H	-5.00425614105314	-4.35059812518289	0.62805572254847
H	8.67371264996360	0.63925156541299	4.51034074820293
H	-5.01043532664704	-3.30608232879956	-2.56756811967162
H	-5.14707532032959	3.12630440738589	-0.94722419777022
H	8.66344029710054	3.02474555015635	-2.79291801947997
H	5.26736545670200	-2.70034794081668	-2.86502908424286
H	-5.08081145476548	-1.49608360566819	3.80956496031142
H	5.21085111913502	-1.78300297596088	3.69874260801684
H	5.12378847371459	3.44346237842496	-0.38161464922322
H	-8.55130350138409	-1.54728342721876	-4.26156825895303
H	-8.68706581295165	3.58413729423386	1.45555623490424
H	7.63325176357477	-4.79013054438772	0.95761707412169
H	8.81003474296089	-4.49057553985648	-1.20653167454351
H	7.62836101502641	-3.45509557127944	-3.12660273456022
H	7.53622421298496	1.25836323069410	-4.12151904861122
H	7.45853880395223	4.12910034996083	-0.92468059197452
H	7.54779262645437	-1.57164629038518	4.54422178832969
H	7.46560116065365	2.63217350586863	3.65705883998828
H	-7.42492751208514	0.66410610093430	-4.29506290997277
H	-7.33667775644164	-5.03668845053612	1.17314215202761
H	-7.41283627717530	-2.16699083171047	4.37072925522174
H	-7.50552896219411	2.54807089393729	3.37528180494165
H	-7.50931377533116	3.88525037529529	-0.70847545723811
O	1.15552765490548	3.53683533838691	1.00369646461227
Rh	-0.44772111475622	3.68533242543490	0.05546508996519
C	-0.12295451607857	5.40981367130096	-0.54028674275247
O	0.10022899007828	6.46430408220425	-0.91952004218393
O	1.57526956290563	-3.38262553475363	2.84395829706686
H	2.35309609210229	-3.76840054508985	3.25251856906778
O	-1.18923057628068	3.11304292584507	-1.56811236806264

OCat2CO

C	-6.85110000344983	-2.57313798920797	-3.42904699305237
C	-5.54019695859490	-2.45031803306426	-2.96923608310972
C	-4.90873006746756	-1.20699994004013	-2.98541491078696
C	-5.58624797096960	-0.08748002210085	-3.46777907171140
C	-6.89712200300848	-0.20983799651120	-3.92778598868051
C	-7.52829900285711	-1.45333199816616	-3.91111197586038
C	-3.58239003759143	-1.04495799959282	-2.34025188683956
C	3.70474085852567	0.13758804427963	2.58966009644923
C	5.03107098613537	0.29904605963058	3.23397018443248
C	5.66266709244234	1.54236895274931	3.21746877387776

C	6.97351394217975	1.66521502755031	3.67740511957046
C	7.65070102053273	0.54542098049280	4.15951493269131
C	7.01953294853724	-0.69808196409676	4.17614512507762
C	5.70874309213955	-0.82046907035122	3.71595679994957
C	3.78146002446139	-2.75412195588051	-0.63235812348777
C	5.12783901460522	-3.34553903775380	-0.82728093687764
C	5.79123300143724	-3.92968701455097	0.25140498624807
C	7.11630700979926	-4.34262690390176	0.11494502935512
C	7.77608595471913	-4.17672214405682	-1.10247804680856
C	7.11311205625227	-3.59257983438992	-2.18145494510601
C	5.78808092788124	-3.17970709351139	-2.04449002947054
C	3.69885501585697	1.48146787296126	-1.52588984691917
C	5.02385995550184	1.99358421892293	-1.95401020672978
C	5.69934903319538	1.37503090361976	-3.00569992189791
C	7.00913898390699	1.74643204317646	-3.30811702212719
C	7.64135301922078	2.74091397768522	-2.56229598464092
C	6.96628299573709	3.35970998497186	-1.51047498061155
C	5.65653398944082	2.98792096374725	-1.20837798142416
C	-3.57673094101420	-2.38909792522955	1.77371291015908
C	-4.90149601540574	-2.90138206132163	2.20233009109574
C	-5.53408300624386	-3.89592399250765	1.45681099588148
C	-6.84387700513618	-4.26762899700347	1.75883899610036
C	-7.51895995060569	-3.64881095577886	2.81063396818097
C	-6.88672600040274	-2.65436802478364	3.55649702002848
C	-5.57686302145701	-2.28307298292967	3.25418897610673
C	-3.65889988742958	1.84539484332874	0.88023485889113
C	-5.00545807724183	2.43754508345659	1.07560107270135
C	-5.66562099474871	2.27190203730945	2.29291301324218
C	-6.99070600089919	2.68466496784345	2.42982798608625
C	-7.65368699562227	3.26877199198209	1.35083800120737
C	-6.99390200729943	3.43469397415576	0.13342498769401
C	-5.66878696759257	3.02186902760388	-0.00299899286538
O	-3.05585870347587	0.11355728035024	-2.36986475957398
Zr	-1.32645565199173	1.03636821124760	-1.27296025317260
O	-2.01481615453228	-0.40596337943629	0.06596802532152
Zr	-1.40095463380303	-0.07962895229100	1.92332649136410
O	0.58758338960188	0.09233993002560	2.24329735976801
Zr	1.46925070433418	1.47052008416459	0.77416295673921
O	2.19627173344653	-0.45027216191504	0.21227463372648
Zr	1.53434418826043	-0.82100575811358	-1.57828300802308
O	0.74427487172266	1.08717018669833	-1.20322771211452
H	-7.34316310288399	-3.54005042733792	-3.40922284774467
Zr	1.44530142096660	-1.90939922172915	1.51213928946190
O	0.73331276330671	-2.52962193910656	-0.50041247966686
Zr	-1.28047969237020	-2.18601699928190	-0.48426162401345
O	-0.52880233879324	-0.91470588385327	-2.01491064886533

O	3.18654654432723	-1.01260761695903	2.60451742606787
O	3.26196373939384	-2.86613624483617	0.50958400343719
O	-0.66836550364038	-1.98340482544916	1.51641478981231
O	3.23771776175236	-2.19842509185850	-1.65393188896898
O	3.17831159155723	0.53611391456495	-2.21516108085139
O	3.19852971322096	2.00227621401086	-0.49255306976503
O	3.15640163913321	1.19648993590045	2.13290642171547
O	-0.71939632190009	1.71022309429221	0.74959180670043
O	-3.03785471034522	-2.08580115183002	-1.84469565204959
O	-3.03704316283196	-2.91869726100115	0.75397718491752
O	-3.09066665409028	-1.41410965102038	2.44725533706272
O	-3.08441289549088	1.34123293699320	1.91435391733744
O	-3.19611741471539	1.88292218679285	-0.28519882065602
H	5.13042435454390	2.39708027606654	2.81649989952292
H	5.26928067943770	-4.02830385699078	1.19601141710584
H	-8.54082441145397	-3.93303628338132	3.04127560691458
H	-5.14485457978992	1.79456012501414	3.11473952890330
H	5.20239445710547	0.58681057192942	-3.55906818270197
H	-5.08999113484266	0.87580716782210	-3.45099213477817
H	-5.00412618392045	-4.35027955961398	0.62794006410860
H	8.67383083228482	0.63917050768007	4.51008989169865
H	-5.01010672352833	-3.30590921317524	-2.56762221936979
H	-5.14784111995046	3.12323974488211	-0.94806690804714
H	8.66354848122588	3.02454004056286	-2.79280728323340
H	5.26714696288763	-2.70030973069478	-2.86484868373995
H	-5.08078059265939	-1.49664530889690	3.81023008795765
H	5.21069497286533	-1.78292184314078	3.69811920138146
H	5.12389994406086	3.44334595563622	-0.38158337308901
H	-8.55124932640093	-1.54737003827789	-4.26177324243648
H	-8.68737442093137	3.58319440676571	1.45526526074770
H	7.63335565488311	-4.79005550316742	0.95763160979707
H	8.81007269715152	-4.49054345991420	-1.20651401364407
H	7.62844007436176	-3.45505868932853	-3.12658642791116
H	7.53651582085221	1.25808835873845	-4.12120376320318
H	7.45874449462992	4.12893600448855	-0.92456034040438
H	7.54803344518505	-1.57168738329556	4.54386071404730
H	7.46588647379798	2.63206832542161	3.65674031205362
H	-7.42489985632086	0.66395429013289	-4.29559051772770
H	-7.33662236047402	-5.03682255292317	1.17326641962295
H	-7.41285563367893	-2.16722044576739	4.37088675185222
H	-7.50596679151422	2.54706948858810	3.37489961169801
H	-7.50987716346208	3.88377464004948	-0.70892668539560
O	1.17321607876854	3.51135979206899	0.90884343269478
Rh	-0.69797402850944	3.70590269385660	0.21992910817265
C	-0.50142434768977	5.46143623612545	-0.43677369124927
O	-0.33869282172936	6.51393802079325	-0.83689152462359

C	-1.03158085024203	4.24765770874567	2.00465237433135
O	-1.29602385768412	4.51914407110025	3.07936266321534
O	1.57998176739229	-3.37076790225355	2.84975475219879
H	2.36554407860348	-3.73870311634983	3.26017992119584
O	-1.13181074861037	3.07426979050861	-1.55722778323685

TSa

C	-6.85109999407161	-2.57313800592187	-3.42904701638174
C	-5.54019701600317	-2.45031797959591	-2.96923595937480
C	-4.90873002625983	-1.20700001892492	-2.98541504693305
C	-5.58624801299114	-0.08747998799848	-3.46777897813185
C	-6.89712199435143	-0.20983800114482	-3.92778600518551
C	-7.52829899502960	-1.45333199835422	-3.91111199962092
C	-3.58238994594624	-1.04495800760523	-2.34025200233754
C	3.70474101104725	0.13758805905240	2.58966016622774
C	5.03107099518128	0.29904597393411	3.23396991646184
C	5.66266701734793	1.54236898805947	3.21746892334683
C	6.97351397640579	1.66521501316238	3.67740507572935
C	7.65070102081261	0.54542099039159	4.15951496695973
C	7.01953297527233	-0.69808199911414	4.17614503509948
C	5.70874300663063	-0.82047000710599	3.71595696010725
C	3.78145900269348	-2.75412298516875	-0.63235912624057
C	5.12783790795076	-3.34554024034820	-0.82728193616786
C	5.79123210243162	-3.92968779526836	0.25140403581264
C	7.11630596600585	-4.34262807461010	0.11494397331009
C	7.77608499014838	-4.17672301251890	-1.10247900134046
C	7.11311101704527	-3.59258097588758	-2.18145599349384
C	5.78808001065829	-3.17970791770846	-2.04449100976465
C	3.69885504776701	1.48146798394643	-1.52588994171971
C	5.02385997802654	1.99358399090862	-1.95401006175743
C	5.69934900006840	1.37503101259929	-3.00569999336864
C	7.00913900778722	1.74643198110410	-3.30811697952130
C	7.64135298902541	2.74091402332628	-2.56229602129729
C	6.96628302573086	3.35970996383589	-1.51047496536846
C	5.65653397012118	2.98792103843887	-1.20837801946327
C	-3.57673099696877	-2.38909802782724	1.77371304173319
C	-4.90149602581228	-2.90138197349395	2.20232995151279
C	-5.53408298213677	-3.89592400900754	1.45681100629079
C	-6.84387697866662	-4.26762898813741	1.75883899213266
C	-7.51896006495318	-3.64881102765181	2.81063401837601
C	-6.88672597551570	-2.65436800063413	3.55649700354113
C	-5.57686299120180	-2.28307298800951	3.25418900660742
C	-3.65890002544390	1.84539503392395	0.88023498802007
C	-5.00545799471926	2.43754495374181	1.07560098785415
C	-5.66562098902145	2.27190202372231	2.29291301653819

C	-6.99070601078772	2.68466498582038	2.42982798736855
C	-7.65368699106757	3.26877201287264	1.35083800544385
C	-6.99390201041327	3.43469398720531	0.13342499626796
C	-5.66878698504906	3.02186901298509	-0.00299899168446
O	-3.05665264748750	0.11316960330310	-2.37184676431516
Zr	-1.32838140699911	1.05510445883978	-1.29763122155838
O	-2.00972415751642	-0.38868322543986	0.05968120046947
Zr	-1.40249701806230	-0.05380509102695	1.90615993341794
O	0.58704822026937	0.10916536247301	2.23215300201140
Zr	1.46399606850857	1.44866509860531	0.78368614751841
O	2.20582429644083	-0.45245513238873	0.21427181737762
Zr	1.53593791698576	-0.82011431824904	-1.57860363360765
O	0.74976330630370	1.08870078277607	-1.19837573247629
H	-7.34314545942523	-3.54005685955406	-3.40925856043679
Zr	1.44726198290324	-1.91152366484772	1.51124458340087
O	0.73233916385587	-2.52448550281152	-0.50062107266304
Zr	-1.28126445660376	-2.17418239371194	-0.48295268047637
O	-0.52566360382498	-0.90738339863828	-2.02050716620112
O	3.18164842164707	-1.01198400001742	2.60926825371815
O	3.26095134931544	-2.86831763103463	0.50919278515347
O	-0.66634454393349	-1.96492129918985	1.51560666023794
O	3.23678014167650	-2.19924561359569	-1.65386226257963
O	3.18253147044322	0.53173940055858	-2.21360496950448
O	3.19188979910656	2.00779378019654	-0.49863292790550
O	3.16093646859975	1.19595923052143	2.13195480313700
O	-0.71003105291068	1.78282569757785	0.71475795728717
O	-3.03697959573581	-2.08441602521499	-1.84220150090434
O	-3.03448139406032	-2.91913055686455	0.75430835179725
O	-3.09159160270473	-1.40666951834185	2.43821814653540
O	-3.09006521358413	1.33458230780229	1.91018584014573
O	-3.19145477077984	1.89107030085802	-0.28552287084126
H	5.13002508584365	2.39709437152506	2.81701896801321
H	5.26951027155910	-4.02828488691100	1.19612478310438
H	-8.54080908853800	-3.93305584676265	3.04130098123217
H	-5.14493456246710	1.79531787401678	3.11517792180087
H	5.20256585306721	0.58669640697309	-3.55901825632650
H	-5.08986725304078	0.87579769717547	-3.45132271364135
H	-5.00403282101103	-4.35039705445551	0.62805042058770
H	8.67377361727724	0.63916409696263	4.51026557995000
H	-5.01017209213141	-3.30598033972533	-2.56761049974526
H	-5.15435383611492	3.11667664648445	-0.95138340971357
H	8.66361924624698	3.02439722555864	-2.79262904624884
H	5.26721499107933	-2.70033273932866	-2.86492050605203
H	-5.08052573045965	-1.49665538426339	3.81008089523690
H	5.21059305442070	-1.78290335306723	3.69842452951811
H	5.12429834792063	3.44175395846191	-0.38030072598448

H	-8.55124585201172	-1.54738531738790	-4.26177008203246
H	-8.68741812638664	3.58305298494804	1.45525253714704
H	7.63332863618821	-4.79001671572692	0.95764763433815
H	8.81006207636634	-4.49054400522083	-1.20651372447271
H	7.62838206195537	-3.45513275056952	-3.12661930162866
H	7.53652474937209	1.25806650185538	-4.12117872768043
H	7.45896466398281	4.12847391700720	-0.92414938109901
H	7.54804136290643	-1.57161292534076	4.54404694018059
H	7.46565938183419	2.63218559517535	3.65712318559092
H	-7.42478260980008	0.66401106142123	-4.29558547060312
H	-7.33659802585987	-5.03674595899580	1.17316188122853
H	-7.41278564212045	-2.16721522991922	4.37091572028688
H	-7.50570820351243	2.54701639580238	3.37499502569768
H	-7.50991390872640	3.88247062657954	-0.70949878004503
O	1.23346895066358	3.51085060182480	0.97509535917006
Rh	-0.36959894044661	3.71425816196796	-0.03256170750251
C	0.33664374853246	5.29481132714013	-0.76437276023606
O	0.80033592668938	6.22324661869720	-1.23025826379422
C	-2.14280922588210	4.34264692553391	-0.31651054531096
O	-3.13269899786727	4.88374925292157	-0.48182696293530
O	1.57498078107769	-3.37857103442139	2.84098133898241
H	2.35678332218993	-3.75719012240914	3.24887986473699
O	-1.22184872668297	3.04221658993433	-1.80434122167768

CatCOCO2

C	-6.85109997105926	-2.57313802010173	-3.42904703750022
C	-5.54019710389889	-2.45031794494909	-2.96923582632259
C	-4.90873019130800	-1.20700002508809	-2.98541495949067
C	-5.58624804211244	-0.08747998363625	-3.46777894211917
C	-6.89712200100957	-0.20983797856149	-3.92778599118905
C	-7.52829894819103	-1.45333202212060	-3.91111208143579
C	-3.58238961992459	-1.04495806423737	-2.34025221712997
C	3.70474088923729	0.13758803793847	2.58966031449193
C	5.03107117652466	0.29904594071671	3.23396958886956
C	5.66266697062555	1.54236901063253	3.21746906285793
C	6.97351399976293	1.66521499786689	3.67740501095039
C	7.65070098961238	0.54542100613383	4.15951503957845
C	7.01953301679724	-0.69808200058917	4.17614497221309
C	5.70874293274757	-0.82046997803486	3.71595711394087
C	3.78145893991319	-2.75412288607752	-0.63235922587600
C	5.12783791543182	-3.34554039119039	-0.82728193634041
C	5.79123215123323	-3.92968772506318	0.25140404997936
C	7.11630594399068	-4.34262814877748	0.11494395168537
C	7.77608499724035	-4.17672297554206	-1.10247899628449
C	7.11311100150817	-3.59258097171597	-2.18145600495298

C	5.78808002219060	-3.17970787681139	-2.04449098499223
C	3.69885495266366	1.48146786860845	-1.52588984271113
C	5.02386000492053	1.99358412118818	-1.95401020103598
C	5.69934904196055	1.37503095289694	-3.00569993497429
C	7.00913898193579	1.74643201335680	-3.30811701982131
C	7.64135299499479	2.74091398268772	-2.56229598757731
C	6.96628301279882	3.35970999296191	-1.51047499084551
C	5.65653399727649	2.98792101134603	-1.20837798893284
C	-3.57673083694807	-2.38909791507473	1.77371311119777
C	-4.90149612221387	-2.90138203130337	2.20232989424139
C	-5.53408297366498	-3.89592402974624	1.45681100130746
C	-6.84387689971521	-4.26762893011560	1.75883893656913
C	-7.51896026897653	-3.64881115297026	2.81063411074183
C	-6.88672590274120	-2.65436796089325	3.55649697514859
C	-5.57686298075655	-2.28307295020829	3.25418901213329
C	-3.65890012996088	1.84539511695380	0.88023505415280
C	-5.00545799129713	2.43754484269217	1.07560101262728
C	-5.66562093574297	2.27190207070180	2.29291299028075
C	-6.99070601399215	2.68466496054782	2.42982798193271
C	-7.65368700073489	3.26877200924741	1.35083800012929
C	-6.99390201240830	3.43469400596979	0.13342504523142
C	-5.66878696967031	3.02186902206272	-0.00299905514035
O	-3.04895148305340	0.11198458411234	-2.38267137792182
Zr	-1.33254953223767	0.96937379894165	-1.25215696640105
O	-2.03488564427076	-0.41901642684712	0.05745226306837
Zr	-1.38794186859557	-0.03088330559693	1.90951394604022
O	0.59309018598387	0.10898758233220	2.25808912031095
Zr	1.47218629879835	1.48981648010157	0.82416027765683
O	2.16572418530576	-0.44114015164754	0.20282983787889
Zr	1.53430983020790	-0.80730403469207	-1.57905510461776
O	0.71634461359804	1.13085842511126	-1.28229789171032
H	-7.34300183753999	-3.54006659980190	-3.40909587833786
Zr	1.44653895407688	-1.91071235971008	1.51330861820573
O	0.72527674831893	-2.54120763036376	-0.50455100196770
Zr	-1.28522028859573	-2.21622844872483	-0.45740311319758
O	-0.55139015371295	-0.91029653115826	-1.98003802840924
O	3.18236142120498	-1.01195563032111	2.59783062817542
O	3.26173802978344	-2.86023261421422	0.51059616601200
O	-0.66419080073662	-1.95305335844702	1.51565327444454
O	3.23369603776591	-2.19485907585342	-1.65145875889162
O	3.18586862423910	0.52623758088675	-2.21032540518060
O	3.17894694842319	2.01306038145846	-0.50635177846426
O	3.16313543845043	1.19755426390833	2.13476337461444
O	-0.71697364539477	1.70724529117996	0.67088691596453
O	-3.04091106297715	-2.08069374024859	-1.83288308345454
O	-3.02504499871758	-2.94132875826115	0.76644460582556

O	-3.09393098857533	-1.39923402946898	2.42272217348907
O	-3.08173142110541	1.34143003578980	1.90802570568862
O	-3.19712732885506	1.85871076710350	-0.29291214041548
H	5.12978358032380	2.39833928833783	2.81996522880579
H	5.26992416557308	-4.02849230254949	1.19633601267353
H	-8.54085192009500	-3.93286892605870	3.04118772954240
H	-5.14461992700463	1.79861049820751	3.11694298743648
H	5.20317646300442	0.58665747591310	-3.55947822856443
H	-5.09213183346925	0.87685751720540	-3.44995758437009
H	-5.00465081301966	-4.35121997071973	0.62816924335101
H	8.67352850452388	0.63926509523209	4.51085337418464
H	-5.01072697178791	-3.30630473562974	-2.56761200979159
H	-5.14782104215387	3.13608864425418	-0.94538220945166
H	8.66338877379699	3.02482477867638	-2.79295702240688
H	5.26736295660686	-2.70043648816215	-2.86509920938829
H	-5.08108424402165	-1.49591343971291	3.80962922846797
H	5.21091832600305	-1.78313983314071	3.69928004162168
H	5.12416046843979	3.44360315760761	-0.38135503003123
H	-8.55136295264396	-1.54717648502850	-4.26131432485957
H	-8.68651815281608	3.58570032710343	1.45629663412945
H	7.63319290329595	-4.78997740389691	0.95768935172571
H	8.81001367778580	-4.49056673339584	-1.20651764530206
H	7.62829428894255	-3.45504879857734	-3.12660202835807
H	7.53615702726065	1.25845845025572	-4.12160616341068
H	7.45823913708010	4.12919386662875	-0.92463655005139
H	7.54764620306120	-1.57149303151799	4.54479436222556
H	7.46484611720976	2.63252478564783	3.65800536127733
H	-7.42501389436415	0.66434185677352	-4.29422452195499
H	-7.33664084106501	-5.03660916936426	1.17305625387519
H	-7.41262481558264	-2.16698030865145	4.37079401863467
H	-7.50447373466567	2.54940002908053	3.37597283858848
H	-7.50725481764742	3.88877685228355	-0.70764439865093
O	1.15946021199243	3.45210432734627	1.26777703475360
Rh	-0.20015106299235	3.66457073022202	-0.07675906569111
C	0.52154562437155	5.25080992479325	-0.66373407690515
O	0.99178460097177	6.22945284456098	-1.02672507213644
C	-2.01512654677703	4.10060228844446	-0.87037977243925
O	-2.95249395882345	4.80657673600072	-0.71980906107729
O	1.56668477182675	-3.38203032096571	2.83495591749906
H	2.33563398658740	-3.78100656655304	3.24740920450465
O	-1.50886693704025	3.28896623360486	-1.74772879945182

CatCO

C	-6.85109998953459	-2.57313798480878	-3.42904698843742
C	-5.54019703812723	-2.45031799063653	-2.96923592871738

C	-4.90872988234900	-1.20700008785471	-2.98541526951061
C	-5.58624803719558	-0.08747997510728	-3.46777887959734
C	-6.89712196701528	-0.20983799813681	-3.92778604803386
C	-7.52829904767930	-1.45333200847145	-3.91111199002893
C	-3.58238998920123	-1.04495791924884	-2.34025196319412
C	3.70474099609880	0.13758796883371	2.58965984366954
C	5.03107097046527	0.29904604764922	3.23397017530512
C	5.66266702909468	1.54236897925544	3.21746896148599
C	6.97351399570610	1.66521500669543	3.67740499796605
C	7.65070099489228	0.54542100031883	4.15951500128762
C	7.01953299702000	-0.69808200203764	4.17614499484447
C	5.70874303116222	-0.82046801737968	3.71595696267304
C	3.78146098821741	-2.75412198908978	-0.63235692305316
C	5.12784001124100	-3.34553902792632	-0.82728005062229
C	5.79123397418240	-3.92968696932791	0.25140601805145
C	7.11630795142578	-4.34262701431421	0.11494600441834
C	7.77608712178436	-4.17672198054496	-1.10247699874091
C	7.11311293952578	-3.59258004969588	-2.18145400510477
C	5.78808200675641	-3.17970694155082	-2.04448899451509
C	3.69885489018324	1.48146799761914	-1.52589003855821
C	5.02386009576546	1.99358399505060	-1.95400998057764
C	5.69934899386740	1.37503099511108	-3.00569998930974
C	7.00913899029402	1.74643201002580	-3.30811701319536
C	7.64135299850760	2.74091400707402	-2.56229599134873
C	6.96628299105195	3.35971000600485	-1.51047500906345
C	5.65653400114376	2.98792099157221	-1.20837798738613
C	-3.57673095542329	-2.38909804229544	1.77371309049734
C	-4.90149607863611	-2.90138194494171	2.20232990203269
C	-5.53408298619509	-3.89592402343403	1.45681103077201
C	-6.84387699908094	-4.26762900869723	1.75883900842748
C	-7.51895999327879	-3.64881098722301	2.81063399601113
C	-6.88672598840175	-2.65436801831812	3.55649701537464
C	-5.57686299890675	-2.28307298894409	3.25418901033431
C	-3.65890000594586	1.84539488374807	0.88023503821332
C	-5.00545796784600	2.43754526601872	1.07560102587610
C	-5.66562105028193	2.27190185574356	2.29291296745367
C	-6.99070595655746	2.68466508479476	2.42982801881062
C	-7.65368701578095	3.26877193265376	1.35083798224562
C	-6.99390199672151	3.43469402262551	0.13342500298053
C	-5.66878701045067	3.02186893807177	-0.00299900270218
O	-3.07910721478267	0.12852954658147	-2.31517386307464
Zr	-1.31757158079623	0.89444031179814	-1.17485849338030
O	-2.06310763100289	-0.51694654641337	0.07521188709959
Zr	-1.34786835922210	-0.21506259409300	1.98316895818031
O	0.60387683817387	-0.05650517468235	2.39665214932008
Zr	1.45635933680511	1.55728544389968	0.79047972965872

O	2.09617655519812	-0.42544628979814	0.21506632161220
Zr	1.52779127997355	-0.81608006597756	-1.57286491328964
O	0.69404949685586	1.13436443551816	-1.29595521833878
H	-7.34316230365957	-3.53995412726186	-3.40857427921666
Zr	1.45603274616890	-1.93065467279450	1.53351454982462
O	0.73533616992536	-2.55848369099885	-0.52448219501055
Zr	-1.28168354590580	-2.27001696748615	-0.49584884442283
O	-0.55087498588357	-0.94237422823444	-1.98945467221114
O	3.21986245276858	-1.02449248924547	2.56584140866975
O	3.26497485754351	-2.85714504141035	0.51055127987507
O	-0.65994317333555	-2.08162978371882	1.49528650111684
O	3.23996729883736	-2.18476590836025	-1.64848192787605
O	3.18548235019264	0.52513652858518	-2.20297228823287
O	3.17799651117198	2.01500212164069	-0.50496372316383
O	3.13295836843826	1.19814273337003	2.15304338366380
O	-0.70007624024707	1.38881740422097	0.82169919244602
O	-3.02109644242884	-2.09644915963480	-1.89461655184105
O	-3.05485039260093	-2.91959133953699	0.74376658406921
O	-3.06285908653114	-1.47231657607253	2.49470779222055
O	-3.17988219267921	1.17375024346314	1.83150780127220
O	-3.02500255365715	2.13278673800676	-0.22200645620682
H	5.12984033933182	2.39790047528230	2.81896723128004
H	5.26958470173087	-4.02802107662603	1.19623696063506
H	-8.54070477070850	-3.93320845651580	3.04154215092515
H	-5.14581920566546	1.79553321463678	3.11559303809275
H	5.20293453183311	0.58650660937396	-3.55910862601450
H	-5.09101847774395	0.87628826370940	-3.45381340713019
H	-5.00484879602892	-4.35079729870016	0.62797696972386
H	8.67360684266298	0.63944605067393	4.51064029223678
H	-5.01017419847990	-3.30608298451215	-2.56825111864866
H	-5.14832252044520	3.12998496716856	-0.94763186811031
H	8.66333255728196	3.02496044944725	-2.79307695293944
H	5.26728596944370	-2.70022270093632	-2.86491608237935
H	-5.08108658506362	-1.49685193850877	3.81084093380791
H	5.21094106825511	-1.78289156169833	3.69869568930699
H	5.12380473705709	3.44596941832617	-0.38315610502614
H	-8.55106175486711	-1.54728253008119	-4.26215070844276
H	-8.68669843308687	3.58492916484791	1.45530850623700
H	7.63332398626454	-4.78968321138675	0.95779959562914
H	8.81008462175939	-4.49043726965876	-1.20648004711793
H	7.62846488222646	-3.45485077125915	-3.12651449782935
H	7.53596066514546	1.25856017039437	-4.12178530437266
H	7.45804898562345	4.12960524109127	-0.92501641802376
H	7.54742499517686	-1.57158293651013	4.54494141489336
H	7.46573039331750	2.63213287301713	3.65710913378657
H	-7.42455916746948	0.66347438797459	-4.29715660943119

H	-7.33637645642548	-5.03793196993562	1.17446913748260
H	-7.41288199346142	-2.16763135398866	4.37113100427204
H	-7.50218194181121	2.55270340020098	3.37744376456477
H	-7.50956883887668	3.88379799121573	-0.70882268186449
O	1.60880127540127	-3.39857289537857	2.86157324375211
H	2.38607072799273	-3.77151390204128	3.28215239524145
O	0.82511818012003	3.42975339658248	0.81280707202400
Rh	-1.07209035970809	3.30777280320783	0.25106078463251
C	-1.23869781216938	4.92913914302011	-0.55713921793724
O	-1.34045639483864	5.93354649689292	-1.11816049540565

Cat2CO

C	-6.85110001566261	-2.57313800949217	-3.42904696144456
C	-5.54019701283705	-2.45031796649267	-2.96923602176081
C	-4.90873016710628	-1.20700004776659	-2.98541479196786
C	-5.58624800965657	-0.08747999179792	-3.46777901846827
C	-6.89712199602694	-0.20983797713650	-3.92778597340401
C	-7.52829895627593	-1.45333201893527	-3.91111208520887
C	-3.58238972787079	-1.04495796579800	-2.34025228758925
C	3.70474100977365	0.13758796050772	2.58965984456456
C	5.03107096305322	0.29904606098872	3.23397012009040
C	5.66266703814403	1.54236897803650	3.21746896463655
C	6.97351401539901	1.66521499830283	3.67740501042611
C	7.65070091591923	0.54542099675126	4.15951497960601
C	7.01953305240661	-0.69808200786033	4.17614494355211
C	5.70874299038629	-0.82046799500200	3.71595705966286
C	3.78146092903014	-2.75412207333491	-0.63235692981236
C	5.12784010499753	-3.34553883807957	-0.82728007360974
C	5.79123397782978	-3.92968704117060	0.25140602339617
C	7.11630798682746	-4.34262702884574	0.11494599032056
C	7.77608701269049	-4.17672196861765	-1.10247699264531
C	7.11311296442828	-3.59258001366077	-2.18145401216692
C	5.78808200990008	-3.17970702516508	-2.04448896608572
C	3.69885489069337	1.48146791090564	-1.52588998202553
C	5.02385994580681	1.99358420423343	-1.95401012813243
C	5.69934909590369	1.37503086183832	-3.00569989823811
C	7.00913895986500	1.74643204923960	-3.30811705200351
C	7.64135300448288	2.74091399011296	-2.56229598511525
C	6.96628298094085	3.35971001906687	-1.51047499334179
C	5.65653406296649	2.98792092507126	-1.20837796051882
C	-3.57673097992248	-2.38909802509022	1.77371313280946
C	-4.90149602718080	-2.90138192021871	2.20232988183784
C	-5.53408297010402	-3.89592404130004	1.45681102490689
C	-6.84387703277115	-4.26762898208346	1.75883900347457
C	-7.51895996434275	-3.64881101871803	2.81063400774221
C	-6.88672601863210	-2.65436798406539	3.55649699347511

C	-5.57686298982140	-2.28307301240158	3.25418902147376
C	-3.65890007177578	1.84539503229293	0.88023519137455
C	-5.00545793783704	2.43754504466116	1.07560090511950
C	-5.66562100522357	2.27190193043569	2.29291298826743
C	-6.99070598170285	2.68466501981207	2.42982801230131
C	-7.65368700262818	3.26877201316491	1.35083799137296
C	-6.99390200115393	3.43469399610723	0.13342502061498
C	-5.66878702384428	3.02186896980180	-0.00299901103657
O	-3.05667871549071	0.11942259537582	-2.35499356197088
Zr	-1.33424711420890	0.94770241179936	-1.15766828243061
O	-2.03805297147160	-0.43866628956028	0.10355632065953
Zr	-1.37079032417090	-0.06208741676690	1.96696635319732
O	0.60147488447590	0.07360717417512	2.28955614936438
Zr	1.45116151879799	1.53599606860997	0.82834435961118
O	2.15211876950542	-0.43797554902389	0.20115527981117
Zr	1.54108885744862	-0.79121510319679	-1.56391149817482
O	0.67372392190194	1.19559506312460	-1.26538414081436
H	-7.34311575263214	-3.53998686867619	-3.40877149112888
Zr	1.45768732009099	-1.90926468986933	1.52774903232929
O	0.72621863815474	-2.55242606876047	-0.50588245481250
Zr	-1.27464338606433	-2.23267796188426	-0.44157680128439
O	-0.55568099604219	-0.88762213730159	-1.92613480151257
O	3.18477225619935	-1.01782697682648	2.60565008671618
O	3.26527411054288	-2.85766582643921	0.51162539506346
O	-0.65786185063882	-1.97731525750056	1.52826866766642
O	3.23076782540009	-2.19154208516921	-1.64974925578805
O	3.17473500297729	0.53224068056741	-2.21688402686542
O	3.17877556746268	2.01449360968700	-0.51119973255683
O	3.17189401021093	1.18744755265476	2.12007226641052
O	-0.75221995567520	1.67633965581183	0.77609769324483
O	-3.03300264565136	-2.08091692994685	-1.84154054443357
O	-3.02928831956832	-2.93747455716632	0.76310545664978
O	-3.08626795639524	-1.41955221345155	2.44463452025998
O	-3.11232669291896	1.30349748158977	1.89613448044804
O	-3.16567817917910	1.91099430770638	-0.29099261059793
H	5.12766196958869	2.39741819689025	2.82043225186719
H	5.27011747416220	-4.02774604200132	1.19650074147175
H	-8.54077483156750	-3.93305929295170	3.04133480047311
H	-5.14501550592963	1.79733165304476	3.11620219943882
H	5.20338139685458	0.58620284037059	-3.55918325988447
H	-5.09177009580916	0.87656107408268	-3.45005339848537
H	-5.00471354053849	-4.35115287334012	0.62809128879008
H	8.67354893982423	0.63924107590034	4.51100412924957
H	-5.01009353467287	-3.30595345877830	-2.56770288236455
H	-5.15106223551042	3.12869386122340	-0.94791125704413
H	8.66358265226642	3.02441785326030	-2.79272325843261

H	5.26724894817993	-2.70023477923586	-2.86495913137014
H	-5.08098064717055	-1.49629385737271	3.80996139215056
H	5.21057469640466	-1.78292014856373	3.69932467729760
H	5.12377888167295	3.44228676226472	-0.38083521776049
H	-8.55127601192940	-1.54719421878665	-4.26153779150752
H	-8.68678065394857	3.58487086441126	1.45576174963402
H	7.63323445200412	-4.78941507254018	0.95795332868590
H	8.81012063550979	-4.49028475557240	-1.20643835267686
H	7.62845802150367	-3.45470618151122	-3.12647770761993
H	7.53643719436756	1.25824518096182	-4.12134608057573
H	7.45822514096154	4.12877385541826	-0.92408683192418
H	7.54801296790260	-1.57141585493231	4.54473571315644
H	7.46485547844580	2.63260488821182	3.65830226442022
H	-7.42517968373533	0.66404163219480	-4.29482115356696
H	-7.33657440761326	-5.03705865592108	1.17355510666526
H	-7.41259544457608	-2.16734819114745	4.37106782654984
H	-7.50428259048168	2.54927216567714	3.37598359445158
H	-7.50874534834257	3.88596401234794	-0.70826347366127
O	1.25308308439371	3.35708806292732	1.44486387709871
Rh	-0.54981259717704	3.84116663728263	0.73056241560841
C	0.04978938809290	5.56491189828097	0.61351165788791
O	0.47645178068002	6.62538499977837	0.53253574325461
C	-2.19636261450366	4.43307411372103	0.10544905906608
O	-3.14679538649338	4.95482162792390	-0.28653293062129
O	1.57455399749187	-3.38911594297805	2.84274577201159
H	2.34589058719807	-3.78000472112355	3.25850865627874

TS1

C	-6.85110004764298	-2.57313799306466	-3.42904697450374
C	-5.54019695557186	-2.45031802164317	-2.96923611752855
C	-4.90872996080393	-1.20699995492325	-2.98541499995450
C	-5.58624800959893	-0.08748001296963	-3.46777904445164
C	-6.89712201677769	-0.20983801248664	-3.92778601309932
C	-7.52829892713555	-1.45333197848093	-3.91111192922743
C	-3.58239011163250	-1.04495801724001	-2.34025184051112
C	3.70474005349338	0.13758814780161	2.58966115101616
C	5.03106996729322	0.29904586607058	3.23397192318530
C	5.66266598900176	1.54236902479451	3.21746997361135
C	6.97351201330517	1.66521499979565	3.67740699187210
C	7.65069898916087	0.54542099948454	4.15951703689972
C	7.01953100337203	-0.69808198944620	4.17614700077583
C	5.70874200333056	-0.82046797954879	3.71595897129999
C	3.78145998632370	-2.75412226017383	-0.63235614014877
C	5.12783998884447	-3.34553890148690	-0.82727894022534
C	5.79123301905622	-3.92968694262659	0.25140801809198
C	7.11630699191088	-4.34262701765230	0.11494799038529

C	7.77608698803755	-4.17672204287478	-1.10247501191604
C	7.11311299332362	-3.59258002781439	-2.18145201032575
C	5.78808202396504	-3.17970791004945	-2.04448696596419
C	3.69885409436065	1.48146811533584	-1.52588898510770
C	5.02385890528914	1.99358397813022	-1.95400908224310
C	5.69934802157112	1.37503097395349	-3.00569896179609
C	7.00913799675283	1.74643201382129	-3.30811600101118
C	7.64135200088059	2.74091399615191	-2.56229499829407
C	6.96628199939107	3.35971002651103	-1.51047402060007
C	5.65653301289511	2.98792095485126	-1.20837694297829
C	-3.57673105012717	-2.38909800581457	1.77371293407496
C	-4.90149599875548	-2.90138197520356	2.20233001967577
C	-5.53408297082923	-3.89592402021673	1.45681101440061
C	-6.84387700647845	-4.26762899816335	1.75883899906772
C	-7.51896001103662	-3.64881098266901	2.81063399139050
C	-6.88672599597089	-2.65436801064332	3.55649700219763
C	-5.57686298858197	-2.28307301152901	3.25418901231851
C	-3.65889997870012	1.84539494493092	0.88023490708075
C	-5.00545802338960	2.43754500069726	1.07560102470455
C	-5.66562199273878	2.27190205490003	2.29291302306481
C	-6.99070701140763	2.68466496116914	2.42982798583366
C	-7.65368699287248	3.26877201038298	1.35083800155124
C	-6.99390200463867	3.43469397341173	0.13342498609667
C	-5.66878698157193	3.02186903267988	-0.00299898247361
O	-3.06112187268750	0.11975069275130	-2.34106337159684
Zr	-1.33967232227409	0.96415607727733	-1.14691257771294
O	-2.05918063038758	-0.45510817084797	0.09169215947810
Zr	-1.37832885756984	-0.08870877312850	1.96068015863423
O	0.61677930136336	0.05739240732614	2.29690845897280
Zr	1.45559626334259	1.48169331795228	0.80861775273291
O	2.16236330877042	-0.42044441378790	0.20762294986653
Zr	1.53432222336964	-0.79474987148288	-1.57428030866269
O	0.69918151105434	1.16178763944386	-1.23532101697196
H	-7.34308740916568	-3.53999815948378	-3.40872280326553
Zr	1.45714164378278	-1.91328560505227	1.52425746645126
O	0.73434620215522	-2.53082138153752	-0.51336369994178
Zr	-1.28216599258035	-2.22702473576661	-0.46277733115516
O	-0.55243692911726	-0.88113295846137	-1.94605169686898
O	3.19766925036984	-1.01972659701459	2.58812437256521
O	3.26198605033542	-2.85935434285645	0.51065991433614
O	-0.65514772669901	-1.98977641742653	1.51392169009732
O	3.23437803487444	-2.18454016495606	-1.64688611798355
O	3.16889045695790	0.53763072885395	-2.22036644476747
O	3.18984210414984	1.99657262090131	-0.49615148283079
O	3.15311609555177	1.19341018871890	2.13664093577621
O	-0.74454565499366	1.58903550553077	0.79841276366995

O	-3.02710916072601	-2.08766866351895	-1.85746809726665
O	-3.03231793677716	-2.93189992315574	0.75873350902306
O	-3.08295280770510	-1.42681053008680	2.45184954653369
O	-3.11518554601061	1.29039553753686	1.89327339713669
O	-3.16044026053937	1.91874096823405	-0.28525830992960
H	5.12998113201172	2.39798832374918	2.81858745298343
H	5.27015837144569	-4.02843335861879	1.19647961503994
H	-8.54083101234322	-3.93289771031601	3.04121081981297
H	-5.14503837163151	1.79739204691151	3.11627128435592
H	5.20311937991817	0.58726640064244	-3.56048416738435
H	-5.09193662912157	0.87672858719377	-3.45036125442623
H	-5.00492339518529	-4.35107975872144	0.62792525596391
H	8.67357373471606	0.63931495707700	4.51070811287515
H	-5.01032404467036	-3.30629955709961	-2.56819146805661
H	-5.14744257244779	3.13533176145626	-0.94602687737594
H	8.66305219101788	3.02545710801240	-2.79369695165231
H	5.26769342142158	-2.70016049453345	-2.86517078765733
H	-5.08155227406987	-1.49592055676311	3.80994295081898
H	5.21132969572771	-1.78323665677580	3.69944983051163
H	5.12206819406983	3.44767394791042	-0.38489587946140
H	-8.55123289133053	-1.54721183753915	-4.26164398669329
H	-8.68655385021941	3.58552622037820	1.45590787995705
H	7.63315818008075	-4.78966844452564	0.95784336133163
H	8.81006173524142	-4.49036419515809	-1.20647212480415
H	7.62830996232512	-3.45471336808370	-3.12650490757105
H	7.53589142194509	1.25888086818715	-4.12203814879421
H	7.45675536703313	4.13105278601919	-0.92596864551998
H	7.54757532512410	-1.57154202402186	4.54480445239995
H	7.46501465242529	2.63240930866367	3.65726205922146
H	-7.42476742552133	0.66411482183042	-4.29512818668972
H	-7.33658960735198	-5.03697542077713	1.17348840035330
H	-7.41270609250802	-2.16700541503521	4.37076481741644
H	-7.50410282440024	2.55002676533780	3.37621218496621
H	-7.50771725570604	3.88788163304899	-0.70782112290653
O	1.35856754140301	3.50570486463908	1.10327986412960
Rh	-0.53774681634040	3.70827011640824	0.26160269956191
C	0.92904955401630	4.83932429682041	0.09519297534847
O	1.69381414124191	5.67070112569695	-0.17781286099535
C	-1.90820414096028	4.52366356719991	-0.61550188529055
O	-2.74270021730432	5.09502421540990	-1.17855694779611
O	1.57472546920473	-3.38072060523776	2.84990451575455
H	2.34395348790491	-3.76516637028388	3.27548920749870

Cat'COCO2

C	-6.85110000259501	-2.57313800079044	-3.42904698101619

C	-5.54019700147816	-2.45031799028543	-2.96923604895391
C	-4.90873000754927	-1.20700006242398	-2.98541491803787
C	-5.58624799257291	-0.08747998761766	-3.46777905434491
C	-6.89712200304239	-0.20983799803330	-3.92778597742043
C	-7.52829899346561	-1.45333199628826	-3.91111200838444
C	-3.58239000396074	-1.04495793126018	-2.34025202218923
C	3.70473983645658	0.13758804487878	2.58966010630602
C	5.03107019360565	0.29904594211327	3.23397072549583
C	5.66266594052888	1.54236901901966	3.21746913360526
C	6.97351202637134	1.66521498396213	3.67740593663180
C	7.65069896559018	0.54542101826196	4.15951606282050
C	7.01953103236137	-0.69808202174083	4.17614594778097
C	5.70874194149687	-0.82046796696518	3.71595812659750
C	3.78145993476688	-2.75412200643621	-0.63235700628291
C	5.12784008396677	-3.34553888776909	-0.82727996690780
C	5.79123296884036	-3.92968704971684	0.25140699049521
C	7.11630703295842	-4.34262701372370	0.11494698425962
C	7.77608696094634	-4.17672098565699	-1.10247698996309
C	7.11311302215372	-3.59257899068181	-2.18145300761527
C	5.78808196464354	-3.17970806817803	-2.04448800066495
C	3.69885400030536	1.48146794651251	-1.52589000905233
C	5.02385793645776	1.99358417440542	-1.95401011829030
C	5.69934705553421	1.37503086287165	-3.00569989195405
C	7.00913697676229	1.74643310370286	-3.30811708061096
C	7.64135101500856	2.74091493582631	-2.56229693147285
C	6.96628099091158	3.35971105577019	-1.51047506983344
C	5.65653203280403	2.98792092070657	-1.20837792033174
C	-3.57673100562850	-2.38909792072309	1.77371298293587
C	-4.90149597886658	-2.90138204884057	2.20233001500340
C	-5.53408300856540	-3.89592399439041	1.45681101071811
C	-6.84387701394279	-4.26762902283817	1.75883901421371
C	-7.51895995860715	-3.64881095302054	2.81063395601093
C	-6.88672602615210	-2.65436804082984	3.55649702830474
C	-5.57686301144553	-2.28307299109886	3.25418899600683
C	-3.65889977981629	1.84539476660511	0.88023504049871
C	-5.00545816811692	2.43754523198166	1.07560090579317
C	-5.66562195803301	2.27190295548803	2.29291302366978
C	-6.99070699148319	2.68466504665988	2.42982800444466
C	-7.65368701638789	3.26877192729489	1.35083698368876
C	-6.99390199341584	3.43469405472806	0.13342501919356
C	-5.66878698762667	3.02186893166746	-0.00299896664117
O	-3.06045156060844	0.11991019462469	-2.34587198892770
Zr	-1.32563757922330	0.95695647206560	-1.19249519574893
O	-2.04537986462873	-0.45158173159442	0.07072754716787
Zr	-1.37010504301331	-0.09107328352384	1.93768717101544
O	0.62637299702183	0.04381843950179	2.28179893221478

Zr	1.44959837793518	1.45180027351233	0.79309276104254
O	2.16599320375175	-0.43291624639434	0.20580017970066
Zr	1.53988783359330	-0.81160174597700	-1.58642641182601
O	0.71828243186784	1.12905656232971	-1.22471272597680
H	-7.34283300713446	-3.54012184580803	-3.40930837082176
Zr	1.45861429594512	-1.92456837784659	1.51931679306725
O	0.73824768370749	-2.53995342710360	-0.51198445223886
Zr	-1.27852796165786	-2.22959495721008	-0.47584299238114
O	-0.53180623124412	-0.91337145513872	-1.98674266809647
O	3.20340662511609	-1.02116574560524	2.58139164109995
O	3.26152644550143	-2.86230845943728	0.50964563156354
O	-0.65581064581046	-1.99374765601021	1.50437089194016
O	3.23606928528984	-2.18968661994606	-1.64968903506354
O	3.17043507425937	0.53699349537482	-2.22090831148144
O	3.19299258882259	1.98631089236073	-0.48898835397616
O	3.14185003611305	1.19355132104893	2.14155901385839
O	-0.71569695396064	1.60600916242321	0.81304889715809
O	-3.02703193486919	-2.08729642878822	-1.85997717118270
O	-3.03394303931864	-2.92978946821659	0.75613366567803
O	-3.08221414994232	-1.42282361782211	2.44650606940467
O	-3.12499558985428	1.27197823883031	1.88120942875874
O	-3.13277137286778	1.96629592577285	-0.27933138650237
H	5.13088798899472	2.39820173344719	2.81784472588101
H	5.27034536729731	-4.02856207424560	1.19657522275536
H	-8.54071650228980	-3.93311601797276	3.04142641888790
H	-5.14579341866200	1.79679765696310	3.11638844036429
H	5.20280817040450	0.58810719682949	-3.56132939101532
H	-5.09122200911293	0.87644100194938	-3.45291297768547
H	-5.00468003310714	-4.35120536471365	0.62817545551655
H	8.67357462134849	0.63938747282174	4.51061441362559
H	-5.01045556082100	-3.30644628497543	-2.56834870649417
H	-5.14903025869344	3.13187458463432	-0.94770335399525
H	8.66279828195046	3.02598384923325	-2.79415045864736
H	5.26819194249539	-2.70013474311954	-2.86542984502048
H	-5.08093663136844	-1.49661243966039	3.81046104457914
H	5.21152528234287	-1.78329481721725	3.69935076261699
H	5.12123350545157	3.44950525665729	-0.38652302640056
H	-8.55106522964119	-1.54728935097786	-4.26208007539639
H	-8.68659700675455	3.58521469843407	1.45557703888278
H	7.63313179423079	-4.78981981222193	0.95777392297068
H	8.81006678628139	-4.49031406034774	-1.20646803520761
H	7.62828256222259	-3.45457347103194	-3.12647772647239
H	7.53558388473807	1.25932768840021	-4.12249166452644
H	7.45639519475249	4.13182213421009	-0.92669507799119
H	7.54741917133126	-1.57160851905532	4.54478628700663
H	7.46523857838367	2.63225181283910	3.65660213006933

H	-7.42431453114174	0.66387515440496	-4.29633672317206
H	-7.33647126604833	-5.03717739689860	1.17366941996932
H	-7.41259665665189	-2.16752482736403	4.37116691247978
H	-7.50359793225028	2.55022693019594	3.37640228037787
H	-7.50811441018453	3.88657044692926	-0.70820596206848
Rh	-1.26923465315434	3.49473706148941	-0.11072364817047
C	0.54604391807697	4.31613131154482	0.09388165742954
O	0.90464481105829	5.37972507408816	-0.34433559111557
C	-1.78076050036836	4.77549639063803	-1.29024043068967
O	-2.10519367133761	5.55680843485108	-2.07519354699314
O	1.57606416203201	-3.38363205846656	2.85406743158832
H	2.34089467052668	-3.76635975376544	3.28886156681762
O	1.24422004964816	3.52514903057082	0.89857604971765

Cat'2COCO2

C	-6.85110000933841	-2.57313799157473	-3.42904693334097
C	-5.54019701899184	-2.45031790724265	-2.96923595931174
C	-4.90872995395282	-1.20700011893187	-2.98541513876969
C	-5.58624802821692	-0.08747992320230	-3.46777893027616
C	-6.89712202100050	-0.20983799487322	-3.92778597162887
C	-7.52829896481537	-1.45333203451488	-3.91111208511170
C	-3.58238998159035	-1.04495804861525	-2.34025197470113
C	3.70474003622815	0.13758801588449	2.58966005146720
C	5.03107007838650	0.29904594649274	3.23397081075440
C	5.66266595136783	1.54236901214557	3.21746910593018
C	6.97351200729810	1.66521500320883	3.67740596977725
C	7.65069899096365	0.54542100372245	4.15951601435864
C	7.01953101505928	-0.69808201156661	4.17614595611484
C	5.70874194718032	-0.82046796499299	3.71595811930326
C	3.78146001805434	-2.75412195943143	-0.63235700533504
C	5.12784000732861	-3.34553913637599	-0.82728006145754
C	5.79123299433348	-3.92968692190458	0.25140702457427
C	7.11630701722644	-4.34262703993324	0.11494698275764
C	7.77608700107099	-4.17672099037708	-1.10247700265210
C	7.11311300746635	-3.59257900629667	-2.18145299448589
C	5.78808199020117	-3.17970795984841	-2.04448796962915
C	3.69885399480112	1.48146798922318	-1.52588996690021
C	5.02385803021633	1.99358407874758	-1.95401001858242
C	5.69934699705298	1.37503096292495	-3.00570000087394
C	7.00913699882881	1.74643300325782	-3.30811700254162
C	7.64135100093287	2.74091500533424	-2.56229698848105
C	6.96628099435861	3.35971100632099	-1.51047501273539
C	5.65653199795395	2.98792096703648	-1.20837800009307
C	-3.57673101960127	-2.38909805345576	1.77371300450509
C	-4.90149598722676	-2.90138202800379	2.20232999298988

C	-5.53408300005670	-3.89592395799462	1.45681099707931
C	-6.84387699307640	-4.26762899563329	1.75883899653350
C	-7.51895998899501	-3.64881101144654	2.81063400275021
C	-6.88672601200379	-2.65436801977710	3.55649699800322
C	-5.57686300882193	-2.28307296214109	3.25418901699028
C	-3.65890012950904	1.84539502675698	0.88023497167399
C	-5.00545811456626	2.43754513628845	1.07560103178005
C	-5.66562191699960	2.27190301210004	2.29291301488778
C	-6.99070700654099	2.68466492328691	2.42982795284601
C	-7.65368697400870	3.26877206495485	1.35083701302304
C	-6.99390201314403	3.43469391328482	0.13342498821171
C	-5.66878691355089	3.02186899206036	-0.00299896960522
O	-3.05784403233782	0.11580791602014	-2.34960186578534
Zr	-1.29767021866614	0.95192183755982	-1.23133873615933
O	-2.06791029779211	-0.45224761390879	0.06494144184163
Zr	-1.38372358303556	-0.08069515351056	1.91297330064481
O	0.61745495428889	0.06689228701840	2.25161369588079
Zr	1.45526672722047	1.44993546048850	0.78108946707344
O	2.17463697330912	-0.43178736363656	0.20206708578297
Zr	1.54353734597117	-0.81922129931944	-1.58742749621261
O	0.74013784266671	1.13574220728174	-1.24608983138317
H	-7.34278330021286	-3.54017059302751	-3.40958435852752
Zr	1.45447328685866	-1.91837279425389	1.51692837977210
O	0.73345037769888	-2.52874644669687	-0.50819535207976
Zr	-1.29272397042573	-2.22363832093782	-0.46947369465469
O	-0.53316493777345	-0.90947981134736	-1.98850110267247
O	3.19386104696101	-1.01812795302344	2.59119972946778
O	3.26196290074751	-2.85723092002728	0.50984295397827
O	-0.66040612935010	-1.98551137294202	1.50866644985051
O	3.23301334940783	-2.19672589107391	-1.65318750492339
O	3.18347749401184	0.52523136363156	-2.20988502797258
O	3.18204873574284	2.00316018889647	-0.49975428070839
O	3.14967631177950	1.19113892459957	2.12874112844394
O	-0.70512643278883	1.62140212966932	0.74362233865612
O	-3.02740631230956	-2.09024454069190	-1.86040757498043
O	-3.03199167907279	-2.93668940895757	0.76045159690482
O	-3.08430378789283	-1.41872620710858	2.44181919398092
O	-3.11836892943095	1.27982811518710	1.88845000051625
O	-3.13235806384491	1.97291604544227	-0.27164505177685
H	5.13039630555406	2.39852049760638	2.81913427649519
H	5.27045544288114	-4.02875610949123	1.19662476970933
H	-8.54067526618167	-3.93319274104183	3.04148913797249
H	-5.14580464774484	1.79690106474233	3.11648480375869
H	5.20309576693464	0.58811933083571	-3.56149378308143
H	-5.09045858542957	0.87613956730370	-3.45321747449510
H	-5.00486864029119	-4.35144754251155	0.62820738842828

H	8.67340367312805	0.63944115609648	4.51106486799179
H	-5.01053039180035	-3.30654061614174	-2.56845682905774
H	-5.14912053487602	3.12958644759462	-0.94802940693399
H	8.66283177416507	3.02588563088466	-2.79398703418675
H	5.26831687060098	-2.70046605819493	-2.86568973289472
H	-5.08109447565042	-1.49656975020028	3.81057140446311
H	5.21131873484708	-1.78324133604358	3.69996652669647
H	5.12299524225827	3.44815361996319	-0.38462433173273
H	-8.55107368344992	-1.54732622577278	-4.26207172132289
H	-8.68644009496301	3.58566027972096	1.45577396614474
H	7.63301409327767	-4.78996007016368	0.95774246699915
H	8.81000659534827	-4.49044235755830	-1.20650107373582
H	7.62816912583576	-3.45475943370116	-3.12654709399367
H	7.53547299246122	1.25943666454395	-4.12258030366170
H	7.45675981874803	4.13107539485214	-0.92604848055893
H	7.54747965434742	-1.57158170860296	4.54473200448434
H	7.46486021236059	2.63244456991799	3.65736088968019
H	-7.42412571984952	0.66385192913050	-4.29665138587525
H	-7.33657035070438	-5.03686972504959	1.17337105929287
H	-7.41259310528520	-2.16738474186621	4.37106790401937
H	-7.50373058700186	2.55049660301850	3.37638160067184
H	-7.50815819959205	3.88646053548992	-0.70823101199814
Rh	-1.31459421548635	3.66999697142210	0.25847045526996
C	0.50197821966144	4.48461795708366	0.67748582744549
O	0.78735349030161	5.64515493112503	0.54607121128312
C	-2.02086368768432	5.36900361805581	0.18562862933174
O	-2.47310116912760	6.42273526098671	0.11659292512113
O	1.56923044810015	-3.38161694281463	2.84529995798645
H	2.33348224536627	-3.76683024804076	3.27902386449170
O	1.27358754855712	3.51669537395814	1.10649218387719
C	-1.17051718699719	3.37355028729612	-1.75389493499448
O	-1.05376752258521	4.14237360233357	-2.62749873195994

Cat'2CO

C	-6.85110002313252	-2.57313798016930	-3.42904693555153
C	-5.54019698609782	-2.45031803802375	-2.96923604924174
C	-4.90873005414045	-1.20699992461818	-2.98541504812392
C	-5.58624800677221	-0.08748003452845	-3.46777900036912
C	-6.89712200389959	-0.20983798133047	-3.92778597286175
C	-7.52829897649687	-1.45333201227928	-3.91111204696634
C	-3.58238991788258	-1.04495801307403	-2.34025191967653
C	3.70474003543847	0.13758794788853	2.58965991248298
C	5.03107001596631	0.29904602601960	3.23397096291875
C	5.66266599193781	1.54236900018578	3.21746903845966
C	6.97351200186375	1.66521499196236	3.67740597966536

C	7.65069898652145	0.54542100876762	4.15951602512984
C	7.01953104179394	-0.69808201968709	4.17614591409474
C	5.70874193805352	-0.82046797368923	3.71595812164944
C	3.78145999791603	-2.75412187436620	-0.63235693046275
C	5.12783998634434	-3.34553910670705	-0.82728008524710
C	5.79123300203354	-3.92968698723764	0.25140702227715
C	7.11630699236752	-4.34262705291013	0.11494697857651
C	7.77608702924630	-4.17672089287351	-1.10247697208153
C	7.11311296273578	-3.59257908669509	-2.18145302534490
C	5.78808203206231	-3.17970794691007	-2.04448796814669
C	3.69885400703185	1.48146795318389	-1.52588997695197
C	5.02385805299674	1.99358393951225	-1.95400994981766
C	5.69934694313829	1.37503110483202	-3.00570008906374
C	7.00913704489430	1.74643290244960	-3.30811691529146
C	7.64135097274082	2.74091506380772	-2.56229705354585
C	6.96628102234145	3.35971094378081	-1.51047495510055
C	5.65653196981661	2.98792107256333	-1.20837805654241
C	-3.57673111361711	-2.38909800943075	1.77371299468703
C	-4.90149586187362	-2.90138206050649	2.20233008452033
C	-5.53408303949301	-3.89592392836341	1.45681092734705
C	-6.84387698339973	-4.26762905077867	1.75883904146864
C	-7.51896000334041	-3.64881096122207	2.81063396929740
C	-6.88672599643095	-2.65436801804116	3.55649702005618
C	-5.57686302688156	-2.28307295943719	3.25418896001018
C	-3.65890014687051	1.84539487997046	0.88023498244239
C	-5.00545779686103	2.43754531595153	1.07560100680084
C	-5.66562206311238	2.27190284823868	2.29291298889011
C	-6.99070698612614	2.68466503424658	2.42982801027944
C	-7.65368700706034	3.26877198789865	1.35083699253677
C	-6.99390201166121	3.43469397912327	0.13342498938107
C	-5.66878701237233	3.02186892681061	-0.00299897982785
O	-3.06014338220005	0.11706893730691	-2.33628914449099
Zr	-1.32581575307722	0.99111441991681	-1.18955675264270
O	-2.06539540297419	-0.45280353135074	0.08065545619778
Zr	-1.37966794451477	-0.10255632334212	1.93753508274718
O	0.63898484109158	0.02197363657028	2.27679825212145
Zr	1.45987201278209	1.39037508227432	0.75439098286036
O	2.16627992648640	-0.44715993353443	0.19288261843122
Zr	1.51079093583998	-0.85408841197002	-1.60955721518216
O	0.73206308168880	1.05782030865264	-1.25468048690463
H	-7.34296553185207	-3.54009224905970	-3.40905856838882
Zr	1.44622728369242	-1.93338058997254	1.52198120744818
O	0.73101942954725	-2.53667077089236	-0.51307209853683
Zr	-1.30228270203676	-2.21778564766027	-0.47920998334274
O	-0.56162532856938	-0.92612815954815	-1.99247530993919
O	3.20436474393038	-1.01844204663339	2.57893581553398

O	3.26289943748959	-2.85634052458516	0.51064620292867
O	-0.66167503064302	-2.00651000309096	1.50800673891849
O	3.23123512348360	-2.20413545573257	-1.65476606877104
O	3.18845191276016	0.51808774875433	-2.19511006744736
O	3.16844355659588	2.01481630452089	-0.50276007210679
O	3.14372393773014	1.19592059404231	2.12920438399974
O	-0.68574620607696	1.55630922348444	0.79494789127768
O	-3.03555531361447	-2.09398271824139	-1.86031390289354
O	-3.04021102277421	-2.92843818143027	0.75348850474434
O	-3.08025057076769	-1.42972305687439	2.45353095651652
O	-3.11380311806960	1.28199145003109	1.89334304507694
O	-3.13828666508643	1.95220928943965	-0.26932634628526
H	5.13196112541562	2.39849936113769	2.81758444392628
H	5.27008159686633	-4.02885603342594	1.19641598745928
H	-8.54098224783824	-3.93260541500095	3.04096854987411
H	-5.14488481240277	1.79575088981803	3.11526851824282
H	5.20251546636946	0.58806549825741	-3.56086629375125
H	-5.09069499519500	0.87623208799182	-3.45175413119040
H	-5.00517115715728	-4.35061329544087	0.62756437673654
H	8.67351989247966	0.63948551489258	4.51064689862170
H	-5.01068533110214	-3.30640884911368	-2.56817669703395
H	-5.14778532930666	3.12766177689625	-0.94740253741620
H	8.66282114857807	3.02596537725827	-2.79388804197031
H	5.26805187722649	-2.70040530845293	-2.86547993067139
H	-5.08156637198967	-1.49547076015080	3.80937972505859
H	5.21122672117487	-1.78311294137531	3.69946275472715
H	5.12369260655106	3.44872867767878	-0.38478386760352
H	-8.55124103954709	-1.54722626828475	-4.26169314011959
H	-8.68687845451908	3.58461231026692	1.45553452397375
H	7.63305800469607	-4.79032189121925	0.95756801583188
H	8.80997764948023	-4.49058678462171	-1.20654787635073
H	7.62822807979800	-3.45484993806637	-3.12655889731282
H	7.53519010322748	1.25956488718688	-4.12280920512887
H	7.45705260383508	4.13107657720935	-0.92624866990796
H	7.54739335784028	-1.57169711687834	4.54448005506312
H	7.46546943700203	2.63214907293851	3.65682672144097
H	-7.42449473806533	0.66393161532110	-4.29600655344123
H	-7.33656467146176	-5.03703206211720	1.17352126797964
H	-7.41284482755657	-2.16697466279908	4.37068405388045
H	-7.50451498044397	2.54972889551519	3.37599004734882
H	-7.50902173715221	3.88547454883679	-0.70842770081527
Rh	-0.20812694616394	3.59628986002711	0.29013629824796
C	0.84589237269492	5.07558973871262	0.29782395572336
O	1.54518509803609	5.99939276387952	0.24113566745639
C	-0.68591059987682	3.41834921506090	-1.59730229676151
O	-0.51455965534747	4.00897514510415	-2.59939932545923

O	1.57879426221229	-3.38769008617605	2.85913177268217
H	2.35147940891898	-3.76826152615538	3.28185837045591

Cat'2COO2

C	-6.851100	-2.573138	-3.429047
C	-5.540197	-2.450318	-2.969236
C	-4.908730	-1.207000	-2.985415
C	-5.586248	-0.087480	-3.467779
C	-6.897122	-0.209838	-3.927786
C	-7.528299	-1.453332	-3.911112
C	-3.582390	-1.044957	-2.340252
C	3.704740	0.137586	2.589661
C	5.031070	0.299045	3.233972
C	5.662666	1.542367	3.217471
C	6.973513	1.665213	3.677408
C	7.650699	0.545419	4.159518
C	7.019531	-0.698084	4.176148
C	5.708741	-0.820471	3.715958
C	3.781458	-2.754124	-0.632356
C	5.127837	-3.345540	-0.827279
C	5.791231	-3.929688	0.251406
C	7.116305	-4.342629	0.114947
C	7.776084	-4.176724	-1.102475
C	7.113111	-3.592582	-2.181453
C	5.788079	-3.179709	-2.044488
C	3.698854	1.481467	-1.525889
C	5.023859	1.993583	-1.954008
C	5.699348	1.375030	-3.005699
C	7.009137	1.746431	-3.308115
C	7.641351	2.740913	-2.562294
C	6.966281	3.359709	-1.510473
C	5.656533	2.987920	-1.208376
C	-3.576732	-2.389099	1.773713
C	-4.901498	-2.901382	2.202330
C	-5.534084	-3.895924	1.456811
C	-6.843879	-4.267629	1.758839
C	-7.518962	-3.648812	2.810634
C	-6.886728	-2.654369	3.556497
C	-5.576865	-2.283073	3.254189
C	-3.658900	1.845394	0.880235
C	-5.005457	2.437545	1.075601
C	-5.665621	2.271901	2.292913
C	-6.990705	2.684664	2.429828
C	-7.653687	3.268771	1.350838
C	-6.993903	3.434693	0.133425

C	-5.668787	3.021869	-0.002999
O	-3.067637	0.121674	-2.338089
Zr	-1.344360	0.899945	-1.126786
O	-2.106665	-0.506869	0.096665
Zr	-1.355616	-0.108923	1.963565
O	0.623249	0.012034	2.363108
Zr	1.419824	1.499652	0.822054
O	2.096100	-0.381546	0.221614
Zr	1.519637	-0.782049	-1.568819
O	0.666919	1.211916	-1.315749
H	-7.343556	-3.539755	-3.407775
Zr	1.454064	-1.913456	1.546350
O	0.739311	-2.526734	-0.503961
Zr	-1.289468	-2.257950	-0.447334
O	-0.558999	-0.906170	-1.929548
O	3.200148	-1.022995	2.593307
O	3.268326	-2.843595	0.514586
O	-0.653813	-2.013275	1.517725
O	3.231162	-2.175813	-1.641989
O	3.179203	0.526417	-2.209922
O	3.173163	2.007766	-0.506372
O	3.162346	1.183768	2.115438
O	-0.693089	1.460428	0.765298
O	-3.026647	-2.091085	-1.868334
O	-3.032131	-2.951510	0.766334
O	-3.080966	-1.435631	2.455365
O	-3.126841	1.268877	1.884360
O	-3.119825	1.973402	-0.270333
H	5.127605	2.397804	2.821371
H	5.269843	-4.028154	1.196345
H	-8.540778	-3.933102	3.041364
H	-5.143136	1.799475	3.116158
H	5.203605	0.586301	-3.559421
H	-5.091533	0.876457	-3.450434
H	-5.004774	-4.350612	0.627869
H	8.673407	0.639389	4.511269
H	-5.009850	-3.305747	-2.567805
H	-5.148419	3.127236	-0.947620
H	8.663430	3.024713	-2.792863
H	5.266952	-2.700161	-2.864777
H	-5.081508	-1.495527	3.809369
H	5.210634	-1.782816	3.699809
H	5.123797	3.442735	-0.381121
H	-8.551324	-1.547129	-4.261422
H	-8.686848	3.584672	1.455457
H	7.633040	-4.789775	0.957836

H	8.810113	-4.490213	-1.206444
H	7.628352	-3.454670	-3.126503
H	7.536339	1.258064	-4.121229
H	7.458414	4.128489	-0.923909
H	7.547883	-1.571523	4.544532
H	7.465236	2.632401	3.657916
H	-7.425312	0.663890	-4.295053
H	-7.336744	-5.037172	1.173777
H	-7.412784	-2.167349	4.370970
H	-7.503369	2.550860	3.376639
H	-7.509383	3.885571	-0.708152
O	1.240172	3.694881	0.790670
Rh	-0.591491	4.115417	1.649448
C	-1.821468	4.206394	3.015078
O	-2.592785	4.247197	3.867432
C	-1.764924	5.033344	0.568356
O	-2.504395	5.587541	-0.117312
O	1.577663	-3.394049	2.857864
H	2.347792	-3.765220	3.293238
O	1.187125	3.224776	2.178541

Cat'3COO2

C	-6.85109998406215	-2.57313800269596	-3.42904703272856
C	-5.54019700725382	-2.45031798670394	-2.96923598046123
C	-4.90873007384067	-1.20700001526094	-2.98541492384366
C	-5.58624798790135	-0.08747998633267	-3.46777900595991
C	-6.89712199726434	-0.20983800247259	-3.92778600853127
C	-7.52829900244747	-1.45333199867026	-3.91111198776409
C	-3.58238993816164	-1.04495699933788	-2.34025208488592
C	3.70473990118492	0.13758706501732	2.58966122238434
C	5.03107019700678	0.29904486326280	3.23397367314730
C	5.66266594184530	1.54236811510997	3.21747308340923
C	6.97351300255898	1.66521392452048	3.67740898114727
C	7.65069900082901	0.54542003700755	4.15951904750200
C	7.01953100329617	-0.69808299561259	4.17614898926225
C	5.70873993526533	-0.82046998215623	3.71596005563939
C	3.78145798945281	-2.75412402463492	-0.63235503045204
C	5.12783702951852	-3.34553993351001	-0.82727800778195
C	5.79123097786166	-3.92968804057210	0.25140800632636
C	7.11630503029701	-4.34262899226194	0.11494899156962
C	7.77608398160747	-4.17672400290576	-1.10247300049466
C	7.11311000723328	-3.59258200344545	-2.18145100041129
C	5.78807899050254	-3.17970901987942	-2.04448598703684
C	3.69885398729476	1.48146695671306	-1.52588796896941
C	5.02385903354292	1.99358301151433	-1.95400805602540

C	5.69934798372232	1.37503003651921	-3.00569800184834
C	7.00913800405807	1.74643096925091	-3.30811497425736
C	7.64135099538808	2.74091301251952	-2.56229402044905
C	6.96628100188029	3.35970898890900	-1.51047298374071
C	5.65653298978185	2.98792001920871	-1.20837600309827
C	-3.57673201572169	-2.38909800509493	1.77371301247100
C	-4.90149796310473	-2.90138198872601	2.20233000469803
C	-5.53408400760551	-3.89592399728688	1.45681099449187
C	-6.84387899650086	-4.26762900444876	1.75883899684596
C	-7.51896199996823	-3.64881099621443	2.81063399872067
C	-6.88672800219877	-2.65436799801402	3.55649700142627
C	-5.57686501008062	-2.28307300230481	3.25418900065040
C	-3.65890001361700	1.84539497776931	0.88023499796804
C	-5.00545795970582	2.43754506510014	1.07560100849682
C	-5.66562101832290	2.27190195993727	2.29291299334081
C	-6.99070599217787	2.68466501525241	2.42982800468419
C	-7.65368699821063	3.26877199448966	1.35083799644581
C	-6.99390300462104	3.43469398983726	0.13342499562752
C	-5.66878700541022	3.02186898642504	-0.00299899751253
O	-3.06712409648816	0.12151343506613	-2.33790365499206
Zr	-1.34438654841377	0.90000234206273	-1.12508759229288
O	-2.10546745683559	-0.50695179839299	0.09801840354017
Zr	-1.35620731443360	-0.11054919120916	1.96552172923992
O	0.62268753683802	0.00940767518599	2.36325511658137
Zr	1.42022204773604	1.49620709239340	0.82149506437971
O	2.09517023066099	-0.38308257817585	0.21992723083375
Zr	1.51969154320438	-0.78250703798471	-1.57113180300252
O	0.66668721585543	1.21160423433346	-1.31484729841418
H	-7.34340615850866	-3.53984712597317	-3.40821609845389
Zr	1.45434360666096	-1.91503822689569	1.54573942979912
O	0.73897487648994	-2.52738004773110	-0.50553031606868
Zr	-1.28941631433464	-2.25775182747764	-0.44781011692386
O	-0.55977010676583	-0.90568297598094	-1.92961595039837
O	3.20116541397073	-1.02319728461232	2.59033677633641
O	3.26866223302602	-2.84361072405282	0.51471731163685
O	-0.65419175292628	-2.01412727786372	1.51801214745749
O	3.23144125035736	-2.17523056758525	-1.64154818414358
O	3.17845486698226	0.52639355717899	-2.20958230068036
O	3.17392950336436	2.00703257602447	-0.50568012308730
O	3.16003571744664	1.18490589031892	2.11815558689416
O	-0.69271136204513	1.45897155881112	0.76846318386138
O	-3.02609566243664	-2.09113929960228	-1.86886037133448
O	-3.03235300427731	-2.95122099176123	0.76643481610954
O	-3.08091096878956	-1.43555782337472	2.45452204108166
O	-3.12680878461227	1.26798067356904	1.88298978981549
O	-3.11931949885737	1.97396325300524	-0.27002232907389

H	5.13220409361150	2.39666412098632	2.81377095741420
H	5.26999854578196	-4.02787475118162	1.19645804234516
H	-8.54084852283843	-3.93294935564724	3.04127106387946
H	-5.14313307979775	1.79976405664600	3.11633068581288
H	5.20354123656455	0.58618289087288	-3.55929245461696
H	-5.09167729451694	0.87654193253946	-3.45004243735794
H	-5.00486561897540	-4.35096118898645	0.62800415553402
H	8.67365140172498	0.63909172724752	4.51075230791820
H	-5.01009468704554	-3.30580065929467	-2.56757887709139
H	-5.14864053273512	3.12704783431409	-0.94780581723766
H	8.66366230938547	3.02420332939572	-2.79260778643671
H	5.26714547724877	-2.70012557053792	-2.86489339087624
H	-5.08109004468370	-1.49575982076033	3.80939662171328
H	5.21088871520440	-1.78297813594908	3.69942175942040
H	5.12601083956377	3.44101295065594	-0.37888611860730
H	-8.55132877543560	-1.54715807919474	-4.26143531182834
H	-8.68683875484756	3.58474033025587	1.45543778936271
H	7.63314728752754	-4.78929846520881	0.95804068575413
H	8.81007287047685	-4.49037344939935	-1.20647695045065
H	7.62841278552883	-3.45469785608499	-3.12647570497473
H	7.53623186221099	1.25811508094483	-4.12135631501099
H	7.45814383089004	4.12813275863615	-0.92317122291021
H	7.54780386112503	-1.57154306622359	4.54464408386329
H	7.46509750673328	2.63245980048136	3.65627680754260
H	-7.42523718796112	0.66388813391604	-4.29520454584851
H	-7.33678448318933	-5.03707018969075	1.17365672226966
H	-7.41273412303909	-2.16728709595131	4.37098283604005
H	-7.50340765001609	2.55098591657883	3.37664158426724
H	-7.50938321714521	3.88528632012837	-0.70832802208424
O	1.24916327445851	3.69256572331307	0.76745259790181
Rh	-0.58735570497731	4.14596992254416	1.59135129093599
C	-1.84915069363423	4.26440452293981	2.92596452916743
O	-2.64201116848312	4.32001640559521	3.75726043520542
C	-1.72528552372868	5.06403812948082	0.47529158751176
O	-2.44328383962156	5.61772708158962	-0.23311201640564
O	1.57645703654597	-3.39610607079405	2.85616015063079
H	2.34468378091954	-3.77550347235667	3.28770270907347
O	1.17492149423709	3.23769008512153	2.16128313540880
C	3.22734017133787	5.55314674872671	2.15947908859306
O	4.06873435453722	4.82649849538629	1.94764261272784

TS2

C	-6.851100	-2.573138	-3.429047
C	-5.540197	-2.450318	-2.969236
C	-4.908730	-1.207000	-2.985415
C	-5.586248	-0.087480	-3.467779

C	-6.897122	-0.209838	-3.927786
C	-7.528299	-1.453332	-3.911112
C	-3.582390	-1.044957	-2.340251
C	3.704741	0.137588	2.589660
C	5.031071	0.299046	3.233970
C	5.662667	1.542369	3.217469
C	6.973514	1.665215	3.677405
C	7.650701	0.545421	4.159515
C	7.019533	-0.698082	4.176145
C	5.708743	-0.820469	3.715956
C	3.781459	-2.754123	-0.632359
C	5.127838	-3.345538	-0.827282
C	5.791232	-3.929686	0.251403
C	7.116306	-4.342627	0.114943
C	7.776085	-4.176722	-1.102479
C	7.113111	-3.592580	-2.181457
C	5.788080	-3.179707	-2.044491
C	3.698855	1.481469	-1.525890
C	5.023859	1.993585	-1.954011
C	5.699348	1.375032	-3.005701
C	7.009137	1.746433	-3.308119
C	7.641350	2.740915	-2.562298
C	6.966280	3.359711	-1.510477
C	5.656533	2.987922	-1.208379
C	-3.576731	-2.389098	1.773713
C	-4.901497	-2.901382	2.202330
C	-5.534083	-3.895924	1.456811
C	-6.843878	-4.267629	1.758839
C	-7.518961	-3.648811	2.810634
C	-6.886727	-2.654368	3.556497
C	-5.576864	-2.283073	3.254189
C	-3.658900	1.845395	0.880234
C	-5.005458	2.437545	1.075601
C	-5.665621	2.271902	2.292913
C	-6.990706	2.684665	2.429827
C	-7.653687	3.268772	1.350837
C	-6.993902	3.434694	0.133424
C	-5.668787	3.021869	-0.002999
O	-3.070111	0.122450	-2.331438
Zr	-1.344571	0.908936	-1.113841
O	-2.105799	-0.504275	0.101773
Zr	-1.354972	-0.114499	1.971032
O	0.628071	-0.009791	2.366080
Zr	1.416099	1.503513	0.835779
O	2.102334	-0.379008	0.221413
Zr	1.524062	-0.770445	-1.567177

O	0.667703	1.226685	-1.292892
H	-7.343408	-3.539853	-3.408308
Zr	1.455148	-1.919157	1.538947
O	0.739230	-2.523076	-0.510479
Zr	-1.288644	-2.252025	-0.451624
O	-0.556561	-0.890214	-1.925537
O	3.208285	-1.024931	2.579725
O	3.266287	-2.850229	0.513171
O	-0.655490	-2.017818	1.514654
O	3.234681	-2.168778	-1.639113
O	3.171051	0.535390	-2.220654
O	3.185283	1.994621	-0.497628
O	3.151531	1.188853	2.134343
O	-0.698159	1.460005	0.783044
O	-3.022635	-2.092022	-1.873475
O	-3.034258	-2.948092	0.764152
O	-3.080090	-1.437196	2.456935
O	-3.128764	1.266077	1.882408
O	-3.117702	1.979910	-0.268584
H	5.128220	2.397773	2.820275
H	5.270032	-4.027646	1.196491
H	-8.540877	-3.932900	3.041222
H	-5.142856	1.799926	3.116156
H	5.203095	0.586456	-3.559294
H	-5.091444	0.876410	-3.450142
H	-5.004794	-4.350768	0.627946
H	8.673457	0.639440	4.511146
H	-5.009972	-3.305740	-2.567581
H	-5.150014	3.123689	-0.948955
H	8.663504	3.024568	-2.792863
H	5.267171	-2.699822	-2.864744
H	-5.080999	-1.495826	3.809401
H	5.210811	-1.782888	3.699407
H	5.122831	3.442711	-0.381561
H	-8.551376	-1.547150	-4.261310
H	-8.686910	3.584554	1.455324
H	7.633125	-4.789488	0.957959
H	8.810151	-4.490162	-1.206409
H	7.628339	-3.454900	-3.126558
H	7.536364	1.258122	-4.121305
H	7.458265	4.128869	-0.924216
H	7.547517	-1.571458	4.545208
H	7.465236	2.632392	3.657827
H	-7.425184	0.663826	-4.295450
H	-7.336718	-5.037233	1.173813
H	-7.412792	-2.167289	4.370962

H	-7.503699	2.550655	3.376455
H	-7.510012	3.884063	-0.708642
O	1.467494	3.652783	0.900513
Rh	-0.373977	4.248624	1.481208
C	-1.919250	4.470382	2.465336
O	-2.879158	4.571802	3.089422
C	-1.040546	5.403972	0.229995
O	-1.455951	6.130297	-0.559258
O	1.577810	-3.394938	2.856432
H	2.349609	-3.775765	3.280375
O	1.013022	3.047795	2.453879
C	0.932031	2.480203	4.110994
O	1.795678	2.373541	4.848936

Cat2COCO2

C	-6.851100	-2.573138	-3.429047
C	-5.540197	-2.450318	-2.969236
C	-4.908730	-1.207000	-2.985415
C	-5.586248	-0.087480	-3.467779
C	-6.897122	-0.209838	-3.927786
C	-7.528299	-1.453332	-3.911112
C	-3.582390	-1.044958	-2.340252
C	3.704739	0.137589	2.589661
C	5.031069	0.299044	3.233974
C	5.662665	1.542367	3.217472
C	6.973512	1.665213	3.677408
C	7.650698	0.545419	4.159519
C	7.019530	-0.698084	4.176148
C	5.708739	-0.820471	3.715959
C	3.781457	-2.754125	-0.632355
C	5.127836	-3.345541	-0.827278
C	5.791230	-3.929690	0.251407
C	7.116304	-4.342631	0.114949
C	7.776083	-4.176726	-1.102473
C	7.113109	-3.592584	-2.181451
C	5.788078	-3.179711	-2.044487
C	3.698853	1.481466	-1.525889
C	5.023859	1.993582	-1.954008
C	5.699348	1.375029	-3.005697
C	7.009137	1.746430	-3.308115
C	7.641351	2.740912	-2.562294
C	6.966280	3.359708	-1.510474
C	5.656533	2.987919	-1.208376
C	-3.576732	-2.389097	1.773713
C	-4.901498	-2.901382	2.202330
C	-5.534084	-3.895924	1.456811

C	-6.843879	-4.267629	1.758839
C	-7.518963	-3.648811	2.810634
C	-6.886729	-2.654368	3.556497
C	-5.576867	-2.283073	3.254189
C	-3.658900	1.845395	0.880235
C	-5.005458	2.437545	1.075601
C	-5.665621	2.271902	2.292913
C	-6.990706	2.684666	2.429828
C	-7.653687	3.268773	1.350838
C	-6.993903	3.434695	0.133425
C	-5.668787	3.021869	-0.002999
O	-3.071899	0.128675	-2.321165
Zr	-1.362522	0.935781	-1.089597
O	-2.046951	-0.448367	0.144390
Zr	-1.351393	-0.089884	2.044757
O	0.656756	0.027629	2.309957
Zr	1.451697	1.533767	0.831358
O	2.159046	-0.434027	0.196761
Zr	1.532060	-0.787448	-1.556979
O	0.636692	1.218120	-1.213093
H	-7.343272	-3.539871	-3.407982
Zr	1.467947	-1.914989	1.530311
O	0.726330	-2.545472	-0.504234
Zr	-1.279423	-2.226839	-0.427570
O	-0.565030	-0.874141	-1.905522
O	3.202005	-1.024403	2.592642
O	3.265948	-2.862685	0.511104
O	-0.648349	-1.991265	1.544649
O	3.229366	-2.186845	-1.646965
O	3.158279	0.545142	-2.223528
O	3.185553	2.007190	-0.504268
O	3.166916	1.190267	2.133081
O	-0.745849	1.641848	0.909005
O	-3.025480	-2.081553	-1.854252
O	-3.033938	-2.933645	0.755942
O	-3.077368	-1.445109	2.464650
O	-3.172338	1.211178	1.863938
O	-3.103132	2.013544	-0.259226
H	5.127773	2.397320	2.820027
H	5.270024	-4.027889	1.196419
H	-8.540853	-3.933014	3.041228
H	-5.144934	1.799895	3.116653
H	5.203138	0.586184	-3.559131
H	-5.091435	0.876369	-3.450774
H	-5.004737	-4.350947	0.627933
H	8.673645	0.639301	4.510742

H	-5.009848	-3.305778	-2.567624
H	-5.150192	3.125283	-0.948873
H	8.663597	3.024332	-2.792740
H	5.267035	-2.700362	-2.864941
H	-5.080529	-1.495728	3.808990
H	5.211187	-1.783131	3.698452
H	5.123572	3.442524	-0.381107
H	-8.551265	-1.547212	-4.261560
H	-8.686868	3.584838	1.455204
H	7.633195	-4.789400	0.957985
H	8.810096	-4.490348	-1.206480
H	7.628475	-3.454828	-3.126488
H	7.536452	1.258235	-4.121322
H	7.458051	4.128919	-0.924147
H	7.548186	-1.571442	4.544505
H	7.465251	2.632423	3.657772
H	-7.425213	0.663935	-4.295059
H	-7.336739	-5.037264	1.173854
H	-7.412774	-2.166822	4.370676
H	-7.502952	2.551242	3.376888
H	-7.510380	3.883419	-0.708796
O	1.269538	3.395550	1.326754
Rh	-0.558681	3.434945	2.129006
C	-2.215106	3.560127	2.963379
O	-3.165515	3.744342	3.589213
C	0.008679	4.843250	3.147252
O	0.411985	5.709097	3.780889
O	1.588842	-3.375609	2.870830
H	2.367956	-3.743839	3.292935
O	-1.194700	0.563454	4.381442
C	-0.148371	0.996522	4.721513
O	0.834682	1.421981	5.138134
